# A village-level cluster randomized controlled implementation trial to measure the effectiveness of a behavioral intervention aiming to reduce women’s exposures to household plastic waste burning in rural Guatemala: study protocol for the Ecolectivos trial

**DOI:** 10.1186/s13063-025-09338-z

**Published:** 2025-12-13

**Authors:** Lisa M. Thompson, Amy E. Lovvorn, Hina Raheel, Mayari Hengstermann-Artiga, Maria Renee Lopez, Alex Ramirez, Dana Boyd Barr, Melinda Higgins, John P. McCracken, Eri Saikawa, Margaret A. Handley

**Affiliations:** 1 Family Care Nursing, School of Nursing, University of California San Francisco, San Francisco, CA USA; 2 Gangarosa Department of Environmental Health, Rollins School of Public Health, Emory University, Atlanta, GA USA; 3 Nell Hodgson Woodruff School of Nursing, Emory University, Atlanta, GA USA; 4 Center for Health Studies, Universidad del Valle de Guatemala, Guatemala City, Guatemala; 5 Department of Environmental Health Science, College of Public Health, University of Georgia, Athens, GA USA; 6 Department of Environmental Sciences, Emory University, Atlanta, GA USA; 7 Department of Epidemiology and Biostatistics, University of California San Francisco, San Francisco, CA USA; 8 PRISE Center (Partnerships for Research in Implementation Science for Equity), University of California San Francisco, San Francisco, CA USA

**Keywords:** Solid waste management, Plastic pollution, Open waste burning, Plasticizers, Air pollution, Implementation science, Behavioral change intervention

## Abstract

**Background:**

Open burning of household waste, especially plastics, is a major but unaddressed environmental and health hazard in countries that lack infrastructure to properly manage waste. This study will implement village-level community working groups that aim to reduce household plastic waste burning and improve health-related quality of life in women of reproductive age in rural Guatemala.

**Methods:**

Using a type 1 hybrid-effectiveness-implementation study design, we will randomize 16 matched-pair rural villages in Jalapa, Guatemala, and randomly select 400 women of reproductive age (25 in each village) who report burning plastic waste as a primary form of waste disposal to participate in the trial. In eight intervention villages, we will conduct 12-week community working groups to implement alternatives to burning plastic that are achievable over the subsequent 9 months. We will use the Behavior Change Wheel and RE-AIM, two implementation science frameworks, and a mixed-methods approach to refine, implement, and evaluate community-initiated interventions that address plastic waste. At baseline, 4 and 12 months, we will measure personal exposures to fine particulate matter and black carbon, and urinary biomarkers of exposure (e.g., bisphenols, phthalates, polycyclic aromatic hydrocarbons, and volatile organic compounds). We will use filter-based 1,3,5-Triphenylbenzene, a known tracer of plastic incineration, to quantify emissions estimates of air pollutants due to plastic burning. Based on plastic waste reductions in intervention villages, we will assess regional impacts of pollutant emissions reduction using a 3D chemical transport model.

**Discussion:**

Our findings will inform community-driven public health actions to develop programs that address this environmental and health hazard. This project has direct benefit not only to those residing in Guatemala, but potentially in other areas where open waste burning contributes to air pollutants both regionally and globally.

**Trial Registration:**

ClinicalTrials.gov NCT05130632 (Trial registration date 10/20/2021).

**Supplementary Information:**

The online version contains supplementary material available at 10.1186/s13063-025-09338-z.

## Introduction

### Background and rationale {6a}

Household air pollution from solid fuel combustion (e.g., wood, crop waste, dung) is a major environmental risk factor in low-resource countries, accounting for an estimated 3.2 million deaths in 2020 [1]. While global advances have been made to replace solid fuels with cleaner fuels and stoves (e.g., liquified petroleum gas, electric and solar stoves) [2, 3], these programs do not address plastic that is frequently burned in household fires, either as kindling added to cooking fires [4] or as a method of disposing of the mounting plastic waste in these settings. Globally, two billion people lack adequate municipal sanitation services, leaving no safe alternative to dispose of household waste, including plastic. In low-resource settings, waste is discarded in informal dumps or burned in outdoor open fires and indoor cooking fires [5]. In Guatemala, 17% of total generated waste is plastic [6]. In rural Guatemala, 71% of households burn waste, including plastic, as a primary means of disposal, principally because rural communities lack any formal recycling or sanitation infrastructure [7].

Open burning of plastic waste in household fires is a critical public health and environmental hazard [8]. The human health effects from exposure to air pollution from plastic waste are understudied. Combustion of plastic releases toxic smoke that contains carcinogenic [9, 10] and endocrine-disrupting [11] compounds. While studies suggest that low levels of exposure to bisphenols and phthalates from plastics disrupt neurodevelopment [12], endocrine [13], and reproductive function [14], personal exposure to these compounds in women of reproductive age who burn their household plastic waste has not been reported in the literature. Furthermore, while there are descriptive studies that characterize the environmental and health harms of plastic waste burning [8], there are no randomized-controlled intervention studies that have evaluated potential personal exposure reductions from plastic waste burning in low-resource settings. Finally, there are no estimates of the effects of a behavioral intervention on reductions of ambient air pollution from household-level plastic waste burning.

Key to the success of many behavioral interventions is the theoretical models that address the enablers and barriers to behavioral change, which can fill the implementation gap between what we know (evidence) and what we do (practice) [15]. One widely used approach to determining key intervention targets and strategies to affect change is the COM-B (“Capability,” “Opportunity,” “Motivation,” and “Behavior”) model, part of the Behavior Change Wheel (BCW). The BCW provides a framework for assessing the conditions affecting key behaviors that must be understood contextually before developing effective intervention strategies. Capabilities are demonstrated by applying necessary skills and knowledge. Opportunities are external social or environmental factors that inhibit or allow change. Motivation is guided by reflective or emotional processes that direct behavior. The COM-B Model is at the center of the BCW framework. Nine intervention functions address gaps in capabilities, opportunities, and motivations that, when implemented correctly and consistently, will lead to sustained behavior changes [16]. The related Theoretical Domains Framework (TDF) is a synthesis of 84 theoretical constructs of behavior change organized into 14 domains [17] that maps directly onto the COM-B model and BCW and provides a more detailed characterization of the factors that affect the behavior(s) targeted by the intervention [18]. The BCW can be used to develop behavioral interventions but also to assess the delivery of implementation strategies for intervention components. Few studies have used the BCW for behavioral change related to community environmental health [19–22], but there is considerable recognition that theory-driven implementation science approaches, such as the application of the COM-B/BCW frameworks, are necessary to identify promising intervention approaches that can be empirically tested [23]. In our prior work [19], we applied this approach to design the intervention for enhancing clean cookstove adoption during our formative research, which was then adapted for use in the Household Air Pollution Intervention (HAPIN) trial [24], which is located in the same region of Jalapa, Guatemala, as our currently described research study.

In addition to applying approaches based on implementation science to environmental problems, methodological approaches are needed to address the underlying drivers of pollution prevalent in countries in the Global South facing centuries of colonization [25]. The subsequent structural and political marginalization of indigenous groups, as is the case in Guatemala, requires methodologies that prioritize community voices that have been historically silenced [26]. Community-level engagement is critically important, particularly in rural settings in Guatemala, where 40–60% of the people are indigenous [7, 27], many of whom have been excluded from institutions, laws, or policies that protect them from environmental harms [28]. There are examples of citizen participation to protect the environment in Guatemala, including the community development councils (COCODEs) who represent village concerns, especially in indigenous areas of Guatemala where plastic waste is a recognized problem. In 2024, twenty-three of the 340 Guatemalan municipalities had banned single-use plastic, although these bans are not strictly enforced [28]. Addressing systemic problems of pollution builds necessarily on qualitative and/or mixed methods [29], environmental protection [30], health communication [31], peer education [32], and participatory research [33]. Feasible, implementable, and sustainable alternatives to the mounting plastic waste problem need to be prioritized in these communities.

No intervention studies have assessed personal exposures to plastic combustion by-products, including bisphenols and phthalates, in women of reproductive age. To test this, the Ecolectivos study aims to introduce a behavioral educational intervention (the community working group) and measure the effect on urinary biomarkers of exposure and women’s personal exposure to air pollution. Guided theoretically by two implementation science frameworks, the COM-B model and the RE-AIM Framework [34, 35], we will evaluate the adoption and sustainability of strategies to refuse, reduce, reuse/reintegrate, repair, and recycle plastic instead of burning household-level plastic in intervention communities in rural Jalapa, Guatemala.

### Objectives {7}

Our study aims are to:

1. Implement a behavioral intervention (12-week community working groups) and evaluate strategies chosen by the group that address plastic waste burning, targeting barriers and enablers identified within the capability, opportunity, and motivation components, for key behaviors. The implementation outcomes assessed over 9 months will be reach, effectiveness, adoption, and maintenance/sustainability (*implementation aim*).
2. Evaluate the longitudinal effect of a behavioral intervention (community working groups) on urinary biomarkers of exposure to plastic waste burning (bisphenols, phthalates, polycyclic aromatic hydrocarbons (PAHs) and volatile organic compounds (VOCs)) and personal airborne fine particulate matter (PM_2.5_) and black carbon (BC) in 400 reproductive age women drawn from 8 intervention compared with women in 8 control villages (*effectiveness aim*). We hypothesize that urinary biomarkers and air pollution exposures will decrease over time in 200 women from intervention villages compared to 200 women from control villages at 4–5 and 12–13 months. There are no known estimates of changes over time in personal exposure from an intervention to reduce plastic burning. The expectation of detecting small to moderate effect sizes (Cohen’s *f* = 0.15–0.20) is supported by our previous findings of 20–40% decreases in urinary metabolites of PAHs and VOCs and 57–87% decrease in PM_2.5_ in women who used cleaner cookstoves instead of biomass stoves [36, 37].
3. Using 1,3,5-Triphenylbenzene (TPB) as a tracer of plastic burning [38], and measurement of household plastic waste, apportion PM_2.5_ and quantify emissions estimates of air pollutants from plastic incineration and assess potential emissions reduction from the working group intervention on air quality with a chemical transport model.

### Trial design {8}

Ecolectivos is a village-level cluster randomized controlled trial (cRCT) using a type 1 hybrid effectiveness-implementation study design that measures the effectiveness of the community working group, while also evaluating how the intervention is implemented [39, 40].

See Additional File 2 for the SPIRIT Checklist used during the development of the protocol.

## Methods: formative research

### Patient and public involvement

Community-dwelling research participants were involved in the co-production of the behavioral intervention described in the formative phase before the trial. We conducted formative research in Year 1, which allowed members of the targeted communities to be actively involved in activities that led to the ultimate design and conduct of the trial. We describe here the formative research that was already carried out to illustrate the activities that prepared us for the trial. Formative research consisted of four main components: (1) form a community advisory board (CAB); (2) conduct a baseline assessment survey; (3) refine the behavioral intervention, the working group, including conducting a pilot working group in one community (Aim 1); and (4) pilot exposure assessment methods for the main trial (Aim 2).

### Study setting {9}

The formative research was conducted in the rural mountainous region of Santa Maria Xalapán in Jalapa, Guatemala, a region where we have conducted research since 2017 as part of the HAPIN trial [41–43]. The Jalapa Department has a rural population of approximately 75,000 people in 133 villages, with 37 of these villages in the Santa Maria Xalapán district. In this region, almost 50% self-identify as Xinca indigenous people; this proportion is higher in rural areas [7]. The remaining population self-identify as *Ladino* or *mestizo*. In our earlier pilot work, 84% of women reported that they were responsible for burning their household waste, including plastic [44], thus justifying an intervention study among women in this study setting.

#### Eligibility criteria for formative phase

For the baseline survey and pilot working group, the inclusion criteria were as follows: (1) age > 18 years; (2) lives in the study area; and (3) head of household and primary cook. Among the baseline assessment survey participants, we selected 12 households that reported burning plastic waste inside and outside of their homes to participate in a pilot of air pollution monitoring that would inform the trial monitoring strategies. For CABs and key-informant interviews, inclusion criteria were as follows: (1) willing to attend quarterly CAB meetings and (2) identified as someone who works for a non-governmental or governmental organization on environmental protection or improvement, such as recycling, or a village leader.

#### Community Advisory Board

We invited 12–15 people to form a CAB, consisting of community stakeholders (e.g., councilmen from local villages, organizations working on waste management, recyclers, and governmental agencies (e.g., such as the Ministry of Natural Resources and Ministry of Health)). The CAB was designed to provide input on study activities, evaluate working group intervention strategies, and discuss the potential to expand and sustain activities regionally. We extended formal invitations and met with individuals, but we were unsuccessful in formalizing a CAB that would guide the trial activities. Reasons for not joining the CAB included the following: individuals saw the CAB as a vehicle for personal gain, one member asked for large donations (e.g., a truck) for their community-based organization, those who worked for governmental agencies told us they were not authorized to work on projects that were outside their job scope, community leaders informed us that they hold their elected positions for a fixed term and could not commit to a 5-year membership, and Xinca indigenous leaders worked as a collective and did not want a single person to represent a communal voice. There was also tension and distrust among members representing the government, who were from urban Jalapa, and the indigenous leaders who represented their communities on the Santa Maria Xalapán mountain. Given these challenges, and based on consultation with local leaders, we chose a different path for the trial. We were asked to meet with leaders (known as the *Juntas*) in each of the selected communities to explain the goals of our study, and to seek their approval before we started. We met with them during randomization, and then periodically during the study to inform them of the progress. We met with the COCODE, who are indigenous members who coordinate municipal programs, and serve as a bridge between municipal and local authorities. Without their permission, the study would not have been allowed to proceed.

#### Baseline survey

The second formative phase activity was to conduct a baseline assessment of households in the Santa Maria Xalapán region. Using Google Earth imagery, geographical software, and the 2018 Guatemala Census to define a sampling frame and digitize building structures, we randomly selected 60 rooftops using satellite images from 37 selected villages with more than 200 households, oversampling to account for 30% misidentification of structures for a total of 2220 potential households. Trained local fieldworkers used hand-held GPS receivers to identify 1630 households. They verbally consented and administered a 30-minute survey to the primary cook about the following: (a) household size/composition; (b) stove type(s) and location(s); (c) location and frequency of burning waste, including plastic; (d) other types of waste that are burned in and outside of the home; (e) recycling practices; (f) capabilities and opportunities to dispose of waste besides burning; (g) motivation to improve the environment; (h) interest in participating in future community working groups; and (i) contact information. This brief Spanish-language survey was constructed based on information from a previous pilot study conducted in the same area (unpublished). It was formulated in Spanish and tested with local fieldworkers for consistency and understandability. We did not conduct reliability or validity testing since this was not a scaled, scored instrument but rather an attempt to characterize household characteristics and women’s perceptions about plastic waste before introducing a behavioral intervention. The main purpose of this activity was to identify potential, eligible participants from these households and assess their interest in participating in the future trial.

#### Refinement of intervention component: community working group

In the third formative phase activity, we piloted and refined the community working groups in two villages, and tested potential intervention strategies, like soap-making, recycling, and composting, that could be implemented. We refined the curriculum and tested all procedures in two villages to standardize the scripts and materials. To refine the curriculum, we observed participants who burned plastic waste in their households and conducted open-ended surveys with them. We conducted key informant interviews with community stakeholders who recycled or repurposed plastic.

#### Pilot air pollution monitoring and urinary biomarkers

The fourth strategy of the formative phase was to test the exposure assessment methods for the trial. We recruited 12 households identified in the baseline assessment who reported burning plastic waste in household fires. We monitored 24-h personal and kitchen concentrations of personal airborne 24-hour fine particulate matter (PM_2.5_) and black carbon (BC) and measured open waste burning over 1–2 hours. To assess two different tracers of plastic combustion, we measured filter-based 1,3,5-Triphenylbenzene on 37-mm quartz filters and antimony (Sb) on 37 mm polytetrafluoroethylene (PTFE) filters [45–47]. We collected first-void urine samples from the 12 women at the 24-hour visit when we retrieved the air pollution equipment. Frozen urine was shipped to the LEADER Laboratory at Emory University and analyzed for analytes of 17 phthalates, 7 PAHs, and 2 bisphenols using liquid chromatography-tandem mass spectrometry (LC–MS/MS) as described elsewhere [48, 49].

## Methods: participants, interventions, and outcomes

### Study setting {9}

The trial will be conducted in the same area as the formative research. Among the 37 villages in Santa Maria Xalapán, 16 villages (8 intervention, 8 control) will be recruited to participate. In our earlier pilot work, 84% of women reported that they were responsible for burning their household waste, including plastic [44], thus justifying an intervention study among women in this study setting.

### Eligibility criteria {10}

Women are eligible to participate in the trial if they meet the following inclusion criteria: (1) of reproductive age (15 to < 44 years, verified by an official document); (2) willingness to attend weekly meetings of the 12-week community working groups if the village is randomized to the intervention group; (3) willingness to participate in the exposure/biomonitoring study; (4) reported use of biomass as the primary fuel for cooking; (5) reported daily participation in household cooking (does not need to be the primary cook); and (6) reported plastic burning in household fires (in cooking stove and outdoors) at least once a week. Exclusion criteria for women are as follows: (1) inability to consent; (2) individuals with impaired decision-making capacity; (3) currently pregnant; and 4) report using tobacco products.

To be eligible to be an environmental health promotor (EHP), participants must have participated in the community working group, be over the age of 18 years, and know how to read and write. For invited participants who attend the community-level working groups, there are no inclusion or exclusion criteria, but questionnaires administered to attendees will be limited to those over the age of 15 years.

### Who will take informed consent? {26a}

Written informed consent or assent for all procedures, including ancillary studies, will be obtained by trained local fieldworkers in Spanish, the language of the Xinca indigenous people. Fieldworkers receive training in the ethical conduct of research from the Universidad del Valle de Guatemala (UVG), including how to obtain informed consent and how to protect the confidentiality of data collected throughout the study. The field team will be supervised by the local principal investigators and re-trained periodically to ensure standardization of procedures. Steps will be taken to ensure participants’ comprehension at an elementary-school level. Written assent will be obtained from participants between 15 and < 18 years of age, and a designated legal guardian will also provide consent for assenting individuals who choose to participate in the study. Informed consent will be signed by participants who can read and write. For those who cannot read or write, the fieldworker will read the informed consent, and in the presence of a witness, the participant’s thumbprint will serve as valid consent. The study will employ standard methods for protecting the confidentiality and privacy of participants based on the ethical principles of the Declaration of Helsinki.

### Additional consent provisions for collection and use of participant data and biological specimens {26b}

Biological samples will be collected using the informed consent described above. Collected urinary specimens will not be used for other purposes than those described here.

## Intervention

### Explanation for the choice of comparators {6b}

Based on our formative baseline assessment survey (data unpublished) and Guatemalan census data [7], we will randomly select eight pairs of non-contiguous villages matched on population size, proximity to a main road, and distance from the city of Jalapa. Matching villages based on proximity to a main road and to the city of Jalapa is necessary because garbage and recycling vehicles can access households near main roads, potentially offering an alternative to household open waste burning as a means of disposal. To minimize contamination (from both air pollution and spill-over of intervention activities) between intervention and control groups, we will select non-contiguous villages. Control villages are the comparator group and will not receive the behavioral intervention.

We justify matching villages on these criteria based on our formative baseline survey (*n* = 1572 randomly selected participants surveyed in 37 villages), which showed important distinctions between women living in villages located on main, paved roads, where women were younger, more educated (*p*-value ~ 0.08), and had more “large assets” than women living in more remote villages accessed by dirt roads. While only 1.3% of all 1572 households reported municipal/private company waste pickup, the households near the road were statistically more likely to state that recycling would benefit them (*n* = 474; 89%) versus those living at a distance from a road (*n *= 501; 85%) (*p*-value = 0.031) and stated that it would be easier for them to stop burning plastic trash (*n* = 235; 36%) compared to those living at a distance from a road (*n* = 255; 28%) (*p*-value = 0.004). We did not find studies in similar rural settings that test the assumption that households along a main road would be more likely to access waste management services or recycle than households distanced from main roads. However, rurality [50] and poor roads [51, 52] have been found to be barriers to proper waste management, leading to clandestine dumping and complicating the logistics of recycling in rural remote settings.

### Intervention description {11a}

The community working group is a behavioral intervention with the goal of enabling participants to engage in strategies that lead to the reduction or elimination of household plastic waste burning. The COM-B model is used in the intervention materials (e.g., by participants using take-home worksheets, by researchers using evaluation materials (see Supplementary Materials: Materials for community working groups) to identify and subsequently address the capabilities, opportunities, and motivations to stop burning plastic waste that form the behavioral intervention).

Participants over the age of 15 from 8 intervention villages will be invited to participate in 12-week working group sessions in a local village meeting place (Table 1). Participants from the 8 control villages will not participate in the working group sessions, but they will have the same scheduled study visits for household data collection as the intervention participants. We will seek permission from village leaders and ask for their support in promoting working group activities, since from our experience they are decision-makers about village-level change. Two to four local Guatemalan fieldworkers will implement the 12-week working group, which is grounded in the ecological and health implications of exposure to air pollution from burning plastic waste. The Spanish-language manual (and English translation) used to develop the modular materials (PowerPoints, handouts, and other didactic materials) is available on the Ecolectivos website [53]. Eight core modules (weeks 1–8), the “essential ingredients,” and four additional modules (weeks 9–12) will be implemented weekly. During weeks 9–12, participants will identify intervention activities to reduce plastic waste burning. Participants will brainstorm on individual and community strategies that are important to them and will prioritize one or more strategies that can realistically be implemented over the next 9 months, under the guidance of Ecolectivos fieldworkers and designated EHPs. These strategies may consist of the following, for example: (1) conducting community-level clean-up campaigns; (2) starting organic compost free of plastic waste; (3) training on community recycling, focusing on plastics; (4) making organic soaps, which can be used for personal grooming and washing clothes, eliminating plastic packaging; and (5) creating materials out of plastic, like soft drink bottle planters.

**Table 1:** Working group curriculum themes and components

Week	Theme	Components
1	Identification of main problems of solid waste management, including sources of contamination in communities	Uses of plastics, plastic in waterways, plastic burning, introduction to recycling *Homework:* Turn in pre-test; tally amount of plastic in the household
2	Personal, family, and community practices of plastic waste management and effects on the ecosystem	Discussion of sources of contamination in the ecosystem and ways to reduce plastic use *Homework:* Plastic production and marine life
3	Plastics in waterways and oceans, including discussion of microplastics; introduce the 4 R’s (refuse, reduce, recycle, repurpose)	*Group activity:* Make a stool out of plastic bottles and fabric *Homework:* Time needed to degrade plastic and other materials in the environment
4	Contaminants in burning plastic and health implications of exposure; discuss the difference between junk food in plastic bags and food like fruit, and vegetables, typically not wrapped in plastic	Discussion of alternatives to burning plastic; video on junk food versus healthy food *Group activity:* Make worm composting bins *Homework:* Find substitutions for plastic items used in the home
5	Sustainable alternatives to plastic and reducing plastic litter in the community	*Group activity:* Make organic soap (plastic-free) *Homework:* Specify type and amount of plastic used during the week
6	Recycling plastic; discuss materials that can and cannot be recycled	*Group activity:* Sort recyclable plastic materials; Recycling company buy materials *Homework:* Find recyclable items in home
7	Environmental justice; community empowerment	*Homework:* Identify community problems that need to be addressed
8	Collective action; importance of community groups; describe plastic ban in Guatemala	*Group activity:* Community clean-up *Homework:* Administer post-test; reflect on activities that participant wants to work on
9–12	Group selection of 1–2 community activities, or “interventions”; this includes weekly meetings with Ecolectivos fieldworkers and community stakeholders who can support these activities. Project resources will be dedicated to implementing the activities, addressing bottlenecks to success	

### Criteria for discontinuing or modifying allocated interventions {11b}

Participants are free to discontinue their participation in the intervention for any reason, at any time. These reasons will be documented on an exit survey form. We are not monitoring clinical outcomes that would require modification of the allocated intervention.

### Strategies to improve adherence {11c}

Ecolectivos fieldworkers and EHPs will work with community members to overcome bottlenecks to implementation during this period. We aim to maintain adherence to core modules through strict protocols, while permitting flexibility of the selected activities, to allow integration of prioritized activities into community practices over the 9-month period [54]. Strategies to improve adherence to the behavioral intervention will include phone calls and home visits by our field team and EHPs to encourage working group attendance, use of an attendance card stamped at each visit to monitor attendance, and in-class and take-home assessments about plastic waste, including waste burning in household fires.

### Concomitant care permitted or prohibited {11d}

Concomitant interventions or procedures, should they exist, are allowed during the study. There are no prohibitions on concomitant clinical care because this is not a clinical trial.

### Outcomes {12}

Outcomes will be measured at baseline (before randomization), at 4–5 months and 12–13 months (follow-up) in women who participate in the exposure/biomonitoring study. The follow-up period for both intervention and control participants included in this study will be 12–13 months. Each matched community pair will be followed during the same period to control for seasonal and secular trends in local migration to harvest coffee, agricultural waste burning, and plastic waste burning.

### Primary outcomes

Our primary outcomes are changes from baseline in personal airborne 24-hour fine particulate matter (PM_2.5_) and black carbon (BC) and changes in urinary concentrations of bisphenols (BPA’s), phthalates, polycyclic aromatic hydrocarbons (PAHs), and volatile organic compounds (VOCs) (effectiveness, Aim 2).

#### Particulate matter and back carbon 

We will use air pollution sampling techniques employed in previous studies [36, 42, 55]. We will assess exposures to personal airborne PM_2.5_ and BC in all 400 women using PTFE 37 mm filters. We will use a personal monitor placed in apron pockets that we provide to participants. We will instruct women to wear the apron throughout the day and to place it near their bed at night. On day 2, we will retrieve the equipment and administer a survey about daily activities/compliance. We will use the Ultrasonic Personal Air Sampler monitor (UPAS V2 Plus, Access Sensor Technologies, Fort Collins, CO), which provides real-time and filter-based PM_2.5_. The UPAS is lightweight, quiet, durable, and has a battery that lasts over 24 hours. The device uses a cyclone for PM_2.5_ size separation and a pump that maintains a constant flow rate, measured internally using pressure sensors. The device is configured over Bluetooth using an iPhone app [56].

Prior to deployment in the field, PTFE filters will be conditioned and pre-weighed in the air pollution laboratory at the Universidad del Valle de Guatemala using a microbalance (Sartorius Cubis MSA 6.6S-000-DF-00) to calculate the gravimetric PM_2.5_ concentrations. A SootScan™ (Model OT21 Optical Transmissometer) will be used to analyze BC. Each week, one lab blank sample will be collected. After sample collection, PM_2.5_ mass will be calculated as the difference between pre- and post-sampling filter weights, each determined in duplicate. PM_2.5_ masses will then be converted to mass concentrations by dividing by the sampled air volume. Mean change in field blank filter masses (5% of samples will have field blanks) will be subtracted. We will measure precision by performing duplicates in 5% of samples. Black carbon will be determined by the optical attenuation and black and brown carbon mass. The analysis is non-contact, non-contaminating, and non-destructive. Therefore, filters can be subsequently analyzed for additional elements. The analysis method requires no support gases or consumables.

#### Bisphenols, phthalates, polycyclic aromatic hydrocarbons, and volatile organic compounds 

We will use urine measurement methods that were successful in our previous studies [36, 43, 55]. On day 1, when air pollution exposure sampling begins, female fieldworkers will instruct women on clean-catch procedures and provide a sterile urine collection cup and vaccine cooler with ice packs. Women will be instructed to collect a first-morning void sample on day 2. During the home visit on day 2, fieldworkers will administer surveys, including urine collection time. Labelled specimens will be stored in a cooler and transported in project vehicles to the field laboratory. Urine will be frozen within four hours of collection.

In the field laboratory, 21 ml of urine will be transferred to four cryovials (two 4 ml tubes with 3 ml urine and two 10 ml tubes with 7.5 ml urine) using QR code labels that include sample identification information including participant ID, the phase of the study (baseline, 4 months, and 12 months), matrix, and aliquot number. Samples will be stored at −20 °C in the field laboratory and transported to the UVG laboratory within 3 months. They will be stored in −80 °C freezers at the UVG laboratory until shipped with freezer packs to the LEADER Lab at Emory where they will be stored at −80 °C until urinalysis is performed. We will not include information about the study arm so that blinded data will be analyzed. If samples are accessed by investigators other than those named in this protocol, data will be requested by the investigators using a data request form and reviewed by the MPIs before approving any requests.

**Fig. 1:**
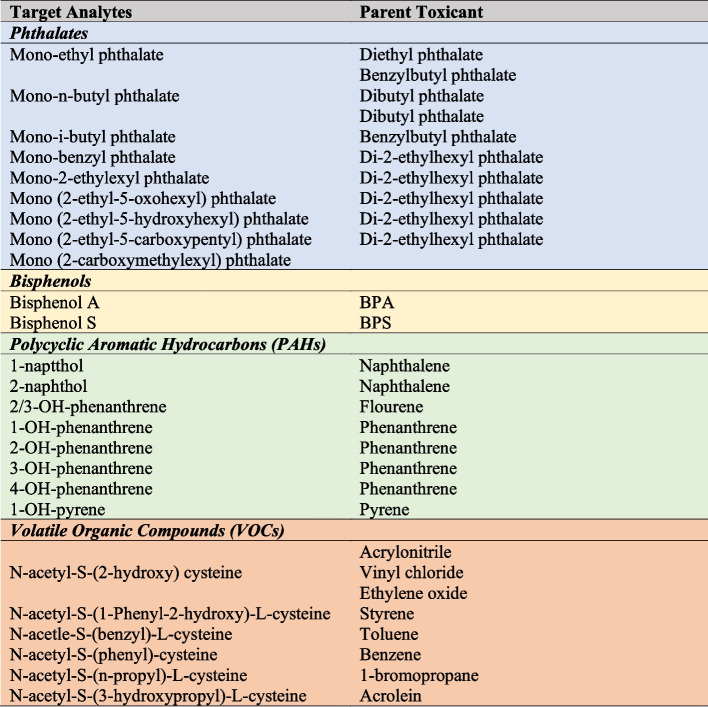
Target analytes and parent toxicants

Target analytes are outlined in Fig. 1. Eight PAH and six VOC analytes will be targeted. 1.0 ml (PAHs) and 0.5 ml (VOCs) of urine will be aliquoted and spiked with isotopically labeled internal standards for automatic recovery correction and normalization of mass spectral data. Nine phthalate and two bisphenol analytes will be targeted. A 1.0 ml aliquot of urine spiked with isotopically labeled analogues of target phthalates and phenols will be subjected to enzyme hydrolysis to liberate glucuronide-bound conjugates. The concentrated extracts will be analyzed using GC-MS/MS. Two quality control materials (one high and one low) and one blank sample will be analyzed concurrently with each set of 28 unknown samples, and NIST reference standards will be used for quality assurance measures.

### Secondary outcomes

Our secondary outcomes are implementation outcomes (Aim 1) and include reach, effectiveness, adoption, and maintenance/sustainability of the intervention using the RE-AIM implementation science framework:

1. Assess the reach of the intervention in the intervention arm, as measured by the following: (1) the number and proportion of total invited participants who attend working groups; (2) the number and proportion of participants' household members who engage in working group activities; and (3) the number of intervention village members who engage in working group activities.
2. Assess the effectiveness of the intervention by measuring the number of intervention group participants who report behavior changes. These changes are categorized as “high” or “low” behaviors based on factors such as working group attendance, engagement in activities, and reported reductions in plastic burning.
3. Assess the enablers and barriers to the adoption of the intervention using qualitative data collected from focus groups and interviews with participants and EHPs. This will help identify the factors influencing the uptake of the intervention at the village level.
4. Assess the adoption of the intervention through direct observations of intervention activities, using qualitative data analysis to evaluate the extent of implementation at the community level.
5. Assess the maintenance (sustainment of intervention strategies) at the participant, household, and village level, as measured by the following: (1) number and proportion of participants who report no longer burning plastic in household fires (a measure of de-adoption); (2) number and proportion of households who continue their involvement in intervention activities at the village level after the working groups are completed; and (3) number of community members and organizations who have independently maintained, or established, similar intervention activities in the communities after the working groups are completed in the intervention arm.

The RE-AIM evaluation framework is elaborated upon in Table 2.

**Table 2:** Exemplar of COM-B and RE-AIM frameworks to evaluate individual and community-level behavior change

Examples of COM-B Inquiry	Effectiveness outcomes	Reach, adoption, and maintenance outcomes
Psychological/physical capability		
Identify plastic items in the home. (TDF^1^ domain = *Knowledge*)	*N* and % participants separating plastic and other recyclables correctly	% households report recycling by material type (*adoption*); variation in recycling by village (*reach; maintenance*)
Have you burned household waste? Plastic? (TDF domain = *Knowledge*)	*N* and % participants stating benefits from not burning plastic waste	% households de-adopting burning of plastics (*adoption*)
If you started an activity (like recycling or community clean-up), what would you need to be successful? (TDF domains = *Knowledge; Memory, attention & decision processes; Environmental context & resources*)	*N* and % participants still participating in the activity	% still participating in the activity (*adoption)*; variation in participation by village (*reach; maintenance*)
If your household stopped burning waste/plastic, what would happen? (TDF domain = *Outcome expectancy*)	*N* and % participants no longer burning plastic in indoor fires/in outdoor fires	% of villages no longer burning plastic (*adoption)*; variation in burning by village (*reach; maintenance*)
Reflective/automatic motivation		
Have you talked with your family members about not burning plastic? What have they said? (TDF domain = *Social influences*)	*N* and % participants reporting that participation in educational sessions has improved their status within the home	Variation in perceptions by village (*reach; maintenance*)
Have you seen less plastic waste? Less plastic waste burning in your community? (TDF domain = *Optimism; Beliefs about consequences; Emotion*)	*N* and % participants who perceive less waste present/burning as part of community goals	Variation of villages attributing stopping burning waste for community goals by village (*reach; maintenance*)
Does burning plastic harm health? What happens if we don’t take action to reduce plastic? (TDF domain = *Beliefs about consequences*)	*N* and % participants reporting health consequences from exposure to burning plastic	Variation of villages attributing stopping burning waste to health outcomes by village (*reach; maintenance*)
Is recycling a priority? Is not burning waste/plastic a priority? (TDF domain = *Social influences; Goals*)	*N* and % participants and community members active during community clean-ups	Variation in villages participating in a municipal waste program and reduced waste burning *(reach; maintenance)*
Physical/social opportunity		
What community resources are needed to have a village “free of plastic”? (TDF domain = *Environmental context and resources*)	*N* and % accessing materials needed to create an improved environment in their village	Variation in villages accessing materials (e.g., recycling bins, clean-up resources, etc.) *(adoption; reach)*
Do people in your community care about waste or waste/plastic burning? (TDF domain = *Social influences; Goals*)	*N* and % stating others have reduced waste/plastic burning	Variation in reporting by village (*adoption*), and by age, gender, etc. (*reach*)

^*1*^*TDF* Theoretical Domains Framework

### Other outcomes

Other outcomes include quantification of emissions estimates of air pollutants from plastic incineration using filter-based 1,3,5-Triphenylbenzene (TPB) as tracers of plastic burning and collecting household plastic waste (Aim 3). Because engagement in a community working group intervention may affect women’s sense of well-being, we will also measure changes in Health-related Quality of Life Score, Household Decision Making Score, New General Self-Efficacy Scale, Short Social Capital Assessment Tool Score, and Community Mobilization Scale Score.

*1,3,5-Triphenylbenzene (TPB):* In a subset of 50 women, we will measure 1,3,5‐TPB and a series of 25 PAHs on 37 mm quartz filters twice (50 at baseline and 50 at 4 months; 25 each from intervention and control). The filters will be pre-baked at 550 °C at the Stone Research Group at the University of Iowa. The filters will be analyzed as done in previous studies [57]. Extracted samples will be analyzed by gas chromatography (GC; Agilent Technologies 7890 A) coupled to mass spectrometry (MS; Agilent Technologies 5975). All measurements will be blank-corrected.

*Ambient monitoring: *With the permission of the Guatemalan Ministry of Health officials, we will set up low-cost PM_2.5_ ambient sensors (MODULAIR-PM, produced by QuantAQ, Inc, Somerville, MA) on the rooftops of four health centers in participating villages, selected for their distance from main roads and stable electricity supply. A fifth monitor will be located on the field station in Jalapa city. The MODULAIR-PM measures size-resolved particle mass concentrations and particulate matter (PM) at different diameters, as well as temperature and relative humidity. Data is transmitted via cellular phone data services and can be viewed globally via the QuantAQ Cloud platform. Before setting up the sensor, all five sensors are co-located at the Georgia Environmental Protection Division’s South DeKalb monitoring station to compare with the data collected by the Federal Equivalent Method (FEM). We will develop a bias-correction model using the hourly and daily average values over the 1-month co-location period. Such data is essential for air quality management and to understand sources to mitigate pollution.

*Estimates of plastic waste burning:* From each village, we will randomly select 5 participating households and collect all nonorganic waste, including plastic. We will instruct them to collect all household waste produced over a 1-week period, at baseline, 4 months, and 12 months, in bags we provide. After fieldworkers sort and weigh the plastic waste at the field office, waste will be transported to the municipal dump and/or the recycling center in Jalapa City. These estimates will be used to capture changes in plastic waste that would have been burned and used for modeling plastic emissions estimates in Aim 3.

*Women’s health and well-being:* To measure women’s sense of well-being, we will utilize the Health-related Quality of Life instrument (HRQoL-4) [58], Household Decision Making Scale [59], New General Self-Efficacy Scale (NGSE) [60], Short Social Capital Assessment Tool (SASCAT) [61], and the Community Mobilization Measure (CMM) [62] (Table 3). During the formative phase, we piloted all surveys for understandability and face validity using cognitive interviewing in 10 households purposefully selected to represent the intended sample for the main study.

**Table 3:** Study instruments to measure women’s health and well-being

Instrument	What it measures
CDC Health-related Quality of Life (HRQoL-4) instrument [58]	4 item Healthy Days core questions: 1) would you say that in general your health is excellent, very good, good, fair, or poor?; 2) for how many days during the past 30 days was your physical health not good?; 3) for how many days during the past 30 days was your mental health not good?; and 4) during the past 30 days, how many days did poor physical or mental health keep you from doing your usual activities?
Household Decision Making [59]	Household decision making will be measured by a household decision making scale. This scale utilizes 7 questions, and the responses are coded on a 4-point scale: 1 = Husband; 2 = Joint decision; 3 = Respondent decision; or 4 = Others
New General Self-Efficacy Scale (NGSE) [60]	Self-Efficacy will be measured using the validated New General Self-Efficacy Scale. It utilizes an 8-item scale that is measured by a 5-point Likert scale, ranging from 1 = strongly disagree; 2 = disagree; 3 = neither agree nor disagree; 4 = agree; 5 = strongly agree
Short Social Capital Assessment Tool (SASCAT) [61]	Community social capital will be measured using a validated measure, the 9-item tool, which assesses group membership, support from others, citizenship activities (yes/no responses), and cognitive social capital (yes/no responses)
Community Mobilization Measure (CMM) [62]	Community mobilization will be assessed using two subscales (collective action, 3 items; and critical consciousness, 9 items) from the validated community mobilization measure. The responses are noted on 3-point Likert scale: agree, somewhat agree, disagree

The HRQoL-4 instrument is a widely used, well-validated instrument developed by the Centers for Disease Control and Prevention (CDC) used in the Behavioral Risk Factor Surveillance System (BRFSS) and the National Health and Nutrition Examination Survey (NHANES) surveys with multi-racial/ethnic groups. Studies have shown that responses are strongly associated with self-reported health conditions, as well as chronic diseases and depression [63]. We will use the Spanish version of the HRQoL-4 developed by the CDC. The household decision-making instrument has been used globally in Demographic and Health Surveys to assess women’s autonomy [64]. The short form of the survey contains 4 core questions about who decides about women’s health care, women’s visits to family or friends, and large and small household purchases. We will use the expanded 8-question survey, which asks additional questions about decisions regarding women’s seeking work outside of the home, spending her or her partner’s earned money, and what foods are cooked [65]. We added one unique question about decisions regarding trash disposal. The New General Self-Efficacy Scale (NGSE) has excellent internal consistency (Cronbach’s *α* ranging from 0.86 to 0.90) and content, construct, and predictive validity. The NGSE has been correlated with motivational traits like self-esteem and desire for achievement [60]. We translated the scale into Spanish and back translated it into English; two bilingual researchers independently compared the version for consistency. Based on the cognitive interviews, we added two questions: “give me an example of a goal that you have set for yourself” and “give me an example of an outcome important to you.” The developers of the Short Social Capital Assessment Tool (SASCAT) used psychometric methods and cognitive interviewing to measure citizenship (participation in activities), social and cognitive capital in Vietnam and Peru [61]. The SASCAT had good face and fair discriminant validity; the author recommended that cognitive interviews be used to pilot and adapt the tool in different settings [66]. Based on our cognitive interviews, we removed one question: “do you think that the majority of people in this community would try to take advantage of you if they got the chance?” because it was found to be offensive. We added three questions specific to participant engagement in the Ecolectivos activities. Finally, the Community Mobilization Measure (CMM) measures women’s perceptions about the extent to which communities mobilize around specific issues. It assesses collective actions of community members facing common issues of concern via six domains: shared concerns, critical consciousness, leadership, collective action, social cohesion, and organizations and networks. Internal consistency reliability was strong (*ρ* = 0.81 to 0.93) and subscales were correlated with other measures of social capital, with subscales demonstrating both individual and community-level validity [62]. We used the 9-item critical consciousness subscale, which queries how people solve village problems together, and the 3-item collective action subscale, which ascertains participation in community events. We added a question about specific attendance at Ecolectivos activities.

### Participant timeline {13}

**Fig. 2:**
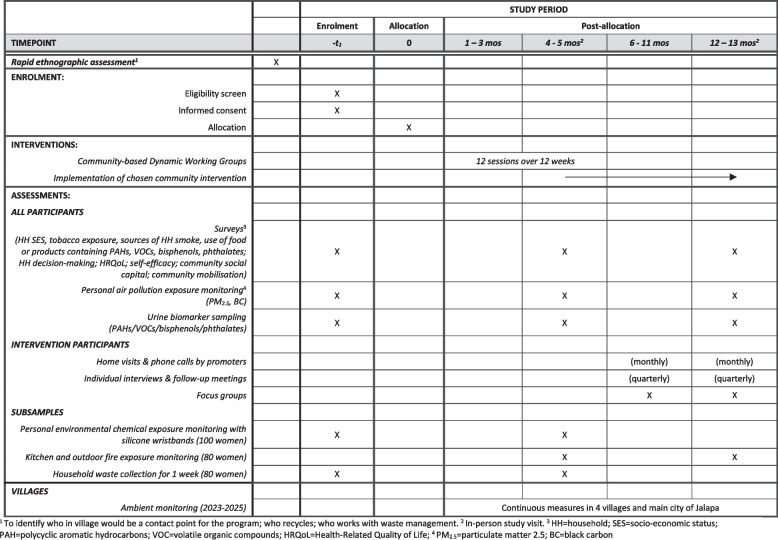
Ecolectivos trial schedule of enrolment, interventions, and assessments

Trial recruitment is staggered over a 2-year period as each village pair enters the study. All measures will be collected from 400 women who participate in the trial at baseline (before randomization), at 4–6 months (timed to coincide with the conclusion of the 12-week community working group in the intervention villages), and 12–13 months from baseline (timed to coincide with the conclusion of the 9-month intervention activities). Fieldworkers will make household visits at these three timepoints. Each household visit will occur over two days to sample air pollution and collect urine specimens. See Fig. 2 for a schedule of assessments during each visit.

### Sample size {14}

Our final sample size is estimated at 400 (200 in each group) and is based on differences in urinary metabolites by study arm. Of the 400 women recruited for Aim 2, we estimate 10% will drop out by 12 months, or 360 participants. With the lower sample size of 360, we will be powered to detect small effect sizes on urinary metabolite differences (Cohen’s *f* = 0.15–0.20) for the group, time, and group-by-time effects in a repeated measures analysis of variance for 8 groups and 3 time points. It is expected that the lower bound sample size of 360 (180 in each group) will account for both attrition and intermittent missing data as well as the potential for non-independence of households clustered within villages, which will be measured by intraclass correlation coefficients (ICC). Although the correlation in exposure among women within the same village tends to reduce the power for between-group comparisons, we expect substantial gain in power by randomization within pair-matched communities based on factors that are predictive of exposure as well as adjustment for baseline differences [67]. The expectation of detecting small to moderate effect sizes is supported by our previous findings for changes in urinary metabolites of PAHs and VOCs yielding significant decreases of 20–40% from baseline [36, 37].

We will ask women at baseline, 4 and 12 months, “do you burn plastic waste in your home?” We assume that in control households, there will be a 5% decrease in burning plastic waste. Using a cluster randomized design and estimated intraclass correlation coefficients (ICC) ranging from 0.05 to 0.10, we would be able to detect a 30% reduction in plastic burning in households in the intervention villages with 80% power, an alpha level of 0.05 and a sample size of 255 (ICC of 0.05, design effect of 2.2) or 394 (ICC of 0.10, design effect of 4.4).

### Recruitment {15}

We will recruit 400 women of reproductive age from 16 villages from the baseline assessment conducted in the formative phase. Trained local Guatemalan fieldworkers will re-visit the women who expressed interest in participating in the trial during the formative research survey. If women are not home after making three visit attempts (e.g., due to a permanent move or temporary migration), or do not wish to consent to participate in the study, the fieldworkers will choose the closest house to the left (when facing the front of the house), and so on, until an eligible participant is identified. This will be done until we enroll 25 women from each village who want to participate in the exposure monitoring study.

We will ask the study participants who attend the working groups to invite other village members to attend the groups. These women may be neighbors or friends of the recruited woman, members of city councils, teachers, or church members. The goal is to have 50 people participating regularly in the community working groups. All participants in the working groups will provide written informed consent.

## Methods: assignment of interventions

### Allocation concealment mechanism {16b}

To conceal study arm allocation, the program manager at Emory University will prepare eight sets of numbered, sealed opaque envelopes (and one sample set) containing intervention/control assignments. The outside of each envelope will contain a label for recording: the date of randomization; village name; group assignment (control or intervention); and the name of the person conducting the randomization. Envelope flaps will be sealed with a tamper-safe sticker. Each envelope will contain two identical folded pieces of paper, labeled either ‘intervention” or “control.” Envelopes will be stored in sequence number order in a secure location at the study office.

### Village-level randomization

#### Sequence generation {16a}

Using a randomized cluster trial design, we will randomly select one village within each matched pair to receive the community working group intervention to implement strategies to reduce the burning of plastic waste.

#### Implementation {16c}

On the day of randomization, a Guatemalan fieldworker will check out the next sequential set of envelopes, and a “sample” set of envelopes, documenting this on a Randomization Envelope Log in the field office. Randomization will occur at a community meeting with representative leaders from both villages. We will review the study goals, randomization process, and activities that will occur in both intervention and control villages during the study period. A fieldworker will show the contents of two unsealed sample envelopes, one containing a control assignment, the other an intervention assignment. They will then place the two actual randomization envelopes on a table, and each representative will choose an envelope and open it to reveal the assigned study group. One sheet of paper will be given to the village representative; the other will be placed back in the envelope. The fieldworker will then fill out all items on both randomization envelope labels. The envelopes will be returned to the study office, documented on the Randomization Envelope Log, and filed in a secure, designated location.

#### Blinding (masking) {17a, 17b}

Given the nature of the intervention, the participants and the fieldworkers will not be masked. Data analysts will be masked.

## Methods: data collection, management, and analysis

### Data collection methods {18a}

**Fig. 3:**
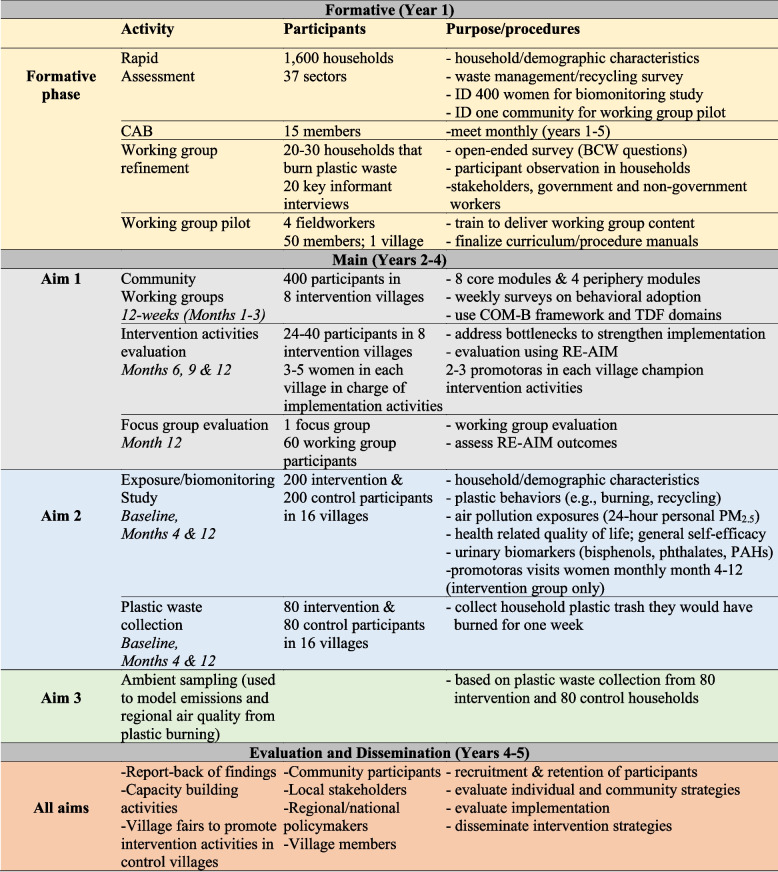
Ecolectivos summary of study activities and timeline

The Dual-PIs and co-investigators at UVG will oversee personnel training, including standardization and data entry/management.A summary of study activities and timeline is presented in Fig 3. Standard operating procedures (SOPs) have been developed in English and Spanish and are based on SOPs used during the HAPIN trial and refined during our formative research phase. The quality of air pollution and urinary biomarker monitoring will be assured during lab analysis, specimen handling, and calibration at the field headquarters, and protection of specimens in transport. Staff will use pre-printed QR-coded ID labels created by the data manager at UVG to avoid transcription errors when labeling specimens. Unique sample identification codes are the basis for sample tracking and data analysis in this study. The sample identification codes supply information regarding household and research phase and will be used in place of respondents’ names, addresses, or other identifying data to ensure confidentiality and anonymity. Sample identification codes will be printed on adhesive labels in both QR codes and human-readable format. Printed labels with the appropriate sample identification code will be affixed to sample containers. Sample identification codes will be handwritten on the physical Chain of Custody form in ink and typed into the digital Chain of Custody form in Redcap. The QR codes on the labels will also be readable by the tablet to ensure the correct questionnaires and participants are matched during data entry in the field. Training of local field personnel includes review of all SOPs, including how to minimize mistakes during biomarker and air pollution sampling in the field and in the labs. SOPs list potential errors and describe how to handle these in a uniform manner.

*Surveys:*All survey forms will be pilot tested, translated into Spanish, and back translated into English to confirm consistency with original questions to enhance reliability using procedures we have developed over the past 20 years conducting research with rural, indigenous Guatemalan communities [68, 69]. The translations and back-translations of the survey and informed consent forms are conducted by our team, comprised of Guatemalan and US-based researchers and staff, who include a Guatemalan anthropologist, data manager, and laboratory manager, eight Guatemalan field team members from local communities, and six researchers from the US, two of whom are bilingual in Spanish and English. We review survey forms in Spanish with local staff to discuss the intent of the question, and whether the wording is understandable to a limited-literacy Spanish-speaking population. Many of the team members reside in these communities and provide insightful comments that are used to modify wording without losing the intent of the question. We then pilot test the Spanish version of the questionnaire in the field, under the supervision of the Guatemalan co-investigators. Back at the office, we debrief and decide if further changes need to be made. At that point, we modify the English form to match the Spanish form.

Data for the outcome assessments will be obtained directly from participants through questionnaires, interviews, or observations. During the working groups in the intervention villages, we will administer a pre-test during the orientation session and a post-test in week 8. At each session 1–7, we will give participants two “homework” sheets which consist of a reflection sheet about what they learned in class and another sheet with questions we mapped onto the theoretical domains of the COM-B model. These questionnaires are based upon two earlier pilot studies of the intervention group. We did not conduct reliability or validity testing, beyond an assessment of face validity and comprehension of questions during the pilot testing. Participants will be asked to work with their family members to answer the questions. (See Supplementary Material). In both study arms, household visits will be scheduled over a period of two days. Fieldworkers will verbally administer questionnaires in Spanish, collecting data on sociodemographic characteristics; location and frequency of burning waste, including plastic; types and amounts of waste that are burned or recycled; other sources of household smoke, including stove type and tobacco exposures; and food/beauty care products that are known to contain PAHs, VOCs, bisphenols, and phthalates that will be measured in the biomonitoring study.

*Focus groups to assess intervention activities: *In each intervention village, we will organize meetings to assess the sustainability of interventions that were chosen over the 9-month follow-up period. These meetings will occur 3, 6, and 9 months after the community working groups have ended. The purpose of these meetings is to troubleshoot problems that are impeding the success of the chosen interventions and offer support from project staff as needed. We will also conduct focus groups to evaluate the working groups at 13 months, inviting 6–8 women (*n* = 60–80) who did not participate in the activities. The purpose of these focus groups is to evaluate the reach of the chosen interventions among those who did not participate in intervention activities. 

### Retention {18b}

Based on other longitudinal trials we have conducted in this area, we estimated that loss-to-follow-up will be 10% [70]. We will make every effort to follow the participants for the 12–13 month period of enrollment. Cash equivalent to $5–7 at the 3 biomonitoring visits will be given to women in the biomonitoring group in control and intervention villages.

*Control villages:* Participating control households will receive 40 sapling trees to plant on their land. In each of the eight control villages, village leaders will receive 3000 sapling trees for local reforestation. Community leaders will choose areas to be reforested and will organize the activities with study staff.

*Intervention villages:* We will seek permission from local village leaders to support the retention of women in the working groups. Study staff will call women to remind them about meetings of the community working groups, and will call them if they do not attend, to collect data on reasons for not attending. We will offer locally made refreshments, small gifts for attendance, transportation vouchers, and childcare. We used community-engaged research to develop intervention activities chosen by the working group participants so that the community working groups are attractive to participants. EHPs will visit participating women to assess their engagement in intervention activities. EHPs receive training materials, a cell phone with airtime, and a small monetary incentive for visiting participating households. Participants are free to withdraw from the study for any reason, at any time. Participants may be withdrawn if the study sponsor or regulatory authorities terminate the study prior to its planned end date. To reduce missing data and ensure study integrity, baseline data in those who withdraw from the study will be analyzed to assess differences at baseline with those who participated fully. We will analyze all data that has been collected, including air pollution and urinary biomarkers, on those who withdrew from the study.

### Data management {19}

Data management at the field site will be overseen by the project manager and the data manager. Enrolled participants will be assigned a unique participant ID number that will be linked to all of their data. Survey forms, data dictionaries, and databases will be created in REDCap (Research Electronic Data Capture). Data will be entered directly into REDCap on password-protected tablets by trained fieldworkers. Reports generated by the data manager will provide summaries such as the number of visits completed, exceptions to the protocol (missed visits, missing forms), and dates of expected visits. Data collected on paper study forms (e.g., homework collected during the community working groups) will be entered into REDCap and then kept on file at the participating site. A subset of 10% of these forms will be double-entered and reviewed for accuracy by the supervisor. If inaccuracies are found, the supervisor will retrain staff. Data consistency checks will be implemented. Data query reports will be generated by the data manager and sent to the field supervisors to clarify missing or erroneous data.

### Confidentiality {27}

All data will be stored securely and confidentially. Participant identifiers will be stripped of protected health information and will be assigned in a way to improve tracking and monitoring of study activities. Access to data will be restricted to authorized study staff only. Participants will be informed of their rights concerning data privacy and the use of their information during the consent process.

### Statistical methods {20a}

Pre- and post-test instruments, and surveys administered during each of the working group sessions, are semi-structured (See Additional File 3 in Supplementary Information). Close-ended questions will be analyzed using descriptive statistics, and textual responses from open-ended questions will be coded into themes. Key informant interviews will be audio-recorded and transcribed. Transcripts, notes from participant observations, and ethnographic field notes will be coded and analyzed. We will use thematic analysis, which is flexible and theoretical and can be applied across a range of qualitative methodologies. Categories (i.e., themes or variables) and their properties (sub-categories) will be tested deductively [71] based on the constructs of the BCW. New themes will be identified and coded using an inductive thematic approach.

Mixed methodologies offer causal explanations grounded in different kinds of empirical data [72]. Informant-driven qualitative findings, such as responses from focus groups post-intervention and semi-structured instruments administered during the working groups, will be the following: (1) assessed against demographic characteristics, such as education level, asset ownership, and age, of working group participants; (2) linked to the reach, effectiveness, adoption, and sustainability of intervention strategies (Aim 1); and (3) compared to quantitative findings from air pollution exposures and biomarkers (Aim 2). Thus, mixing methods may contextualize both the depth (qualitative) and breadth (quantitative) of patterns of plastic burning and changes related to the intervention strategies [73].

#### Aim 1: Evaluation of intervention activities using implementation research approaches

Our evaluation of intervention implementation involves components of the COM-B model/TDF and the RE-AIM framework. Measures related to the COM-B model (described previously) that are included in the study data collection are outlined in Table 2. The evaluation of the intervention includes three main approaches: (1) we will measure individual-level changes in plastic waste use and burning behaviors among all working group participants over the age of 15 years using data collected during the working group meetings, by the EHPs at their monthly home visits, and from our field notes (intervention arm only); (2) we will evaluate the community-level changes in these same plastic-use and burning-related measures among the 400 participants involved in the biomonitoring study, also with survey methodology (both intervention and control arm); and (3) we will measure changes in urinary biomarkers and personal exposures to air pollution in 400 women in the biomonitoring study (both intervention and control arm). Together, these approaches will inform us about community-level impact among women who attend and do not attend the working groups.

We will also use the RE-AIM framework [34, 35], primarily focusing on overall reach as well as on effectiveness, as measured by changes in individual-level household behaviors (women who participate in working groups in intervention villages). Specifically, *Reach* of the intervention will be assessed by participants’ level of engagement in working groups (attendance frequency, duration, and completion). *Adoption* will be measured by the number and types of behavior changes based on working group strategies. This will include the number of participants in community working groups who report reducing, reusing, or recycling plastic and reduced plastic burning, as reported by survey data. Additionally, we will measure village-level reach measured by the level of involvement in village-level activities that arise from working group survey data [74]. *Effectiveness* will be measured by the level of behaviors (high/low levels of reducing, reusing, or recycling plastic) reported by participants and its effect on perceived health and well-being outcomes, including collective efficacy, general self-efficacy, health-related quality of life, and reductions of urinary biomarkers of exposure. Using monthly data collected at the household level by EHPs, we will assess *Maintenance* by the number and proportion of intervention participants (25 biomonitoring women and working group attendees) who remained engaged in one or more plastic waste reducing behaviors (e.g., participation in a community clean-up program) to reduce consumption, increase recycling, and who report no longer burning plastic. We will assess which program components are essential for success [75]. Results for Aim 1 will be analyzed and disseminated by May 2027.

#### Aim 2: Longitudinal effect of behavioral intervention on air pollution and urinary biomarkers

*Urinary biomarkers and air pollution:* Urinary biomarker and air pollution concentrations are typically right-skewed and will be described as the geometric mean (95% confidence interval). We will estimate variance components to assess intraclass correlations among repeated measures within subject and village.

*Analysis methods:* We will log transform our data and use linear mixed effects models. Multi-level mixed effects models (MLM) with random (individual- and village-level) and fixed (treatment group, study period, and group-by-period interaction) effects will be employed to test for trajectories of change for intervention group versus control group, focusing on the group-by-time interaction effect. If there are significant differences between the 2 groups at baseline (e.g., difference in ambient air pollution), these will be controlled by design and estimated by the main effect for the study group. MLM longitudinal models evaluate the changes across the three time points, and the interaction of group and each separate follow-up time point will allow planned post hoc estimates of both the immediate intervention effects (4 months) and the longer term sustained effects (12 months). To adjust for potential confounding, as well as to increase efficiency by reducing residual variance in urinary biomarker concentrations, we will adjust for indicators of dietary intake (ingestion route) and cosmetic product use (dermal route) plastic contaminants. We will use the Stepdown-minP procedure to calculate *p*-values that take into account multiple comparisons and the correlation expected among multiple measures of air and urine concentrations [76]. All model assumptions will be tested with standard diagnostic tests and influence statistics used to test the distributions of the residuals. MLM utilize all available data for all participants at each time point.

### Methods for any additional analyses (e.g., subgroup and adjusted analyses) {20b}

In addition to the overall effect, we will explore heterogeneity in effects between communities using a meta-analysis approach. This will be done by estimating the intervention effect within each of the matched-pair communities, aggregating the effect sizes across matched pairs to derive an overall intervention effect, and testing for heterogeneity in the effect of the working group interventions on exposure to products of plastic combustion across matched pairs of communities [77]. This examination of variation in the intervention effect across matched pairs has the potential to answer important questions about why the working groups may be more successful in one set of matched pairs compared to other matched pairs. For example, the heterogeneity may be partly explained by fidelity to the working group protocols, the level of community participation, or the types of interventions that are implemented. Moreover, as a secondary analysis, we will explore the impact of the interventions not only on the mean of the exposure distribution but also on its shape. This will be performed using quantile regression to compare distributions between and within groups [78]. This will allow us to compare the effects of the interventions at various locations of the exposure distribution.

### Methods to handle protocol non-adherence and statistical methods to handle missing data {20c}

We will conduct intention-to-treat analysis. From our previous experience conducting longitudinal studies in rural Guatemala, we expect about 85% completeness accounting for loss to follow-up and intermittent missingness due to logistic, communication, and technological failures [79]. We will use the doubly robust estimation method to address bias due to missing data [80]. First, we will explore models to predict missing exposures, including predictors such as baseline characteristics, previous exposure measures, and reported and observed behaviors related to exposure sources. If this model has predictive validity, we will use it in a multiple imputation sensitivity analysis. Our second approach will be to build a model that explains missingness by modelling the probability of missingness conditional on treatment assignment and baseline covariates, as in inverse probability weighting. Finally, if both models are found to be predictive, we will apply the doubly robust estimation for targeted inference, which relies on parameters from either of these models being estimated consistently [81]. R software will be used [82]. Results for Aim 2 will be analyzed and disseminated by May 2027.

#### Aim 3: Model regional air pollution scenarios from plastic waste burning

We will collect all household waste that is produced by 5 participating families in each village and estimate the masses of plastic waste to quantify emissions of 62 chemical species, as described in Bardales et al. [44]. The emissions estimates for each of the species will have uncertainties associated with them by conducting 1 million Monte Carlo samplings using the mean and standard deviation of mass estimates and emission factors (EFs). Using the most recent 2018 Guatemalan census data and the amount of garbage produced [7], as well as the percentage of plastic waste from the recent World Bank database [83], we will create emissions estimates, again using the 1 million Monte Carlo samplings. Due to data availability for Guatemala, in addition to Jalapa, we have city-level emissions estimates for Guatemala City, Antigua, and Jutiapa. For other parts of Guatemala, we will use country-level estimates. These emissions estimates will then be spatially distributed across Guatemala, following the garbage burning emissions estimates distribution in our pilot study [44]. This activity will occur three times at baseline (the start of the study), 4–6 months, and 12–13 months from the baseline visit.

For forecasting future emissions from plastic burning, we will create several emissions scenarios for the year 2030. Based on differences in plastic waste collected in households over time, we will estimate the mitigation potential for different chemical species of interest from reduced plastic waste in Jalapa, Guatemala, following the similar methodology used in Saikawa et al. [84]. We will then use the “online” Weather Research and Forecasting (WRF) model coupled with Chemistry (WRF-Chem) [85, 86] to simulate the regional air quality over Guatemala and Central America to assess the impacts of different plastic emissions on air quality at the local and regional levels. The model domain will cover most of the Central America region with two nested domains: one for Guatemala and the innermost domain for Jalapa. For each scenario, we will use WRF-Chem to model air quality for several months of the year to understand seasonality. For each simulation, the model will be spun up for 14 days to allow the model to ventilate the regional domain, and this period will not be included in the analysis. For our baseline simulation, we will evaluate our WRF-Chem model results by comparing simulated pollutant concentrations with observational data available in Guatemala. By modifying emissions from the domestic sector and using the most optimal scenario where we assume there is no more plastic burning to the business-as-usual scenario where we assume that plastic burning continues and potentially worsens in the future, we will be able to quantify the percentage reduction in various pollutant concentrations among scenarios. Results for Aim 3 will be analyzed and disseminated by May 2027.

## Methods: oversight and monitoring

### Composition of the coordinating center and trial steering committee {5d}

The coordinating center is based at Emory University, Atlanta, GA, where the Dual PIs oversee the overall components of research activities, assisted by the study manager. They work in close collaboration with the local principal investigator and co-investigators at UVG, the implementing partner in Jalapa, Guatemala. Monthly teleconferencing meetings are held with all principal and co-investigators who form the steering committee. The Dual PIs visit the field site monthly to quarterly to supervise project activities, assure compliance with study procedures, review data management, and address issues as they arise. The first author of this publication meets weekly via teleconference with the field team and the data manager in Guatemala. The local PI, employed by UVG, visits the field site weekly and oversees the laboratory where the urine samples are collected, transported, stored, and shipped to Emory. The co-investigator at the University of Georgia provides overall supervision, quality control, and data of the UVG air pollution lab where filters are weighed. The local field team has an air pollution supervisor and an intervention supervisor, each of whom oversees the activities of 3–4 field staff. The local field team, all of whom are Guatemalan, reside permanently in Jalapa.

### Composition of the data monitoring committee, its role and reporting structure {21a}

This study does not have an independent data monitoring committee. The rationale for this is based on the low potential risks from data collection procedures. Most data are obtained by interviewer-administered surveys, observations, or non-invasive procedures and pose no risk of physical harm.

### Interim analysis {21b}

Because we do not have a health endpoint that we are monitoring, we do not have a plan for interim analyses and formal stopping rules.

### Adverse event reporting and harms {22}

Reporting of adverse events will follow requirements established by the Institutional Review Boards (IRBs) at Emory and UVG. A standard operating procedure has been developed, and the field team has been trained on the reporting of adverse events and serious adverse events. Data will be collected by the field project manager at the research site on case report forms and then reported to the Dual-PIs at UVG and Emory University. Serious adverse events will be reported to the Institutional Review Boards (IRBs) at Emory and UVG within 24 hours.

### Auditing {23}

Other than the annual audits required by our sponsor, we do not have an independent auditor assigned to the study. We will internally audit the trial through the following: (1) data reports generated by the data manager and communicated to the Dual PIs; (2) the built-in audit trail in the REDCap database which shows changes made to the project databases; and (3) data quality control checks for air pollution, urinary biomarkers, and survey data; and (4) auditing the consents for consistency.

### Plans for communicating important protocol amendments to relevant parties {25}

Changes to the protocol will be communicated to the ethics boards at Emory University and UVG (the implementing partner), and the protocol will be amended. Any changes to the protocol must be submitted to and approved by Emory and UVG prior to implementation. The program manager communicates all changes to the steering committee and is in charge of maintaining all protocols in a shared folder on Emory’s OneDrive for investigators to access as needed. Protocol violations are reported using a case report form to Emory, the IRB, and regulatory authorities as applicable. We will update the protocol on the clinical trial registry ClinicalTrials.gov with the assistance of the Clinical Trials Compliance Officer in the Office for Clinical Research (OCR) at Emory University. The sponsor will be notified of any major changes to the protocol.

### Provisions for post-trial care {30}

Following the completion of the 12-month intervention, intervention group participants will continue to have access to materials provided to them during the community working groups, to ensure that they can continue with their intervention activities (e.g., tools for community clean-ups; composting bins). We will host “intervention fairs” in control villages where they will learn about working group activities and will be provided with recycling and community clean-up support after the trial. Financial compensation provided to both groups during the study, in the form of food, travel vouchers, and small compensations for participating in the biomonitoring activities, will end. There are no provisions for proposed ancillary post-trial care for this study beyond usual care.

## Ethics and dissemination

### Research ethics approvals {24}

The protocol, informed consents, recruitment, case report forms, and educational materials were reviewed and approved by Emory University and UVG’s ethical committees. This study has approval from the Emory University Institutional Review Board (#00002412) and the Research Ethics Committee of the Center for Health Studies of the Universidad del Valle de Guatemala (approval #245-05-2021). Modifications to the protocol are reviewed and approved by both ethical committees. Reliance agreements are in place with the University of California San Francisco and the University of Georgia to rely on Emory’s IRB approval.

### Protocol amendments {25}

When protocol, consent, and assent modifications are made, they are submitted to the IRB/Ethics Committees, and modifications will not be implemented until official approval is granted. The principal investigator will submit revisions to the ClinicalTrials.gov registry. If modifications affect the conduct of the trial, the field team will be informed of any change in procedures. If consent forms are revised, current participants will be re-consented.

Written informed consent or assent for all procedures, including ancillary studies, will be obtained by a trained local fieldworker in Spanish. Steps will be taken to ensure participants’ comprehension at an elementary-school level. Written assent will be provided for participants between 15 and < 18 years of age, and the designated legal guardian will provide consent for their child to participate in the study. The study will employ standard methods for protecting the confidentiality and privacy of participants.

### Dissemination policy {31a and b}

Results will be shared with participants and participating communities at dissemination meetings conducted in the control communities in Year 5. We will discuss successful practices intervention communities developed to reduce plastic waste burning as a result of the workshop activities (Aim 1). We will invite local policymakers to attend meetings to learn about the results. We will evaluate changes in exposures to air pollutants and urinary biomarkers in women measured at 4 and 12 months after baseline (Aim 2). Aggregated results will be presented at dissemination meetings in Year 5. We will also present air quality modeling results (Aim 3) at the dissemination meetings. We will present findings in such a way that they are understandable to lay audiences. Results will be written by co-investigators, with authorship based on the International Committee of Medical Journal Editors (ICMJE) guidelines [87] and presented in open access, peer-reviewed scientific journals and at scientific conferences and will involve collaborators as appropriate. To comply with NIH Public Access Policy, peer-reviewed accepted publications will be uploaded to the National Library of Medicine’s PubMed Central by the study researchers or the journal.

### Plans to give access to the protocol, participant-level data and statistical code {29, 31c}

Data will be de-identified before it is shared. The IRB-approved protocol and informed consent documents will include language describing the data management and sharing plan, explaining the motivation for sharing, and ensuring that personal identifying information will be removed prior to sharing. Dataset(s) resulting from this research will be shared via the generalist repository Dryad, which provides metadata, persistent identifiers (i.e., DOIs), and long-term access. Dryad is the institutional data repository supported by the University of California, and all data is shared under a creative commons waiver (CC0), which makes the dataset(s) publicly available. Data will be made available as soon as possible or at the time of associated publication. A DOI will be obtained for both final analytic code and data, which will be available through DRYAD and GitHub. Relevant resources, such as code used for data processing and analyses, will be made publicly available through GitHub, a secure web-based platform that hosts source codes, documentation, and project-related web content for research projects.

## Discussion

Plastic waste disposal and the open burning of plastic in household fires are major problems in waste management globally [88], posing risks to human health. To mitigate this risk, local, national, and international policies need strengthening. While there are global efforts to introduce clean cookstoves, these programs do not address plastic that is commonly burned in household cooking fires [89]. Aside from our own research to estimate plastic waste burning [84] and our pilot exposure assessment study for this proposal [37], we found only one intervention study that used a community approach to reduce open burning of plastic waste [90]. Furthermore, there are no global emissions estimates from plastic waste burning in developing countries where a majority of households burn waste as the primary means of disposal [7].

To the best of our knowledge, this is the first study using an implementation-effectiveness trial design to systematically refine and implement a behavioral intervention to address plastic waste burning, as well as conduct a detailed personal exposure assessment. We reviewed 6974 studies and identified 43 studies that used interventions to address the management of plastic waste in low- and middle-income countries. These studies represented 27 unique countries across Asia, Africa, and Latin America. All studies could be broadly categorized into broad intervention categories, including educational and awareness campaigns, infrastructure development, regulatory policy, economic incentives, behavioral nudges, and clean-up campaigns. One study used a trial design to evaluate whether social competition between neighborhoods effectively motivated community leaders and residents to reduce informal waste burning in urban areas of Uganda [90]. However, none of the studies specifically addressed household-level plastic waste burning using a theory-informed behavioral intervention, as we plan to do.

We will use a village-level cluster randomized design to evaluate whether community/individual behavioral interventions reduce the quantity of plastic waste combustion. Using a randomized design will allow us to evaluate the causal effects of these interventions on women’s personal exposures and urinary concentrations of contaminants produced by plastic combustion. We will refine, implement, and evaluate the reach, effectiveness, adoption, and maintenance of intervention strategies from village-level working groups using the RE-AIM framework. This study will provide estimates of how much plastic waste is burned in homes in Jalapa, Guatemala, and how this is manifested in air pollution exposures and urine concentrations of chemicals found in women exposed to burning plastic. We will create several emissions scenarios, based on our estimates and census data, to assess the impact of the intervention to reduce plastic waste burning on local and regional air quality.

The long-term goal of the Ecolectivos study is to build sustainable local and national strategies based on findings from community working groups. We will work in partnership with local community members to address an issue of concern to them, the accumulation of plastic waste in their households and communities. However, there are several limitations to the implementation of our proposed intervention activities at the local level. We anticipate that the biggest barriers will be infrastructural and economic constraints [91], as well as the challenges of changing behaviors in a resource-limited environment. The 12-week working group intervention is labor-intensive and bears financial costs (e.g., training educators, providing materials) and might not be scalable in the current form. However, the EHPs may be an important motivating force in their villages, since they will be trained to provide educational messages and support alternative activities to burning plastic waste. Our EHPs may feel empowered to share their knowledge with their networks to facilitate social norm change, as has been seen in other studies [91, 92]. Further, intervention activities, like community clean-ups and recycling programs, instituted by women participating over a period of 12 months, may be more likely to continue, given the length of involvement of the women working on these projects independently. The long-term sustainability in intervention communities and the potential adoption in other communities may be limited in scope if behaviors and intervention activities are abandoned.

Dissemination of our findings to local governments may allow for the development of similar activities that could be used more broadly. But without local governmental support and policies to guarantee efforts to reduce and remove waste and to promote recycling [93], communities may not be able to reduce plastic waste burning. Rural indigenous populations are isolated, and community needs are not consistently represented by governmental policies and actions. However, Guatemalan indigenous movements, including in the Xinca communities of Santa Maria Xalapán, have resisted mining projects and hydroelectric dams, realizing the damage these exert on local ecosystems [28]. This context may challenge or complement the adoption and spread of alternatives to plastic waste burning in the region of Jalapa.

Findings from our study may provide evidence that regional and national governmental agencies need to justify services, like increased sanitation services in rural areas, and policies, like regional plastic bans [94] and national plastic action plans [95] that are currently in place in Guatemala. By modeling contributions to air pollution made by plastic waste burning in one region, we may provide an approach for future intervention programs that combine behavioral intervention strategies with exposure assessment for policy-relevant solutions. Drawing from the perspectives of human rights and ecological justice, our research initiative aims to mobilize local communities towards sustainable development.

### Trial status

The protocol was first submitted on ClinicalTrials.gov on October 20, 2021. The first IRB-approved protocol was dated April 27, 2021. This manuscript refers to protocol version 1.8, dated May 19, 2025. The protocol revision history is summarized after the title page of the protocol. The formative phase was conducted between August 18, 2021 and May 31, 2022. Participant recruitment into the trial started on October 25, 2022 and ended on November 14, 2024. Completion of primary endpoint data analysis will be May 31, 2026. Due to extensive review processes, there was a delay in final publication. This manuscript was submitted to another journal before recruitment ended but was not accepted. It was then revised to be submitted to this journal, and the revision was not completed until after recruitment ended.

## Supplementary Information

Additional file 1: Protocol: Combustion of plastic waste and human health effects in Guatemala.

Additional file 2: SPIRIT 2013 checklist: Recommended items to address in a clinical trial protocol and related documents.

Additional file 3. Materials for community working groups: COM-B surveys, homework sheets, pre- and post-tests. English and Spanish.

## Data Availability

All requests for data use will be approved by the Dual-PIs. Public access to a de-identified dataset and statistical code will be published and freely accessible in a data repository using a digital object identifier (DOI) for other researchers’ use. The Spanish-language working group manual (and the English-language version) and other modular materials (PowerPoints, handouts, and other didactic materials) are available on the Ecolectivos website [53].

## Abbreviations

BC: Black carbon
CAB: Community advisory board
COCODE: Community Development Council
COM-B: Capability-Opportunity-Motivation-Behavior model
BCW: Behavior Change Wheel
cRCT: Cluster randomized controlled trial
EHPs: Environmental Health Promotors
HAPIN: Household Air Pollution Intervention (HAPIN) trial
PAHs: Polycyclic aromatic hydrocarbons
PM_2.5_: Fine particulate matter, less than 2.5 microns in diameter
PTFE: Polytetrafluoroethylene
TDF: Theoretical Domains Framework
TPB: 1,3,5-Triphenylbenzene
UVG: Universidad del Valle de Guatemala
VOCs: Volatile organic compounds
