# Supplementary material for: A village-level cluster randomized controlled implementation trial to measure the effectiveness of a behavioral intervention aiming to reduce women’s exposures to household plastic waste burning in rural Guatemala: study protocol for the Ecolectivos trial
Source: Trials. 2025 Dec 13;27:49. doi: 10.1186/s13063-025-09338-z (PMC12817428; doi:10.1186/s13063-025-09338-z)
Supplement: Supplementary file 1 — Additional file 1: Protocol: Combustion of plastic waste and human health effects in Guatemala. [file 13063_2025_9338_MOESM1_ESM.pdf]

PROTOCOL TITLE: Combustion of plastic waste and human health effects in Guatemala

EMORY UNIVERSITY COLLABORATORS

Principal Investigator (contact): Lisa M. Thompson, PhD, RN, FNP, Professor, Nell Hodgson Woodruff School of Nursing, Emory University, Atlanta, GA lisa.thompson@emory.edu

Principal Investigator: Eri Saikawa, PhD, Professor, Environmental Sciences, Emory University, Atlanta, GA eri.saikawa@emory.edu

Co-Investigator: Dana Boyd Barr, PhD, Research Professor, Gangarosa Department of Environmental Health, Rollins School of Public Health, Emory University, Atlanta, GA dbbarr@emory.edu

Co-Investigator: Melinda Higgins, PhD, Research Professor/Senior Biostatistician, Nell Hodgson Woodruff School of Nursing, Emory University, Atlanta, GA mkhiggi@emory.edu

EXTERNAL (NON-EMORY) COLLABORATORS

Co-Investigator: Margaret Handley, PhD, MPH, Professor, Department of Epidemiology and Biostatistics, Division of Preventive Medicine and Public Health; University of California, San Francisco Margaret.handley@ucsf.edu

Co-Investigator: John P. McCracken, ScD, MPH, Professor, Global Health Institute, Epidemiology & Biostatistics, University of Georgia, Athens, GA john.mccracken@emory.edu

Co-Investigator: Maria Renee Lopez, Investigator at Center for Health Studies, Universidad del Valle de Guatemala, Guatemala City, Guatemala mrlopez@uvg.edu.gt *UVG will review protocol*

Co-Investigator: Dr. Mayari Hengstermann, PhD, Medical Anthropologist, Center for Health Studies, Universidad del Valle de Guatemala, Guatemala City, Guatemala mhengstermann@uvg.edu.gt

PRINCIPAL INVESTIGATOR:

Lisa M. Thompson, PhD, RN, FNP, Professor
Nell Hodgson Woodruff School of Nursing, Emory University, Atlanta, GA
Phone 510-965-7779
lisa.thompson@emory.edu

VERSION: 1.8

FUNDING SOURCE: NIH: National Institute of Environmental Health Sciences
Award numbers: 1R01ES032009; 3R01ES032009-03S1; P2CES033430

REVISION HISTORY

Revision #	Version Date	Summary of Changes
1.1	June 21, 2021	Modifications based on feedback from Universidad del Valle de Guatemala's Ethical Review Board
1.2	December 14, 2021	Modifications to local PI and study endpoints based on proposal and clinicaltrial.gov registration.
1.3	April 29, 2022	Modified exposure monitoring in formative phase; modified numbers of participants in formative and main trial; Modified baseline assessment and formative phase consents
1.4	July 15, 2022	Modified formative phase: 1) pilot study of exposure monitoring to include a 2nd pilot measurement visit and urine collection, and 2) added a rapid ethnographic assessment, or community diagnosis; 3) Modifications to study endpoints; 4) Modification to main study to add second baseline assessment in 25 households in 16 villages. Modified formative consents.
1.5	March 31, 2023	• Moved rapid ethnographic assessment, or community diagnosis from formative phase to Main Trial<br>• Changed 'plastic' burning to 'trash' burning. Added a third timepoint for trash-burning (12-13 months).<br>• Added control village compensation.<br>• Added intervention village compensation.<br>• Added up to 3 environmental health community workers per intervention village.<br>• Added ambient exposure monitoring<br>• Added kitchen and outdoor fire exposure monitoring
1.6	February 1, 2024	• Added silicone wristbands for personal environmental chemical exposure monitoring.<br>• Modified timing and content of focus groups to evaluate working groups, and individual interviews and follow-up meetings to evaluate intervention activities.
1.7	October 22, 2024	• Removed description of Community Advisory Board.<br>• Re-categorized outcomes.<br>• Ensured all study activities were consistent across all corresponding protocol sections.<br>• Added study team evaluation activities.

1.8	May 19, 2025	• Added 2 funding sources to front page of protocol (already included in Funding Sources page of eIRB)<br>• Made minor edits to primary outcome definitions (for clarity only)<br>• Revised secondary and tertiary outcome definitions based on input from the Clinical Trials Compliance and ClinicalTrials.gov Team in the Emory Office for Clinical Research (OCR)<br>• Revised the sample size power and effect size calculations for Aim 1.<br>• Updated timeline

Table of Contents

1. Study Summary 5
2. Objectives 8
3. Background 9
4. Study Primary Endpoints 10
5. Study Secondary Endpoint 10
6. Study Tertiary Endpoint(s) 12
7. Study Intervention/Design 13
8. Procedures Involved 15
9. Data Specimen Banking 25
10. Sharing of Results with Participants 25
11. Study Timeline 25
12. Inclusion and Exclusion Criteria 28
13. Population 30
14. Vulnerable population 30
15. Local Number of Participants 30
16. Recruitment Methods 30
17. Withdrawal of Participants 32
18. Risk to Participants 32
19. Potential Benefits to Participants 33
20. Compensation to Participants 33
21. Data Analysis, Management and Confidentiality 34
22. Provisions to Monitor the Data to Ensure the Safety of Participants 41
23. Provisions to Protect the Privacy Interest of Participants 44
24. Economic Burden to Participants 46
25. Informed Consent 46
26. Setting 46
27. Resources Available 47
28. References 49

1. Study Summary

**Study Title**	Combustion of plastic waste and human health effects in Guatemala
**Study Design**	Randomized cluster trial
**Primary Objectives**	1. Using community-based dynamic working groups, implement and evaluate intervention strategies that reduce plastic waste burning, targeting barriers and enablers identified within the capability, opportunity, and motivation domains, for key behaviors (guided by Michie's Behavior Change Wheel framework), focusing on assessment of implementation fidelity, reach and potential for scale-up (guided by Glasgow's RE-AIM framework).<br>2. Compare urinary biomarkers of exposure to plastic combustion (bisphenols, phthalates, polycyclic aromatic hydrocarbons and volatile organic compounds) and personal airborne fine particulate matter ($PM_{2.5}$) and black carbon (BC) in reproductive age women. ***Hypothesis***: Biomarkers and exposures will decrease over time in 200 women from 8 intervention villages compared to 200 women from 8 control villages at 4 and 12 months.<br>3. Using filter-based antimony (Sb) and 1,3,5-Triphenylbenzene (TPB) as tracers of plastic burning and collecting household plastic waste, apportion $PM_{2.5}$ and quantify emissions estimates of air pollutants from plastic incineration and assess effects of potential emissions reduction on air quality with a chemical transport model.
**Secondary Objective(s)**	1. To provide robust evidence of sustainable local strategies based on findings from community working groups to reduce air pollution from plastic trash burning.<br>2. To develop an approach for policy-relevant solutions that combine evidence from behavioral intervention evaluation, exposure assessment, and atmospheric emissions.
**Research Intervention(s)/Interactions**	During the **Formative phase year 1**, we will conduct a baseline assessment to identify villages and women of reproductive age for the Main Trial. We will identify 400 women of reproductive age (25 from each village) who report burning plastic trash as a primary form of waste disposal at the baseline assessment. We will pilot the community working group intervention in one village with 100 participants and pilot personal, kitchen and outdoor area exposure assessments in 12 households as a practice run for the Main Trial in Years 2-4.<br><br>For the **Main trial** (Years 2-5), we will conduct a rapid ethnographic assessment in 16 villages. We will enroll 400 women of reproductive age in 16 communities and collect baseline information on them. We will ask the 200 women in the intervention villages to participate in the working groups. We will invite and consent between 25 and 50 additional community members to participate in 12-week working groups and to

	participate in activities related to these working groups. Up to 3 environmental health community workers (*promotoras*) in each intervention village will engage with participants to encourage their participation in the working groups. Each intervention community will commit to alternatives to burning plastic and drive initiatives they can achieve over the next 9 months. Our field team will work with the *promotoras* in the intervention communities over the 9-month period to address bottlenecks and problems as they arise. We will conduct biomonitoring (urine specimens) and air monitoring on 400 women in both the intervention and control villages. We will quantify village-level emissions estimates of air pollutants from plastic incineration using tracers of plastic burning and collection of household waste in the 16 villages. We will conduct kitchen and outdoor fire air pollution sampling in 80 households. In addition, we will install 5 ambient monitors in the local area (community centers, schools) distributed spatially among villages.<br><br>In **Year 5**, we will evaluate our program and disseminate our results to the participating communities, to regional and national policy makers to achieve our secondary objectives listed above.
**Study Population**	The study population will be drawn from households in villages who take part in the baseline assessment in the rural Xalapán region of Jalapa, Guatemala, where the pilot study for this proposal was conducted in 2019-2021.
**Sample Size**	**Formative phase**<br>-1,630 women who report that they are the primary cook in the household to conduct baseline assessment and participant observations<br>-12 households participating in personal and kitchen monitoring where plastic trash is burned<br>-Up to 50 participants for open-ended survey<br>-10-15 key informant (stakeholder) interviews<br>-100 participants for pilot working group intervention<br><br>**Main trial**<br>-Up to 160 participants for rapid ethnographic assessment (community diagnosis) in 16 villages<br>-400 women of reproductive age for biomonitoring study (25 women from each of the 8 intervention villages and 25 from each of the 8 control villages)<br>-80 women (40 in each study group) for kitchen and outdoor fire exposure monitoring<br>-80 women (40 in each study group) for 1 week of household waste collection<br>-Up to 600 participants in working groups (of which a subset will be women of reproductive age who are in the biomonitoring study)

	-16-20 (2 – 3 participants per intervention village) *promotoras*<br>-Up to 80 *promotoras* and participants in the working groups who participated in one or more intervention activities will be included in follow-up evaluations of progress with intervention activities.<br>-Up to 4 study team members who work in the intervention villages will be interviewed to evaluate promotoras and community intervention representatives<br>-100 women in 4 villages to wear silicone wristbands for personal environmental chemical exposure monitoring.
**Study Duration for individual participants**	**Year 1: Formative**<br>-Baseline assessment (20-30 minutes one time)<br>-Participant observation (2-4 hours observation)<br>-Air monitoring (24-hours air monitoring)<br>-Open-ended survey (45-60 minutes one time)<br>-Home observations (2-4 hours one time)<br>-Pilot working group intervention (2 hours/week over 12-weeks)<br><br>**Year 2-4: Main Study**<br>-Interview for rapid ethnographic assessment (45-60 minutes one time)<br>-Follow-up assessment/recruitment into biomonitoring study (45-60 minutes one time)<br>-Working group participants (12 months total, 2 hours/week over 12 weeks + 1 hour/week for 9 months as necessary)<br>- Biomonitoring participants - study visits (interviews, air pollution exposure monitoring, and urinary biomarker collection), 3 times: baseline—before start of working group sessions; 4-5 months—at end of working group sessions; and 12-13 months from baseline (45 minutes on day 1, 30 minutes on day 2)<br>-Kitchen and outdoor fire exposure monitoring (1-2 hours, 3 times)<br>-Household waste collection for 1 week (3 hours a week, 3 times)<br>-Intervention group *promotoras* - weekly phone calls to participants (1-2 hours/week over 9 months), monthly household visits to participants (10 hours/month over 9 months), weekly communication with the research team (15 minutes/week over 9 months), monthly meetings with participants engaging in the intervention activities (2 hours/month over 9 months), provide logistical support and track activities and attendance of participants at the working group meetings (1-2 hours/week over 8 weeks), attend meetings with community leaders to scale up intervention (5 hours total), participate in activities related to the evaluation of the intervention (2 – 4 hours, 3 times), assist in development and delivery of 'intervention fairs' in 8 control villages (4-6 hours total).<br>-Focus groups with working group participants (1-1 1/2 hours, 1 timepoint).

	-Interviews and follow-up meetings with intervention group participants and *promotoras* to evaluate intervention activities (1-1 1/2 hours, at 3 timepoints).<br>-Study team evaluation of the progress of *promotoras* and community intervention representatives (1-1 ½ hours, 3 times)<br>-Personal exposure monitoring via silicone wristbands (20 minutes on day 1, 1 week inactive (wearing of wristband), and 30 minutes on day 8, 2 times [baseline and 4-month visit]).<br><br>**Year 5: Evaluation and Dissemination**<br>- Disseminate results in community meetings (2 hours, once)
**Study Specific Abbreviations/ Definitions**	BC: Black carbon<br>BCW: Behavior Change Wheel Framework<br>BPA: Bisphenol A<br>HRQoL: Health-related Quality of Life instrument<br>PAHs: Polycyclic Aromatic Hydrocarbons<br>$PM_{2.5}$: Particulate Matter, <2.5 microns in aerodynamic diameter<br>PTFE: Polytetrafluoroethylene<br>BA: Baseline Assessment<br>RE-AIM: reach, effectiveness, adoption, implementation fidelity and maintenance framework<br>Sb: Antimony, tracer of plastic incineration in $PM_{2.5}$<br>TPB: 1,3,5-Triphenylbenzene, tracer of plastic incineration in $PM_{2.5}$<br>VOCs: Volatile Organic Compounds
**Funding Source (if any)**	NIEHS: National Institute of Environmental Health Sciences
**ClinicalTrials.gov Identifier**	NCT05130632

2. Objectives

The **objective** of this study is to evaluate the uptake, environmental health impact, and sustainability of intervention strategies to reduce use, recycle, and repurpose plastic aimed at reducing household-level plastic burning in communities.

Our specific aims are:

1. Using community working groups, implement and evaluate intervention strategies that address plastic waste burning, targeting barriers and enablers identified within the capability, opportunity, and motivation domains, for key behaviors (guided by Michie's Behavior Change Wheel framework), focusing on assessment of reach, effectiveness, adoption, maintenance and potential for scale-up of the intervention activities (guided by Glasgow's RE-AIM framework).

2. Evaluate the impact of behavioral interventions on urinary biomarkers of exposure to plastic combustion (bisphenols, phthalates, polycyclic aromatic hydrocarbons and volatile organic compounds) and personal airborne fine particulate matter ($PM_{2.5}$) and black carbon (BC) in reproductive age women. *__Hypothesis__*: Biomarkers and exposures will decrease over time in women from intervention villages compared to women from control villages at 4 and 12-13 months.
3. Using filter-based antimony (Sb) and 1,3,5-Triphenylbenzene (TPB) as tracers of plastic burning and collecting household plastic waste, apportion $PM_{2.5}$ and quantify emissions estimates of air pollutants from plastic incineration and assess effects of potential emissions reduction from the working group intervention on air quality with a chemical transport model.

Secondary Aims:

1. To provide robust evidence of sustainable local strategies based on findings from community working groups to reduce air pollution from plastic trash burning.
2. To develop an approach for future intervention programs that combine behavioral intervention evaluation with exposure assessment for policy-relevant solutions.

3. Background

Household air pollution from solid fuel combustion (e.g. wood) is a major environmental risk factor in low- and middle-income countries, accounting for an estimated 2.6 million deaths annually (World Health Organization, 2016). The contribution of plastic waste incineration in household fires has not been quantified. While there are global efforts to introduce clean cookstoves as a replacement for solid fuels, these programs do not address reducing the mounting plastic that is burned in household fires (Clasen et al., 2020; Cordes, 2011). This is problematic for countries like Guatemala, where 71% of households burn waste as a primary means of disposal (Government of the Republic of Guatemala, 2019).

Plastic waste incineration is a critical, but understudied, public health and environmental hazard. Plastic combustion releases toxic black smoke that contains carcinogenic (Barabad et al., 2018) and endocrine-disrupting (Salgueiro-González et al., 2015) compounds. While studies suggest that low levels of bisphenols and phthalates from plastics disrupt neurodevelopment (Mustieles and Fernández, 2020), endocrine (Rochester, 2013), and reproductive function (Miodovnik et al., 2014), no existing literature examines the exposure to these compounds in women of reproductive age who burn their household plastic waste. Specifically, no plastics-focused intervention studies have been implemented in this population. In addition, there are no estimates of gridded emissions from household-level plastic incineration for air quality modeling in Central America.

Our work is theoretically guided by the Behavioral Change Wheel (BCW) (Michie et al., 2011) and the RE-AIM Framework (Glasgow et al., 2019, 1999). The BCW will be used to plan, implement and evaluate success or failures and implementation fidelity of interventions identified from

community working groups. We will apply the RE-AIM framework to evaluate variation in intervention reach, effectiveness, adoption, and maintenance.

4. Study Primary Endpoints

1. Change in $PM_{2.5}$ exposure
   [Time Frame: Baseline, 4-5 months, and 12 –13 months]
   Air pollution exposure will be assessed using repeated 24-hour measurements. This study will compare differences in personal $PM_{2.5}$ among women participating in the two study arms

2. Change in black carbon (BC) exposure
   [Time Frame: Baseline, 4-5 months, and 12-13 months]
   Air pollution exposure will be assessed using repeated 24-hour measurements. This study will compare differences in personal BC exposure among women participating in the two study arms.

3. Change in urinary bisphenols
   [Time Frame: Baseline, 4-5 months, and 12-13 months]
   To assess urinary biomarkers of exposure to plastic combustion, this study will compare differences in bisphenol concentrations among women participating in the two study arms.

4. Change in urinary phthalates
   [Time Frame: Baseline, 4-5 months, and 12-13 months]
   To assess urinary biomarkers of exposure to plastic combustion, this study will compare differences in phthalate concentrations among women participating in the two study arms.

5. Change in urinary polycyclic aromatic hydrocarbons (PAHs)
   [Time Frame: Baseline, 4-5 months, and 12-13 months]
   To assess urinary biomarkers of exposure to combustion by-products, including plastic, this study will compare differences in polycyclic aromatic hydrocarbon levels among women participating in the two study arms.

6. Change in urinary volatile organic compounds (VOCs)
   [Time Frame: Baseline, 4-5 months, and 12-13 months]
   To assess urinary biomarkers of exposure to combustion by-products, including plastic, this study will compare differences in volatile organic compound levels within and between women participating in the two study arms.

5. Study Secondary Endpoints

1. Reach of intervention using the RE-AIM implementation science framework

[Time Frame: 4-5 months and 12-13 months]
Using the Reach, Effectiveness, Adoption, Implementation, and Maintenance (RE-AIM) implementation science framework, assess the reach of the intervention in the intervention arm, as measured by: 1) the number and proportion of total invited participants who attend working groups; 2) the number and proportion of participants' household members who engage in working group activities; and 3) the number of intervention village members who engage in working group activities.

2. Effectiveness of intervention using the RE-AIM implementation science framework
[Time Frame: 4-5 months and 12-13 months]
Using the Reach, Effectiveness, Adoption, Implementation, and Maintenance (RE-AIM) implementation science framework, assess the effectiveness of the intervention by measuring the number of intervention group participants who report behavior changes. These changes are categorized as "high" or "low" behaviors based on factors such as working group attendance, engagement in activities, and reported reductions in plastic burning.

3. Enablers and barriers to the adoption of the intervention using the RE-AIM implementation science framework
[Time Frame: 13 months
Using the Reach, Effectiveness, Adoption, Implementation, and Maintenance (RE-AIM) implementation science framework, assess the enablers and barriers to the adoption of the intervention using qualitative data collected from focus groups and interviews with participants and environmental *promotoras.* This will help identify the factors influencing the uptake of the intervention at the village level. There are no units of measure.

4. Adoption of the intervention by direct observation using the RE-AIM implementation science framework
[Time Frame: 13 months]
Using the Reach, Effectiveness, Adoption, Implementation, and Maintenance (RE-AIM) implementation science framework, assess the adoption of the intervention through direct observations of intervention activities, using qualitative data analysis to evaluate the extent of implementation at the community level. There are no units of measure.

5. Maintenance of the intervention using the RE-AIM implementation science framework
[Time Frame: After 13 months]
Using the Reach, Effectiveness, Adoption, Implementation, and Maintenance (RE-AIM) implementation science framework, assess the maintenance (sustainment of intervention strategies) at the participant, household and village level, as measured by: 1) number and proportion of participants who report no

longer burning plastic in household fires (a measure of de-adoption); 2) number and proportion of households who continue their involvement in intervention activities at the village level after the working groups are completed; and 3) number of community members and organizations who have independently maintained, or established, similar intervention activities in the communities after the working groups are completed in the intervention arm.

6. Study Tertiary Endpoints

1. Change in Health-related Quality of Life (HRQOL) Score
   [Time Frame: Baseline, 4-5 months, and 12-13 months]
   Using the HRQOL instrument, compare differences in quality of life among women participating in the two study arms. The HRQOL consists of 4 questions measuring: a) overall general health (5 items - excellent, very good, good, fair, or poor; used independently, ordinal outcome); b) number of days physical health was not good in the last 30 days; c) number of days mental health was not good in the last 30 days (b and c are summed together, not to exceed 30 days, continuous outcome); and d) number of days poor physical or mental health limited daily activities in the last 30 days (used independently, continuous outcome). Higher scores typically represent poorer health and quality of life, with individuals reporting more health-related problems and worse functioning in daily activities, mental well-being, and social interactions. Lower scores suggest better health status, with individuals reporting fewer limitations or challenges in their physical, emotional, or social well-being.
2. Change in Household Decision Making
   [Time Frame: Baseline, 4-5 months, and 12-13 months]
   Using a 7-part instrument, compare differences in household decision making (e.g., spending earned money, major and minor purchases, job decisions, visiting others) among women participating in the two study arms. The responses are coded on a 4-point scale (respondent's sole decision; husband's decision; joint decision between respondent and husband; other family member makes decision). Scores range from 7 to 28; the lower the score, the greater the woman's agency to make household decisions.

3. Change in New General Self-Efficacy Scale (NGSE) Score
   [Time Frame: Baseline, 4-5 months, and 12-13 months]
   Using the New General Self-Efficacy Scale (NGSE), an 8-part instrument, compare differences in self-efficacy (e.g., problem solving, goal setting, confidence, resourcefulness) among women participating in the two study arms. Each item is rated on a 5-point scale (strongly disagree; disagree; neither agree nor disagree; agree; strongly agree). Scores range from 5 to 40; the higher the score, the greater the individual's generalized self-efficacy belief.

4. Change in Short Social Capital Assessment Tool (SASCAT) Score
   [Time Frame: Baseline, 4-5 months, and 12-13 months]
   Using the 12-part Short Social Capital Assessment Tool, compare differences in group Membership, citizenship and cognitive social capital (trust) among women participating in the two study arms. Responses are Yes/No, and scoring ranges from 0-37. The responses are summed to calculate a total social capital score, with higher scores indicating higher levels of social capital, signifying more support, trust, and participation in community networks.

5. Change in Community Mobilization Scale Score
   [Time Frame: Baseline, 4-5 months, and 12-13 months]
   Using the Community Mobilization Scale, compare differences in the Critical Consciousness sub-scale (modified 9-item instrument) and the Collective Action sub-scale (modified 2-item instrument) among women participating in the two study arms. The Critical Consciousness sub-scale score is measured using a 3-point Likert scale (agree, somewhat agree, disagree; scores ranging from 9-27, measured continuously). The Collective Action sub-scale asks about the number of times the participant and community have worked on community problems in the last 3 months and is measured continuously. For both scales, higher scores indicate higher levels of mobilization.

6. Change in the quantification of emissions estimates of air pollutants from plastic incineration
   [Time Frame: Baseline, 4-5 months, and 12-13 months]
   Using filter-based antimony (Sb) and 1,3,5-Triphenylbenzene (TPB) as tracers of plastic burning and collecting household plastic waste, apportion $PM_{2.5}$ and quantify emissions estimates of air pollutants from plastic incineration and assess effects of potential emissions reduction on air quality with a chemical transport model.

7. Study Intervention/Design

Based on a Baseline Assessment survey in the rural Xalapán region of Jalapa, Guatemala, we will select eight pairs of communities matched on prevalence of plastic burning, population size and density as well as proximity to a main road. The paired villages will be non-contiguous to prevent emissions from control villages affecting biomarker results in intervention villages. Using a village-level cluster randomized controlled trial design, we will randomly select one village within each matched pair to receive a working group intervention to develop strategies to reduce pollution from plastic waste. In a chosen community randomization location, field team members will meet with village members, usually village leaders, who are interested in the randomization process. The field team member will remind those present of the study goals and as an example, show the contents of 2 (unsealed) sample envelopes, one containing a control assignment, the

other an intervention assignment. Then the village members will select an envelope from the actual (sealed) randomization envelopes.

Prior to village randomization, we will revisit the homes of women of reproductive age who reported burning plastic trash as a primary form of waste disposal during the Baseline Assessment in the formative phase. We will screen and consent these women to participate in the main trial. If women are not home, or do not consent to participate in the main trial, the field team members will choose the closest house to the left (when standing in the door, looking to the left) of the baseline house, and so on, until an eligible participant is identified. After consenting to participate in the main trial, these 400 women will participate in interviews, personal air monitoring, and urine biomonitoring at baseline and at 4-5 and 12-13 months of follow-up during the trial period. A subset of 100 women in 4 villages will participate in personal environmental chemical exposure monitoring via silicone wristbands at baseline and at 4-5 months of follow-up. Silicone wristbands are novel passive sampling devices that measure cumulative personal exposure to a wide variety of chemical mixtures, include polycyclic aromatic hydrocarbons (PAHs), polychlorinated biphenyls (PCBs), phthalates and non-phthalate plasticizers, phenols, pesticides, and brominated flame retardants (Hamzai et al., 2022; Samon et al., 2022). These wristbands are non-invasive and inexpensive (O'Connell et al., 2014). *More details on the procedures are described in Section 8.* In the intervention villages, 200 women will be invited to participate in the community working groups.

<u>*Intervention:*</u>

The community working group is a behavioral intervention. Participants over the age of 15 from the intervention villages will be invited to participate in 12-week working group sessions outlined in **Table 1**. We will seek advice from village leaders about targeting recruitment to others, including men, since from our experience these may contribute to village-level change. Recruitment will occur through word-of-mouth and at informational meetings.

Research team local fieldworkers will work with village members to implement a 12-week working group series. Eight core modules – the "essential ingredients" (Cohen et al., 2008) and four periphery modules -- that we posit will increase participant responsiveness to implement and sustain an intervention strategy in the following 9 months – will be implemented. One to three environmental health community workers, or *promotoras*, per intervention village will be identified and will encourage and monitor the women engaged in the intervention group. For the periphery modules (weeks 9-12), participants will identify interventions to reduce plastic waste, its combustion and exposures that are important to them. Participants will brainstorm on individual and community actions they can take. Participants will prioritize one activity that can realistically be achieved over the next 9 months, under the guidance of the *promotoras*, consisting of, for example: 1) starting an organic compost pile free of plastic waste, led by an agricultural ministry member; 2) training on community recycling, focusing on plastics, by a local recycler; 3) making organic soaps, which can be used for personal grooming and washing clothes, eliminating plastic packaging; and 4) creating materials out of plastic, like soft drink bottle planters, or coin purses made of crocheted plastic bags. Recognizing that households want to generate income, the *promotora* training will include information on marketing and record-keeping. *Promotoras* and fieldworkers will have weekly meetings to discuss progress with

activities, including strategies to overcome bottlenecks to implementation during this period. We aim to maintain adherence to core modules through strict protocols, while permitting flexibility of the periphery components, to allow integration into community practices over the 9-month period. See **Table 1** for a description of content for each module.

Table 1. Format of 12-week community working group sessions with participating *promotoras*

	Week	Theme	Components
ESSENTIAL ELEMENTS	1	Identification of main problems of solid waste management	Plastic use and current solid waste practices<br>Alternatives to plastic burning, like recycling
	2	A world of plastic	Sources of contamination (air, land, food)
	3	Plastics in waterways and oceans	River-ocean flow of plastic; plastic ban in Guatemala
	4	Health effects of exposure to burning plastic	Dangers of burning plastics in household fires
	5	Sustainable alternatives to plastic litter	Alternatives to avoid plastic litter
	6	Recycling plastic	Discuss recycling, sorting recyclable materials
	7	Environmental justice and sustainability	Community clean-up/collecting recyclables<br>Ideas and activities reusing/repurposing plastics
	8	Community organization; collective actions	Brainstorming projects to reduce plastic burning
CUSTOM	9-12	Dynamic group work selects community activities. ***Promotoras***, community intervention representatives and external resources develop & support reduced plastic burning by reducing use, repurposing, reusing, or recycling.	
	12-52	Weekly meetings with village leaders, ***promotoras*** & community intervention representatives to support activities. Evaluate activities and address bottlenecks to success using **RE-AIM**.	

Duration of follow-up period:

The follow up period for both intervention and control participants included in this study will be about 12-13 months. Each community within a matched pair will be followed during the same 12-13 month period, to control for seasonal and secular trends in plastic burning. Participants will be invited back to participate in intervention fairs that will occur during the Year 5 dissemination phase.

8. Procedures Involved

Overview: Data will be obtained directly from participants through interviews, questionnaires, focus groups or observations as described below. Urine and air pollution samples will be collected from women who participate in the biomarker study. Unless we obtain future funding, there are no plans for long-term follow-up once the data collection procedures have

ended in Year 5. We will not be deceiving participants about the intervention; the consent states the purpose of the study and they will know whether they were assigned to a control or intervention village. We will debrief participants about the overall study findings as described below in the evaluation section (Year 5). Participants will not be exposed to undue stress from procedures described as follows:

FORMATIVE PHASE (YEAR 1)

Formative phase (Year 1)

Baseline Assessment: We will conduct a baseline assessment of plastic waste burning and waste management practices and socio-demographic determinants. Going door-to-door, trained local fieldworkers will verbally consent and administer a 20-30 minute survey to the primary cook in household from the 1,630 households in ~37 villages in the Xalapán region in the Department of Jalapa, Guatemala identified from the 2021 Ministry of Health Census The survey will ask: 1) household size/composition; 2) stove type(s), location; 3) location and frequency of burning waste, including plastic; 4) types and amount of waste; 5) recycling practices, knowledge and capabilities; 7) opportunities to dispose of waste besides burning; 8) motivation to improve environment; 9) interest in participating in future community working groups; and 10) contact information. With six fieldworkers, conducting ~30 surveys per day, this activity will be completed in < 3 months.

Community dynamic working group refinement: We will develop the content of the working group material by observing participants who burn plastic trash in their households during the baseline assessment visit, conducting open-ended surveys with up to 50 participants, and conducting 10-20 key informant interviews with community stakeholders who recycle, dispose or repurpose plastic trash. Standard operating procedure manuals will be developed to standardize the intervention package for the main study.

Piloting of the intervention package for community dynamic working groups: We will pilot and further refine the working groups, tested in one village with up to 100 participants. Eight core and four peripheral modules will focus on the topics described in **Section 7** and **Table 1**.

Air pollution monitoring of plastic trash burning: We will identify 6-12 houses from the baseline assessment who meet the eligibility criteria for the biomonitoring study, and who report burning trash in household fires. The purpose of this pilot is to test the detection levels of 1,3,5-Triphenylbenzene (TPB) and a series of 25 PAHs on 37 mm quartz filters and filter-based PM2.5, BC, and elements (including antimony) for source apportionment on 37 mm polytetrafluoroethylene (PTFE) filters under real-world conditions. We will collect 24-hour personal and kitchen measures in 6-12 unique homes twice. Filter-based antimony and TPB and are known tracers of plastic incineration found in atmospheric particulate matter ($PM_{2.5}$). Furthermore, Sb is unique to garbage burning, and especially to plastic burning. We will therefore use Sb and TPB to quantify estimates of air pollutants due to plastic burning.

Prior to deployment in the field, PTFE filters will be pre-weighed and placed in cassettes in a clean lab room. The quartz filters will be pre-baked at 550 °C and placed in cassettes in a clean lab

room. For each week, there will be one lab blank sample that will be included for both quartz and PTFE filters.

In the field, personal exposure will be assessed by 2 fieldworkers visiting the participant's home and asking women to wear two lightweight, unobtrusive personal $PM_{2.5}$ monitors for 24 hours. We will co-locate two UPAS monitors in the kitchen for the same 24-hour period to measure kitchen concentrations.

Round 1 (co-location round):

Personal:

- On day 1, we will place the personal monitoring devices in pockets of aprons provided to them by project staff that women will wear. Two co-located samplers will be worn by each participant, one Casella APEX pump and filter (quartz) and one UPAS and filter (PTFE). We will instruct them to place the monitor next to their bed when they are sleeping.
- On day 2, we will retrieve the equipment and administer a survey about daily activities and compliance with wearing the monitors. The monitors are lightweight, ergonomic design, motion sensor to detect compliance, quiet motor, durability, and long battery life.

Kitchen:

- On day 1, four co-located samplers will also be placed in the kitchen at a height of 1.5 meters and 1 meter from the biomass stove, consisting of two Casella APEX pump and filters (one PTFE and one quartz) and two Casella (duplicates, PTFE).

Two types of particle size selection devices will be used to sample PM2.5:

1. the Harvard impactor designed to collect PM2.5 at 3 LPM flow rate will be used with PTFE and quartz filters and a Casella pump, and
2. the UPAS monitor designed to collect PM2.5 at 1 LPM flow rate will be used only with Teflon filters

Round 2 (UPAS/Quartz round):

All the same procedures described in Round 1 apply, except that:

Personal:

- On day 1, we will place the personal monitoring devices in pockets of aprons provided to them by project staff that women will wear. Two co-located samplers will be worn by each participant, one UPAS and quartz filter and one UPAS and PTFE filter, both with drain discs.
- The UPAS monitor designed to collect PM2.5 at 1 LPM flow rate will be used with the PTFE and quartz filters

Kitchen:

- On day 1, two co-located samplers will also be placed in the kitchen at a height of 1.5 meters and 1 meter from the biomass stove, consisting of two Casella APEX pump and filters (one PTFE and one quartz).
- The Harvard impactor designed to collect PM2.5 at 3 LPM flow rate will be used with PTFE and quartz filters and the Casella pumps.

We will collect one PTFE filter field blank and one quartz filter field blank on each sampling day. There will be a total of 78 Teflon filters (includes lab 6 blanks) and 30 quartz filters (includes lab 6 blanks) for laboratory analyses. For lab blanks, we will open the petri dish with the filter inside, place it in the respective monitor, and then remove the filter and place it back in the petri dish. This will be done in the morning when monitors are being charged in the lab on Tuesday and Thursday or Monday and Wednesday depending on the sampling week.

Urine collection: To pilot the sample collection, storage procedures and sample analysis, at the second 24-hour visit to each of the households, we will collect urine from the participants. Women will be provided with a standard sterilized urine collection cup and a vaccine cooler with ice packs during day 1 of the second visit and instructed to collect their first morning void the following day and store the sample in the provided cooler. Women will be told to briefly initiate the urine stream before collecting the remainder of the urine void in the cup (clean catch). Samples will be picked up by the field team when they come to pick up the exposure monitoring equipment on day 2. The time of urine collection will be recorded on the collection log and the total volume collected will be estimated and recorded. Within 4 hours of collection, the urine will be aliquoted to 4 cryovials (2*4-mL tubes with 3 mL urine; and 2*10-mL tubes with 7.5 mL urine) and labeled at the field office. The labelled samples will be frozen and kept at -20°C at the Jalapa field office and transferred every two weeks to UVG for storage at -70°C. Urine will be shipped using blue ice packs to the Biomarker Core at Emory.

MAIN TRIAL (YEARS 2-4)

Prior to Randomization

Rapid Ethnographic Assessment (Community Diagnosis)

We will conduct a rapid ethnographic assessment in each of the 8 intervention and 8 control communities *prior to the baseline measurements and prior to the determination of study arm* in each of the villages. We will use qualitative and quantitative research methods, including observations, key informant (personal and group) interviews, visual media (pictures and videos), sociograms, and free listing as key strategies to quickly identify and address topics related to solid waste management with a focus on plastic burning. We will identify key organizations and community leaders that would be instrumental in the development of intervention activities. A rapid ethnographic community assessment, right before the intervention activities commence, allows the use of a variety of data sources in a range of settings. The process permits a rapid collection of community insights about a specific issue to identify unique aspects that could be barriers, facilitators, and motivators around plastic waste management. This community

assessment can help us to address concerns and issues ahead of time and close information gaps before we start with the intervention.

Identification of Women for the Biomonitoring Study

In the selected 16 villages, we will return to revisit the homes of women of reproductive age who reported burning plastic trash as a primary form of waste disposal during the baseline assessment in the formative phase. We will screen and consent these women to participate in the main trial. If women are not home, or do not consent to participate in the main trial, the field team members will choose the closest house to the left (when standing in the door, looking to the left) of the baseline house, and so on, until an eligible participant is identified. This will be done to consent and enroll the 25 women from each village who expressed initial interest in the biomonitoring study. Since the villages will be invited to participate sequentially (see timeline in Appendix A), this follow-up assessment will be staggered over time as each pair of villages enters into the study. This follow-up survey will ask about: 1) location and frequency of burning waste, including plastic; 2) types and amount of waste that is burned or recycled; 3) interest in participating in future community working groups; and 4) contact information. Additionally, we will administer the Health-related Quality of Life instrument (Hennessy et al., 1994), Household Decision Making (Martínez-Restrepo et al., 2017), New General Self-Efficacy Scale (Chen et al., 2001), Short Social Capital Assessment Tool (De Silva et al., 2006), and the Community Mobilization Scale (Lippman et al., 2016).

The outcome assessments for the 400 women in the biomonitoring group include interviews, focus groups, personal air pollution exposure assessment, urine exposure biomarkers, and quantification of usual plastic burning. These assessments will generally be performed at baseline (prior to randomization) and at 4-5 and 12-13 months of follow-up.

Interview: Following recruitment and the obtention of written informed consent, a baseline survey will be administered by 2 fieldworkers. We will administer verbal surveys asking about household socio-demographics, tobacco exposure, sources of household smoke, and food/products containing PAH/VOC/BPA/phthalates. The primary question is: “do you burn plastic waste in your home?” Fieldworkers will interview participants in a private room to maintain privacy of information. Similar (but shorter) interviews will be conducted at the 4-5 month and 12-13 month study visits.

Air Pollution Sampling: All 400 women in the biomonitoring study will be participate in personal monitoring of $PM_{2.5}$ and BC at baseline, 4-5 months and 12-13 months. In the field, personal exposure will be assessed by 2 fieldworkers visiting the participant's home and asking women to wear lightweight, unobtrusive personal $PM_{2.5}$ monitors (Ultrasonic Personal Air Sensor - UPAS) for 24 hours. On day 1, we will place the personal monitoring devices in pockets of aprons provided by project staff that women will wear. UPAS samplers will be worn by each participant, with either quartz or teflon filters as described in **Table 2**.

In a subset of 300 women, we will measure concentrations of Sb on PTFE filters twice (300 at baseline and 300 at 4 months; 150 each from intervention and control) using XRF analysis of elements ranging from magnesium (Mg) through lead (Pb), including Sb, a tracer of plastic combustion.

In a subset of 60 women, we will measure 1,3,5-triphenylbenzene and a series of 25 PAHs on quartz filters twice (60 at baseline and 60 at 4 months; 30 each from intervention and control).

Filters will be stored in the laboratory refrigerator until weighing or shipment to a US laboratory.

Table 2. Personal sampling strategy per village, per week

	1 Teflon filter	2 Teflon filters	1 Quartz filter	2 Quartz filters	Teflon	Quartz
25 women	17	3	4	1		
Lab blanks					2	1

Urine Biomarker Assessment: On day 1, when air pollution sampling begins, trained fieldworkers will instruct women on urine clean-catch procedures and provide a sterile urine collection cup and a vaccine cooler with ice packs. Women will be instructed to collect a first-morning urine void sample on day 2. At the home visit on day 2, local fieldworkers will pick up the urine specimen, record the time of urine collection, time of previous urine void (if known), and the total volume of the sample in the collection log. Labelled specimens will be secured in a cooler with ice packs and transported in project vehicles to the field laboratory. Urine will be refrigerated within 4 hours of collection.

__Urine sample processing and storage:__ In the field laboratory, 21 ml of urine will be transferred to 4 labelled cryovials (two 4 ml tubes with 3 ml urine; and two 10 ml tubes with 7.5 ml urine). The labelled samples will be frozen and kept at -20°C at the Jalapa field office and transferred every two weeks to UVG in a cooler with ice packs for storage at -70°C until shipped on blue ice to the Barr Lab at Emory where they will be stored at -80°C until analysis. Urine samples will be aliquoted into 4 cryovials to enable access to samples without repeated thaw-refreeze cycles to maintain sample integrity.

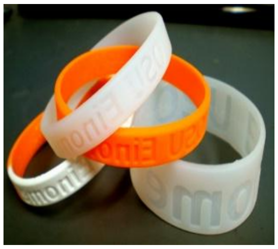

Silicone Wristbands for Exposure Monitoring: A subset of 100 women in 4 villages will be given silicone wristbands designed to monitor personal exposure to environmental chemicals, including PAHs, at the baseline and the 4-5 month visits (n=200 wristbands for analysis), at the same time as the personal exposure assessment and urine collection. Silicone wristbands are made of a stretchy silicone rubber material (**see Figure**) that slips over the wrist and causes no discomfort to the participants. These sampling devices are unique, compared to other methods of exposure sampling (e.g., urine, blood, pump-and-filter air pollution monitoring), in that they are less burdensome to the participant, and are emerging as an easy-to-wear sampling device to measure cumulative exposure to a wide range of chemicals (Hamzai et al., 2022). Organic chemicals in the air, soil and water can be sequestered into the silicone media via diffusion, and uptake depends on chemical concentrations that a person is exposed to, and the length of time the wristband is worn. Inhalation and dermal exposures are both captured in the wristband media (Doherty et al., 2020). The most frequent sampling time is

7 days, although these devices have been used for 4-hours to 30-days (Wacławik et al., 2022). For the purposes of this study, participants will be asked to wear the bracelet for one week.

Silicone wristband processing, deployment, and storage: Prior to distribution to participants, silicone wristbands will be cleaned at the Stapleton lab at Duke University using 2 12-hour Soxhlet extractions with 1:1 ethyl acetate/hexane (v/v) followed by 1:1 ethyl acetate/methanol (v/v), a method adapted from O'Connell et al. (O'Connell et al., 2014). After cleaning, wristbands will be dried in a vacuum oven for 24 hours and then transferred to air-tight aluminum tins until deployment. Each tin will be placed inside a mylar resealable bag. Wristbands will be shipped to the Guatemala field station as such.

At the participant's home, the mylar bag and sampling tin will be opened and the wristband will be removed and placed on the woman's wrist. The date and time that the wristband was placed will be entered on the data collection form. Women will be instructed to wear the wristband continuously for 8 consecutive days and nights (e.g., Monday 9 am to Monday 9 am), during all daily activities, including bathing, eating and sleeping, and will be told the exact day and time of day to remove the wristband. After removal, participants will be instructed to place the wristband in the aluminum tin, seal it tightly, and then return the tin to the original mylar bag, making sure to write the date and time of removal on the label affixed to the bag. They will be told to store the bag in a secure place until the fieldworker returns. At the home visit on day 8, fieldworkers will conduct a brief survey about personal care product use and potential exposures to chemicals that might influence wristband results. They will also ask about compliance while wearing the wristband, for example, if it fell off or was removed for any length of time.

Labelled wristband containers will be transported in project vehicles to the field laboratory and stored at -20°C. Within 2 weeks, they will be transferred to UVG and securely stored at -20°C until shipped to a laboratory at Duke University, where they will be stored at -20°C until analysis.

Emission Estimates from Trash Burning: We will ask participating households (5 participating households randomly selected from each village) to collect all the waste that would have been disposed of over a 1-week period, at baseline, at 4-5 months and at 12-13 months of follow-up. They will collect waste in bags we provide. After our field team classifies the waste, they will separately weigh the plastic waste and the remainder of the waste and then transport the waste to the municipal dump and/or the recycling center.

Kitchen and outdoor fire air pollution sampling: Kitchen gravimetric $PM_{2.5}$ measurements will be taken using UPAS pumps and filters (time weighted average, on both PTFE and quartz 37 mm filters) at the same time as the personal exposure assessment and urine collection. The kitchen UPAS monitor will be affixed to a kitchen wall 150 cm from the floor and 100 cm from the center of the fire. For open fire monitoring, 2 tripods with UPAS monitors will be set up (1 with quartz and 1 with PTFE filters), 1 meter from the outdoor fire. The fire will be monitored at a high flow rate (3L/min) for 60 minutes. We will note behaviors related to starting, stoking and if a woman who is wearing personal monitors is standing by the outdoor fire. This will be collected in a randomly selected subset of 80 homes.

Intervention villages: We will evaluate implementation of program activities in each of the 8 intervention communities as follows:

First, we will conduct a set of activities with the community environmental *promotoras* and participants who were involved in the intervention activities (e.g., composting, recycling) that were chosen to reduce or avoid the burning of plastic waste in their communities. Meetings will be organized by the ECOLECTIVOS field team together with the *promotoras*. This set of activities will be held three times (months 6, 9, and 12 during the intervention period), starting after the working group sessions are finished. Each time, we will do three activities: (1) conduct individual interviews with *promotoras* and representatives of the interventions. These interviews will occur immediately before the intervention evaluation meetings; (2) conduct intervention evaluation follow-up meetings with people who are working on each intervention in each village; and (3) conduct a team evaluation, where our team works together to evaluate the progress of each *promotora* and representative of the intervention. For all three of these activities, which will occur on the same day, we will ask open-ended questions about: 1) progress in relation to what was planned; 2) changes in timelines and objectives of the proposed activities; 3) expected costs of the proposed tasks; 4) proposed objectives and future goals; 5) active participation in activities by members; and 6) involvement of other community members in intervention activities. To evaluate the intervention activity in (2), we will tailor questions specifically to the chosen intervention activity and questions will build progressively in subsequent meetings (e.g., answers to questions in meeting 1 will lead to focused questions in meeting 2). All three of these activities will allow us to assess the *promotoras* as well as the intervention activities and will offer opportunities for addressing bottlenecks to achieve successes.

Second, we will conduct focus groups with 8-10 participants of the working group sessions from each intervention village (n = 60-80) to evaluate the working group sessions, at month 12-13. Through a series of reflective questions, participants will be asked to share their perspectives, and experiences at the personal, family and community level of the ECOLECTIVOS curricular program. Examples of questions will focus on: 1) content learned; 2) motivations to enact activities to reduce plastic burning; 3) changes made in their home to reduce consumption and burning of plastic waste; and 4) the sustainability of interventions after ECOLECTIVOS ends.

Audio-recording: Key-informant interviews conducted during the formative phase and focus groups, individual interviews and follow-up meetings conducted during the main trial phase will be audio-recorded and transcribed. Transcripts, notes from participant observations and field notes will be coded and analyzed using nVivo software. Audio recordings will be stored on password-protected computers or on a secure cloud storage server.

Ambient monitoring: We will set up 5 ambient monitors (MODULAIR-PM, produced by QuantAQ, Inc, Somerville, Massachusetts), which measure size-resolved particle number concentrations (0.35 µm-40.0 µm) and measurements particulate matter (PM) at different diameters (PM_1, $PM_{2.5}$, and PM_{10}) (range: 0-2,000 µg/m3), as well as temperature and relative humidity. Data is transmitted via cellular phone data services and can be viewed globally via the QuantAQ Cloud platform. We will deploy them in participating Guatemala village fixed locations (e.g., schools, community health centers) that are distributed across the region in 5 of the 16 villages. This will allow us to track ambient $PM_{2.5}$ and temperature data over 4 years and link them to the 400

women's personal $PM_{2.5}$ and temperature data. Furthermore, we will be able to better assess the impact of our intervention on ambient air quality and allow us to evaluate the model simulations using the Weather Research and Forecasting model with chemistry (WRF-Chem). With these sensors, we will be able to quantify the health benefits of reducing trash burning, as a climate-health co-benefit. We will also investigate the potential change in air quality over the 4-year time period.

EVALUATION AND DISSEMINATION (YEAR 5)

We will evaluate community dynamic working group activities in year 5 with input from our *promotoras,* village leaders and stakeholders, using RE-AIM informed tool-kit materials developed for improving sustainability of community interventions (see **Table 3**) (Estabrooks et al., 2011). The RE-AIM framework will allow us to assess variation in intervention reach, effectiveness, adoption, and maintenance. We will measure individual-level impacts on all working group participants over the age of 15 years of age. We will evaluate the community-level changes in the 400 participants in the exposure study, which will inform us about community-level impact among women who attend and don't attend the working groups.

The research team will share results obtained from the study and collect inputs from participants at dissemination meetings conducted in local communities in Year 5. We will discuss successful ways communities found to reduce plastic trash burning and plastic usage as a result of the workshop intervention (Aim 1) and invite local policymakers to attend meetings to learn about the results. We will evaluate health through differences in urinary metabolites of PAHs/VOCs/BPA/phthalates from baseline to 4-5 and 12-13 months after baseline (Aim 2), analyzing differences both within-groups and between-groups of women from the workshop intervention villages and the control villages. These aggregated results will be presented at dissemination meetings in Year 5. We will present air quality modeling results (Aim 3) at the dissemination meetings. We will present findings in such a way that they are understandable to lay audiences.

We will assess the effect of community working group interventions on the amount of plastic burning based on reported and observed behaviors that change from baseline to 4-5 months and 12-13 months, like reducing, reusing or recycling (effectiveness), with additional measures included to evaluate adoption, reach, and sustainability of intervention strategies (see **Table 3** for example using plastic trash and recycling behaviors). We will measure implementation quality, dose and fidelity of adherence to working group content, based on working group protocols; exposure or dose, measured by coverage (attendance frequency, duration and completion); and training and quality of teaching by fieldworkers and *promotoras* through direct observation. Participant variation in responsiveness (reach) will be measured by level of involvement in core and periphery activities and understanding of working group strategies (Kechter et al., 2019); and program differentiation by assessing which program components are essential for success (Carroll et al., 2007).

Table 3. Exemplar of COM-B and RE-AIM frameworks to Evaluate Individual and Community Behavior Change Among Working Group Participants, Households and Villages

Year 1	Years 2-4	Years 2-5
**Examples of COM-B: Inquiry: Formative Assessment**	**Effectiveness Outcomes: Evaluation of Individual-Level Household Behaviors**	**Reach, Adoption, Maintenance Outcomes: Evaluation of Community-Level Changes**
**Psychological/Physical Capability**		
List recyclable items in your home. (TDF* domain=*Knowledge*)	N & % able to separate different plastic recyclables correctly	% Households separating recyclables correctly (*adoption*); variation in recycling by village characteristics (*reach*)
Have you recycled? Have you burned household trash? Plastic? (TDF domain=*Knowledge*)	N & % stating benefits from recycling/not burning plastic waste	% Households dis-adopting burning of plastics and level of dis-adoption (*adoption*)
If you became a village recycler, what would you need to be successful? (TDF domains =*Knowledge; Memory, attention & decision processes; Env. context & resources*)	N & % earning income from recycling	% Recycling waste with local recyclers (*adoption*); variation in recycling by village characteristics (*reach*)
Have you heard about plastic trash accumulating in the environment on TV or social media? (TDF domain= *Memory, Attention, Decisions*)	N & % recalling actionable info. re burning plastic on media	Variation in recall by village characteristics (*reach*)
If your household stopped burning trash/plastic, what would happen? (TDF domain=*Outcome Expectancy*)	N & % no longer burning plastic in indoor fires/in outdoor fires	% of women reporting that their household no longer burns plastic (*adoption*); variation in burning by village characteristics (*reach*)
**Reflective/Automatic Motivation**		
What have other family members said about your participation in the educational sessions? (TDF domain=*Social Influences*)	N & % perceiving that participating in educational sessions has improved their status within the home	Variation in perceptions by village characteristics (*reach*)
What do you think about plastic trash/burning in your community? How does recycling benefit you? (TDF domain=*Optimism; Beliefs about consequences; Emotion*)	N & % attributing stopping burning trash for health and community goals	Variation of villages attributing stopping burning trash to health and community goals by village characteristics (*reach*)
Does burning plastic harm health? What happens if we don't take action to reduce plastic?	N & % reporting health consequences from exposure to burning plastic	Variation of villages attributing stopping burning trash to health outcomes by village characteristics (*reach*)
Is not burning trash/plastic a priority? (TDF domain=*Social Influences; Goals*)	N & % report importance of not burning plastic	Variation in villages participating in a municipal waste program and reduced trash burning *(reach)*
**Physical/Social Opportunity**		
What comm. resources are needed to "manage plastic well"? (TDF domain=*Env. Context/resources*)	N & % accessing materials needed to recycle/dispose of waste without burning	Variation in villages accessing recycling materials *(adoption, reach)*
**Opportunity - Social**		
Do people in your community care about trash or trash/plastic burning? (TDF domain=*Social Influences; Goals*)	N & % stating others have reduced trash/plastic burning	Variation in reporting by village (*adoption*), and by age, gender, etc. (*reach*)

*TDF = Theoretical Domains Framework

9. Data Specimen Banking

URINE SAMPLE PROCESSING AND STORAGE

In the field laboratory, 21 ml of urine will be transferred to 4 cryovials (two 4 ml tubes with 3 ml urine; and two 10 ml tubes with 7.5 ml urine) using QR code labels that link to the participant ID. Samples will be stored at -20°C in the field lab until shipped in a cooler with blue ice packs to the Barr Lab at Emory where they will be stored at -80°C until analysis. Urine samples will be aliquoted into 4 cryovials to enable access to samples without repeated thaw-refreeze cycles to maintain sample integrity. After urinalysis is performed, specimens will be destroyed. Data stored with the sample will include the project name, participant ID, and date of collection. We will not include information about the study arm so that blinded data will be analyzed. If samples are accessed by investigators other than those named in this protocol, data will be requested by the investigators using a data request form and reviewed by the MPIs before approving any requests.

10. Sharing of Results with Participants

We will work with select community leaders, *promotoras*, and stakeholders in Guatemala to develop research report-back strategies to communicate study results to communities and individuals, in line with community interests and motivations. We will work carefully to ensure that expectations are not raised unreasonably. To illustrate this point, by training local fieldworkers, knowledge will be transferred to local community members. All findings will be de-identified and presented at the aggregate level to avoid breeches in confidentiality.

We will disseminate our aggregated findings to local governmental and non-governmental organizations in Jalapa and at the national level in Guatemala to inform intervention programs that combine behavioral intervention evaluation with exposure assessment for policy-relevant solutions in Guatemala and other countries that burn waste as a primary means of disposal. We will prepare materials in English and Spanish. We will conduct webinars in English and Spanish to explain the findings from our project. Our approach is to bring community members, academia, policymakers and other stakeholders, including NGOs and national research institutes, together to provide a place for a joint capacity building and information exchange on issues related to household air pollution, ambient air pollution, and the impact this has on climate change.

11. Study Timeline

The formative phase will occur in Year 1. The Main Trial will occur in Years 2-5. It will take us 4 years to enroll all study participants and complete the main trial. Participants will be in the main trial for 12-13 months. We will complete the evaluation phase in Year 5. See **Table 4** for specific study activities and their duration and timeline. Please see **Appendix A** for village roll-out and sampling procedures.

Table 4: Study activities and timeline

Formative Phase (Year 1)				
	**Activity**	**Participants**	**Information collected**	**Time**
**Aim 1**	Baseline Assessment (BA)	1,630 households in 37 villages	– household/demographic characteristics<br>– waste management/recycling survey<br>– ID 400 women for biomonitoring study<br>– ID one village to invite 100 participants for workshop refinement	One visit<br>20-30 minutes<br>Completed in 3 months
	Workshop refinement/ Definition of working group activities	50 BA participants	– open-ended survey (behavior change wheel questions)	One visit<br>45-60 minutes
		Up to 1,630 BA participants who burn plastic waste	– participant observation in households	One visit (2-4 hours)
		10-20 key informants	– stakeholders, officials interviewed	One interview<br>1-2 hours
	Workshop pilot	100 members of one village	– train to deliver curriculum content<br>– finalize curriculum/procedure manuals<br>– brief surveys before and after meetings	1-1½ hours a week (meetings)<br>14 weeks<br><br>10–15 minute surveys
	Exposure Assessment Pilot	12 households	– 24-hour personal measurement<br>– 24-hour kitchen monitoring	1 day per household, 24-hour monitoring, visits are 30 minutes/day
**Main Trial (Years 2-5)**				
	**Activity**	**Participants**	**Information collected**	**Time**
**Aim 1**	Rapid ethnographic assessment	160 participants (10 per village)	– who in village would be a contact point for our program; who recycles; who works with waste management	45-60 minutes
	Surveys	200 intervention<br>200 control women in biomonitoring	– household/demographic characteristics about exposure to air pollution, including plastic burning	45-60 minutes<br>Baseline, Month 4-5 (after working groups

			– household decision making<br>– health related quality of life<br>– self-efficacy<br>– community mobilization<br>– social capital	end) and Month 12-13
	Community dynamic working groups	-200 participants 8 intervention villages<br>-Other villagers who choose to participate over the age of 15 years	– 8 core modules<br>– 4 periphery modules<br>– evaluate intervention fidelity over 9 months	1-2 hours a week over 12 weeks followed by periodic meetings over 12 months
	Home visits/phone calls by *promotoras*	200 participants 8 intervention villages	– home visits to assess engagement of other participants with the working groups	-1-2 times a month for home visits<br>-1 time a week for phone call
	Individual interviews and follow-up meetings	50-80 *promotoras* and leaders of intervention activities	– interviews and meetings to discuss barriers and enablers of selected intervention activities with those who are implementing them in the communities	1-1 ½ hours a week at 1, 3 and 9 months after the start of intervention activities
	Focus groups	8 focus groups (n=60-80 working group participants)	– working group evaluation	1-1 ½ hours,<br>Month 12-13
	Interviews	4 study team members	– evaluation of *promotoras'* and community intervention representatives' progress	1-1 ½ hours,<br>Month 12-13
**Aim 2**	Personal and Biomonitoring Exposure Study	200 intervention<br>200 control	– household/demographic characteristics<br>– air pollution exposures	1-2 hours on 2 consecutive days Baseline, Months 4-5 and 12-13
	Kitchen and outdoor fire exposure monitoring	40 intervention<br>40 control	– air pollution exposures	24 hours for kitchen monitoring
	Personal environmental chemical exposure monitoring (silicone wristbands)	50 intervention<br>50 control	– environmental chemical exposures	20 minutes on day 1, 7 days inactive (wearing of silicone wristbands), 30 minutes on day 8

				Baseline and Month 4-5
	Ambient monitoring	5 villages (regional)	– air pollution exposures	Continuous measures over 4 months in each village
**Aim 3**	Waste collection; ambient sampling (used to model emissions and regional air quality from burning)	40 intervention<br>40 control	– collect household trash that they would have disposed of for one week<br>– ambient sampling for source apportionment in 16 villages	– 3 hours to collect trash over 1 week at<br>– Baseline, Month 4-5 and Month 12-13<br>– 1-2 weeks in each village, 2-24 hours filters
**Evaluation and Dissemination (Year 5)**				
	**Activity**	**Participants**	**Information collected**	**Time**
**Aims 1-3**	Report-back of findings<br><br>Capacity building activities	Community participants, local stakeholders, regional/ national policy-makers	– revaluate recruitment & retention of participants<br>– evaluate individual and community strategies<br>– evaluate RE-AIM dimensions<br>– disseminate intervention strategies	– 1-hour dissemination meetings in participating villages<br>– 1-hour meetings with local and national authorities

12. Inclusion and Exclusion Criteria

Inclusion and Exclusion Criteria for **Formative Phase**:

Inclusion criteria:
For households in the **baseline assessment survey**, working group refinement, participant observations, and piloting of working groups:

- Age > 18 years
- Lives in study area
- Interview conducted with person who identifies as the primary cook
- People who are at home when we conduct interviews
- Among the baseline assessment survey participants, we will select 12 households that burn trash including plastics inside and outside of their homes.

For **key-informant interviews**:

- Willing to attend interviews
- Identifies as someone who works for an NGO or governmental organization on environmental protection or improvement, such as recycling, or a village leader

<u>Inclusion and Exclusion Criteria for **Main Trial**:</u>

For **community-level workshops**:

- There are no inclusion or exclusion criteria as the whole community will be invited

For **questionnaires administered to workshop attendees**:

<u>*Inclusion criteria:*</u>

- Over the age of 15

For **collection of urine, personal air pollution, and personal environmental chemical exposure samples**:

<u>*Inclusion criteria:*</u>

- Women of reproductive age (> 15 and <44 years, verified by official document)
- Willingness to attend weekly working groups for a 12-week period (if village is selected to be in the intervention arm)
- Willingness to participate in biomonitoring study at baseline, 4-5 months and 12-13 months
- Willingness to wear a silicone wristband for 1 week at baseline and 4-5 months.
- Household uses biomass as primary fuel for cooking
- Participant reports daily participation in household cooking (does not need to be the primary cook)
- Participant reports that plastic is burned in household fires (in cooking stove <u>and</u> outdoors) at least once a week
- Plans to live in the household for the next 12 months (duration of the working groups)

<u>*Exclusion criteria:*</u>

- Inability to consent
- Cognitively impaired or individuals with impaired decision-making capacity
- Pregnant women. However, women who become pregnant during the course of the study may continue to participate.
- Women who report using tobacco products

For ***promotoras***:

<u>*Inclusion criteria:*</u>

- Over the age of 18
- Ability to read and write
- Women who participate in biomonitoring (collection of urine and personal air pollution samples) in the intervention group
- Willingness to make monthly household visits to study participants
- Willingness to make weekly calls to study participants
- Willingness to encourage participation in the activity selected by the village
- Willingness to communicate weekly with the research team

- Willingness to support community meetings to scale up the intervention in intervention and control villages

13. Population

Jalapa has a rural population of 75,134 people in 133 villages; we have chosen the area of Xalapán, which has 29 villages with over 100 households. In this region, almost 50% self-identify as Xinca indigenous people; this proportion is higher in rural areas (Government of the Republic of Guatemala, 2019). The remaining population self-identify as Ladino. All individuals speak Spanish; the Xinca language is extinct. We will exclude participants that are unable to provide consent, individuals < 15 years old, pregnant women at time of enrollment (in the biomarkers study), or cognitively impaired individuals. This is a community-based study and members will be invited to participate in the working groups, with dissemination of results to all participating communities.

14. Vulnerable population

Our study targets communities burdened by high exposures to air pollution. Child participants (ages 15-18) in the study are defined as vulnerable populations under DHHS guidelines. We will recruit non-pregnant women at the beginning of the study; however, women may become pregnant during the course of the study. We will not ask them to do anything that would expose them to risks beyond their usual daily activities.

15. Local Number of Participants

We will select a random subset of 16 villages from among villages in Xalapán, Jalapa, Guatemala identified during the Baseline Assessment conducted in Year 1 (formative phase) using Ministry of Health census data. To identify households in these villages, we will use Google Earth imagery and geographical software to define a sampling frame and digitize building structures. We will randomly select 60 rooftops from each of the 37 sectors using satellite images, for a total of 2,220 potential households. From our previous work we know that 30% of the rooftops were other structures (e.g. abandoned homes, garages); we will thus oversample by 30%. Fieldworkers will visit these structures using hand-held GPS receivers to identify 1,630 households. From these villages we will randomly assign 16 villages to study groups (8 intervention and 8 control, pair-matched based on baseline plastic burning, village size and proximity to a main road, and non-contiguous). We will randomly select 25 women of reproductive age from each village (n =400, ages > 15 and <44 years, who burn plastic waste at least once a week in their household fires) to participate in the biomonitoring described in Aim 2.

16. Recruitment Methods

Going door-to-door at the Baseline Assessment, we will recruit participants. We will record survey data using a secure, web-based data collection program, REDCap, on encrypted tablets. No personal identifiers (address, name) will be recorded on REDCap. A key database with the

name, phone number and GPS coordinates of the household will be kept on a secure password-protected server so that we can return to the households for later trial procedures.

Formative Phase:

1) **Baseline Assessment**: Fieldworkers will go door-to-door and will verbally consent women over 18 years of age who are the primary cooks in their homes. From this sample, we will identify participants for the working group curriculum refinement.

2) **Working Group Refinement:** We will recruit a purposive sample of adult participants identified in the Baseline Assessment to provide input on the curriculum for the working groups that will be conducted during the Main Trial. We will conduct observations in these households to identify strategies to reduce plastic burning in household fires that may not have been mentioned in the questionnaire. We will identify, recruit and conduct in-depth key informant interviews with stakeholders, including recyclers, village authorities, villagers, and ministry officials. Some of these stakeholders have been identified during pilot work in preparation for this study, but others will be recruited through word-of-mouth, visits to villages to explain the study, and through local networks of NGOs and governmental organizations that work on waste recycling and disposal.

3) **Exposure Sampling**: Fieldworkers will identify 6-12 suitable households from the Baseline Assessment that meet the same eligibility criteria for collection of urine and personal air pollution samples described in Section 12 above.

Main Study Aim 1:

Women from the biomonitoring group from the intervention villages will be invited to participate in the 12-week working groups. They will be recruited through the 1,630 households that participated during the baseline assessment. If, in any given village, there are not enough baseline households that meet the eligibility criteria for the biomonitoring trial, we will select additional households by walking to the immediate left of the household (facing away from the house) that participated in the baseline study until a household is identified that has an eligible woman. Each eligible woman can invite a family member, friend or neighbor for a total of up to 50 participants at each working group. If we see attrition over time, we will recruit additional participants using methods similar to our pilot study: through council meetings, churches, or schools. Village-level recruitment and working group activities will be staggered throughout the year, allowing us to account for seasonal variability. One to three community environmental health workers (*promotoras*) will conduct monthly home visits and/or make phone calls to participants, to assess their level of engagement in the working groups and encourage participation. Focus groups with 6-8 members from each intervention village will be conducted at months 12-13 to assess activities that have been successful or not, as well as barriers and enablers of success. Individual interviews and follow-up meetings with *promotoras* and leaders of intervention activities will occur in each intervention village at 1, 3 and 9 months after the start of intervention activities to assess the roll-out of intervention activities, including barriers and enablers of their success. The study team will also evaluate the progress of each *promotora* and community intervention representative at the same 3 timepoints.

Main Study Aims 2 and 3:

We will recruit participants for Aims 2 and 3 from the 400 women of reproductive age from 8 intervention and 8 control villages previously identified during the Baseline Assessment.

17. Withdrawal of Participants

Participants may voluntarily choose to withdraw from any of the study procedures at any time. We have found in our other studies in this region that the withdrawal rate is very low, between 5%-7% over an 18-month period. The only foreseeable event that might necessitate withdrawing a participant from our study without their consent would be if there was a direct harm or concern to the well-being of our field staff or to the participating woman (e.g. her husband does not agree with study procedures). We will document the reasons for voluntary or involuntary withdrawal from study procedures, if known.

18. Risks to Participants

The potential risks from data collection procedures used in this study are minimal. Most data are obtained by interviewer-administered surveys, observation, or non-invasive procedures and pose no risk of physical harm to the participant or other household members. Study procedures have no known risks to an embryo or fetus should the subject be or become pregnant.

There may be a risk of a breach of privacy in community sessions, as community members will be meeting together to discuss problems and solutions they face in their communities. Should a participant not wish to answer a question or attend a session, they will be free to do so without any risks to the individual or his/her household.

As part of the biomarker and air pollution monitoring components of the study, field staff will conduct household visits. Participants may view these visits as inconvenient or an invasion of privacy.

Other potential risks of the biomarker/air pollution monitoring include:
- Wearing personal air pollution monitor devices over 24 hours may be viewed as obtrusive.
- Wearing silicone wristbands for 8 days may be viewed as annoying.
- Women may feel uncomfortable providing a urine sample.
- Collecting plastic waste in their homes over a week at baseline, 4-5 months and 12-13 months may be inconvenient and viewed as messy.

The possibility of these adverse events will be minimized by:
- The air pollution monitor we will use is the smallest available device that provides reliable measurements of personal $PM_{2.5}$.
- The silicone wristbands are the same as those commonly worn as decorative accessories.
- Allowing women to collect their urine samples in the privacy of their home, and then store the samples in coolers to be retrieved by trained fieldworkers.
- Providing each home with sealed bags to store plastic trash, thus preventing collection of vermin and insects. This will also limit trash collection period to 1 week, which will avoid trash accumulation in the homes.

During the informed consent process, participants will be informed that their participation in this study is voluntary. If at any point in time, a participant should wish to discontinue his/her involvement in community sessions, they are free to do so without any penalty, prejudice, or coercion to the individual or members of his/her household.

19. Potential Benefits to Participants

Direct benefits to individual participants in intervention villages include knowledge gained from the 12-week working groups. In addition, participants in intervention villages will benefit from community-driven interventions to reduce plastic combustion and exposure. Participants in control villages will receive small trees for reforestation during the period of the intervention activities in the intervention group. The intervention activities will be shared with members of these communities, and both intervention and control villages will be provided with support to further the dissemination of interventions in these communities.

Chronic, high exposures to air pollution from plastic trash burning are pervasive in Guatemala and in other parts of the world facing similar exposures to toxic smoke. We hope to provide stronger evidence of the link between air quality and health benefits, such as reduced biomarkers of exposure to urinary metabolites of plastic combustion. This study represents a unique opportunity to develop, test, and evaluate interventions that are affordable, achievable, and sustainable in highly exposed communities. Therefore, this project has potential indirect benefit not only to those residing in Guatemala, but also other low-middle income countries similarly burdened by high exposures to air pollution and where regional and national implementation policies may be informed by our findings.

20. Compensation to Participants

<u>Formative phase and evaluation of the of the dynamic working group:</u> We will provide a small financial incentive ($3 in Guatemalan *quetzales*) for those who participate in participant observations, key informant interviews and focus groups.

<u>Biomonitoring home visits (control and intervention arms)</u>: We will provide the equivalent of $5 in Guatemalan *quetzales* at each visit to the 400 women who participate in the monitoring study to compensate for their time in the study. If a participant withdraws early, they will only receive monetary compensation that corresponds with completed visits (baseline, 4-5 and 12-13 months).

<u>Dynamic working groups (intervention arm):</u> At our first meeting, we will inform working group participants that we will provide those who participate in 80% of the community working groups a reusable canvas shopping bag to compensate for their time in the study. These participants in the working group will also be entered into a raffle for three large prizes equivalent to $25 in Guatemalan *quetzales* at the end of the 12-week meetings in each intervention village. This compensation package was piloted in the pilot study for this proposal and was well received among the working group participants.

Intervention arm villages: Towards the end of the working groups, based on what they have learned, each intervention village will choose an activity or project to roll-out in their village. This activity will be led by the community *promotoras*. The village will be provided with materials and/or necessary equipment to carry out the activities in an efficient way, based on their chosen activity; for example, drums and gloves for recycling, or materials for soap making or composting. The materials may be of up to $1,000 in value.

Promotoras: They will receive a loan of a phone, SIM card and communication plan to coordinate activities with participants and study staff. They will also receive a small stipend for monthly activities of up to $20/month.

Control arm villages: In order to maintain retention in and engagement with the study, the community leaders of the eight control villages will receive compensation equivalent to $250 in small trees for local forestation. Community leaders will choose which community areas can be reforested, and they will organize the activities with study staff. This activity will be coordinated with the Auxiliary Board and COCODES of each community. Study staff will do a monitoring activity, preferably by phone, at 3 and 6 months with the community leaders to provide attention to the control group.

Control village activity and dissemination meeting materials: At the end of the entire trial (second half of 2026), the field team and the *promotoras* from the intervention communities will make a presentation of the results obtained (the 'product') in various intervention communities to the control communities (including evaluation of successes and barriers). The presentations will have a 'festive' character and will be part of a community party. Community leaders identified in the community assessment and participants from the control group will be invited. The communities will be given $200 for each meeting/party. The communities will then receive compensation equivalent to $500 for them to choose which activity they want to implement.

21. Data Analysis, Management and Confidentiality

SPECIMEN ANALYSIS

Air pollution laboratory analysis

Particulate Matter ($PM_{2.5}$)**:** Before and after sample collection, PTFE filters will be conditioned and weighed at the UVG filter weighing lab. $PM_{2.5}$ mass will be calculated as the difference between pre- and post-sampling PTFE filter weights, each determined in duplicate. Corrections will be made for buoyancy based on temperature and atmospheric pressure. $PM_{2.5}$ masses will then be converted to mass concentrations by dividing by the sampled air volume. Mean change in field blank filter masses (5% of samples will have field blanks) will be subtracted. We will measure precision by performing duplicates in 5% of samples.

Black carbon: At the UVG lab, we will analyze 1,200 filters to determine the optical attenuation and black and brown carbon mass. The analysis is non-contact, non-contaminating and non-destructive. Therefore, filters can be subsequently analyzed for PAHs/VOCs and metals (see below). The analysis method requires no support gases or consumables.

Additional air pollution analysis in a subset

Metals/Elements: A subset of 600 37-mm PTFE filters will be sent to Colorado State University for analysis of trace elements in particulate matter during the formative phase to assess for viability for the Main Trial.

PAHs: A subset of 120 37-mm quartz filters will be analyzed at the University of Iowa to assess the composition and mass of TPB and 25 PAHs, as done in previous studies. The remaining quartz filters will be stored for future analysis when funding is available.

Urine biomarkers laboratory

In addition to urinary Sb, target analytes are outlined in **Table 5**. We will attempt to identify unknown metabolites in untargeted suspect screening.

PAHs and VOCs: Eight PAH and six VOC analytes will be targeted. 1.0 ml (PAHs) and 0.5 ml (VOCs) of urine will be aliquoted and spiked with isotopically labeled internal standards for automatic recovery correction and normalization of mass spectral data.

Phthalates and Bisphenols: Nine phthalate and two bisphenol analytes will be targeted. A 1.0 ml aliquot of urine spiked with isotopically labeled analogues of target phthalates and phenols will be subjected to enzyme hydrolysis to liberate glucuronide-bound conjugates.

**Table 5. Target Analytes**	**Parent Toxicant**
Phthalates	
Mono-ethyl phthalate	Diethyl phthalate, Benzylbutyl phthalate
Mono-n-butyl phthalate	Dibutyl phthalate
Mono-i-butyl phthalate	Dibutyl phthalate
Mono-benzyl phthalate	Benzylbutyl phthalate
Mono-2-ethylhexyl phthalate	Di-2-ethylhexyl phthalate
Mono (2-ethyl-5-oxohexyl) phthalate	Di-2-ethylhexyl phthalate
Mono (2-ethyl-5-hydroxyhexyl) phthalate	Di-2-ethylhexyl phthalate
Mono (2-ethyl-5-carboxypentyl) phthalate	Di-2-ethylhexyl phthalate
Mono (2-carboxymethylhexyl) phthalate	Di-2-ethylhexyl phthalate
Bisphenols	
Bisphenol A	BPA
Bisphenol S	BPS
Polycyclic Aromatic Hydrocarbons (PAHs)	
1-naphthol	Naphthalene
2-naphthol	Naphthalene
2/3-OH-flourene	Flourene
1-OH-phenanthrene	Phenanthrene
2-OH-phenanthrene	Phenanthrene
3-OH-phenanthrene	Phenanthrene
4-OH-phenanthrene	Phenanthrene
1-OH-pyrene	Pyrene
Volatile Organic Compounds	
N-acetyl-S-(2-hydroxy) cysteine	Acrylonitril, Vinyl chloride, Ethylene oxide
N-acetyl-S-(1-phenyl-2-hydroxy)-L-cysteine	Styrene
N-acetyl-S-(benzyl)-L-cysteine	Toluene
N-acetyl-S-(phenyl)-cysteine	Benzene
N-acetyl-S-(n-propyl)-L-cysteine	1-bromopropane
N-acetyl-S-(3-hydroxypropyl)-L-cysteine	Acrolein

Silicone wristband laboratory analysis (metabolites will be analyzed under a separate protocol; funding is pending for the lab analysis but is briefly described here)

Wristbands will be processed and analyzed according to previously published procedures (Wise et al., 2020). In brief, each wristband will be cut into similarly-sized pieces, weighed, placed in a glass tube, and spiked with extraction surrogates. They will then be extracted in 10 mL of a 50:50 (v/v) mixture of hexane/dichloromethane in a 15 min sonication extraction, 3 times. The extracts will be concentrated, cleaned, then reconstituted. A suite of isotopically labeled recovery standards will be spiked into each sample prior to mass spectrometry analysis. The samples will be analyzed for a suite of target compounds using a Q Exactive GC hybrid quadrupole-Orbitrap GC−MS/MS system (Thermo Scientific) operated in full scan in the full scan electron ionization mode. The samples will be analyzed for BFRs using a single-quadrupole GC−MS (Agilent 6890N and 5975, respectively) operated in the negative chemical ionization mode (Anderson et al., 2017; O'Connell et al., 2014). Field blanks and lab blanks will be processed and analyzed with each batch of wristbands for quality control and quality assurance.

DATA ANALYSIS

STATISTICAL METHODS FOR AIM 1 (Urinary metabolites):

Baseline assessment and working group refinement surveys will be analyzed using descriptive statistics.

Key informant interviews will be audio-recorded and transcribed. Transcripts, notes from participant observations, and ethnographic field notes will be coded and analyzed using nVIVO software. We will use thematic analysis, which is flexible and theoretical and can be applied across a range of qualitative methodologies. We will use both inductive and deductive approaches, looking at the semantic content and patterns in the data. Open coding ends when core categories explain specific behaviors. Categories (i.e., themes or variables) and their properties (sub-categories) will be tested deductively based on the constructs of the BCW (Table 1). New themes will be identified and coded using an inductive thematic approach. We will use this approach to identify important topics and intervention strategies to be covered in the working groups.

Mixed methodologies offer causal explanations grounded in different kinds of empirical data. Informant-driven qualitative findings, such as focus group responses post-intervention, will be: 1) assessed against demographic characteristics of working group participants; 2) linked to the reach, effectiveness, adoption, implementation fidelity, and sustainability of intervention strategies (Aim 1); and 3) compared to quantitative findings from air pollution exposures and biomarkers (Aim 2). Thus, mixing methods may contextualize both the depth (qualitative) and breadth (quantitative) of patterns of plastic burning and changes related to the intervention strategies (Tahakkori A and Teddlie C, 2003).

***Sample size and study power**:* We estimate that at least 200 women will attend working groups and follow-up at 12-13 months and that 200 women will follow-up at 12-13 months in control

villages. We will ask both groups at baseline and 12-13 months "do you burn plastic waste in your home?". We assume that in control households there will be a 5% decrease and that in intervention households there will be an approximately 25-30% decrease in burning trash over time. Given these expected differences between the two groups, a small-to-moderate effect size (Cohen's w=0.14-0.16) will be detected at 80% power. This effect size translates to detecting a difference of 24-27% between the two groups (a 5% decrease for control group versus a 29-32% decrease for the intervention group).

<u>*STATISTICAL METHODS FOR AIM 2:*</u>

Urinary biomarkers and air pollution:
Urinary biomarker and air pollution concentrations are typically right-skewed and will be described as the geometric mean (95% confidence interval). We will use quantile regression to compare distributions between and within groups. For urinary data, we will control for dietary intake (ingestion route) or cosmetic product use (dermal route) to isolate the effects of air pollution exposures on urinary results. We will estimate variance components to assess intraclass correlations among repeated measures within subject and village. For silicone wristband data and urinary biomarker data, we will replace values below limit of detection (LOD) with LOD values.

Analysis Methods:
We will log transform our data and use linear mixed effects models. Multi-level mixed effects models (MLM) with random (individual- and village-level) and fixed (treatment group, study period, and group-by-period interaction) effects will be employed to test for trajectories of change for intervention group versus control group focusing on group-by-time interaction effect. If there are significant differences between the 2 groups at baseline (e.g., difference in ambient air pollution), these will be controlled by design and estimated by the main effect for study group.

MLM longitudinal models evaluate the changes across the three time points, and interaction of group and each separate follow-up time point will allow planned post hoc estimates of both the immediate intervention effects (4 months) and the longer term sustained effects (12-13 months).

We will use the Stepdown-minP procedure to calculate p-values that take into account multiple comparisons and the correlation expected among multiple measures of air and urine concentrations. All model assumptions will be tested with standard diagnostic tests and influence statistics used to test the distributions of the residuals. MLM utilize all available data for all participants at each time point.

For compounds isolated from the silicone wristbands, we will create a regression tree for each compound (the dependent variable. Log transformed) and items from the questionnaires (dermal and inhalation sources) as the independent variables. Model performance will be evaluated using RMSE (root mean square errors) in order to understand which of the variables have more explanatory power (McLarnan et al., 2024).

Missing Data:
From previous experience measuring personal air pollution exposures longitudinally in rural Guatemala, we expect about 85% completeness accounting for loss to follow-up and intermittent

missingness due to logistic, communication and technological failures (Smith et al., 2010). We will reduce missing data by programming and distributing an automated visit calendar based on the study schedule that is evaluated for completion on a weekly basis.

We will use the doubly robust estimation method to address bias due to missing data. First, we will explore models to predict missing exposures, including predictors such as baseline characteristics, previous exposure measures, and reported and observed behaviors related to exposure sources. If this model has predictive validity, we will use it in a multiple imputation sensitivity analysis.

Our second approach will be to build a model that explains missingness by modelling the probability of missingness conditional on treatment assignment and baseline covariates, as in inverse probability weighting. Finally, if both models are found to be predictive, we will apply the doubly robust estimation for targeted inference, which relies on parameters from either of these models being estimated consistently. R software will be used.

Sample size and study power*:* The final sample size is estimated to be between 300 to 400 women (150 to 200 in each group). With the lower bound sample size of 300 (150 in each group), we will be powered to detect small-to-moderate effect sizes on urinary metabolite differences (Cohen's f=0.20-0.25) for the group, time and group-by-time effects in a repeated measures analysis of variance for 2 groups and 3 time points. It is expected that the lower bound sample size of 300 (150 in each group) will account for both attrition and intermittent missing data as well as the potential for non-independence of households clustered within villages, which will be measured by intraclass-correlation (ICC).

STATISTICAL METHODS FOR AIM 3:

We will measure the change in plastic waste weight across two time points for households in the working groups, as well as differences between control and intervention households. We will estimate kg/person/day of plastic waste and adjust for household and socioeconomic indicators that may explain patterns of plastic waste.

Emission factors:

We will estimate emission factors (EFs) for various chemical species from plastic burning, using the air pollution exposure data from Aim 2 (120 filters collected for elements/PAHs at baseline and 4-5 months) and the ambient monitoring in each village.

We will use the carbon mass balance approach to determine fuel-based EFs for gases, in units of mass of pollutant per kilogram of fuel burned (g per kg^{-1}). We will apportion $PM_{2.5}$ concentrations on PTFE and quartz filters to plastic burning, using EPA's chemical mass balance model (EPA-CMB) version 8.2.

Source profiles for garbage burning and plastic burning will be drawn from the NAMaSTE field campaign. We will use separate source profiles for open biomass burning and biomass-powered traditional cooking stoves to make sure that we quantify exposure from plastic burning. We will use the existing plastic-burning $PM_{2.5}$ EF of 84 ± 13 g kg-1 and derive particle-bound PAHs and metals by using each component's mass fraction in $PM_{2.5}$, based on the source apportionment.

Emissions estimates:
Using the newly-estimated plastic burning EF for various species, we will create emissions estimates for plastic burning in the 16 villages. Combining the estimated EFs and plastic waste estimates from 400 households, we will quantify emissions of observed BC, PAHs, VOCs, and elements in 16 villages.

We will use the following equation: $E_i = EF_i \times M$, where Ei is the emissions due to plastic incineration for species i (g), EFi is the EF for species i (g per kg^{-1}), and M is the amount of plastic burned (kg), as was done in our previous work.

We will produce emissions estimates for each of the observed BC, PAHs and elements, with their mean EF and SD values, in addition to utilizing the above-mentioned $PM_{2.5}$ EF and 1 million Monte Carlo samplings. The Monte Carlo sampling methodology will be similar to our emissions estimate methodology in previous studies.

We will first create a distribution of EFs for each pollutant and the amount of plastic burned per household, based on the mean and SD from observational data. We will randomly draw the EF and the amount of plastic waste from the two distributions, respectively, 1 million times to create a new distribution of emissions estimates from plastic burning per species of interest. Using the most recent 2018 census data and the amount of garbage, as well as the percentage of plastic waste from the recent World Bank database, we will create emissions estimates, again using the 1 million Monte Carlo samplings. Due to data availability for Guatemala, in addition to Jalapa, we have city-level emissions estimates for Guatemala City, Antigua, and Jutiapa. For other parts of Guatemala, we will use country level estimates. These emissions estimates will then be spatially distributed across Guatemala, following the garbage burning emissions estimates distribution in a pilot study.

Emissions scenarios:
For forecasting future emissions from plastic burning, we will create four emissions scenarios for the year 2030. Based on differences in plastic waste collected in 400 households, we will estimate the mitigation potential for different chemical species of interest from reduced plastic waste in Jalapa, Guatemala.

We will create four scenarios: 1) a business-as-usual scenario, where plastic waste per capita stays the same; 2) a moderate scenario, where plastic waste per capita changed over time in the working group participant group is scaled up to all Jalapa residents; 3) Jalapa plastic ban scenario, where there is no plastic waste in Jalapa; and 4) Guatemala plastic ban scenario, where there is no plastic waste in Guatemala.

Modeling:
We will use the "online" Weather Research and Forecasting (WRF) model coupled with Chemistry version 3.7.1 (WRF-Chem) to simulate the regional air quality over Guatemala and Central America to assess the impacts of different plastic emissions on air quality at the local and regional level. In this project, we will also model PAHs, using the PAH extension that is available.

The model domain will cover most of the Central America region with 120×100 grid cells, using a Mercator map projection. The horizontal grid spacing of the largest domain will be 27km×27km

and it will encompass all of Central America. We will have two nested domains, the one in the middle for Guatemala (9km x 9km horizontal grid spacing) and the inner most domain for Jalapa (3km x 3km horizontal grid spacing). Our model will have 31 vertical levels from the surface (~10,000Pa) to 5,000Pa. The center of the domain will be on Wasala, Nicaragua at 13° latitude and -85° longitude.

In this study, we will use the Regional Acid Deposition version 2 (RADM2) atmospheric chemical mechanism for gas-phase chemistry. For aerosol chemistry including some aqueous reactions, we will use the Model Aerosol Dynamics for Europe with the Secondary Organic Aerosol Model (MADE/SORGAM). With this model set up, we will predict the mass of seven aerosol species, including sulfate, ammonium, nitrate, sea salt, BC, OC and secondary organic aerosols, in addition to PM2.5, as done in our previous work.

Model-simulated horizontal winds, temperature, and moisture at all vertical levels will be nudged to the large-scale meteorological fields from the National Center for Environmental Prediction Global Forecast System final gridded analysis datasets. The initial and lateral boundary conditions for chemical species will be taken from the Model for OZone and Related chemical Tracers (MOZART) version 4 (Emmons et al., 2010). The MOZART model has 28 vertical levels from the surface to ~100Pa and it has horizontal resolution of 2.8 degrees latitude.

For the present situation, we will conduct two model simulations. One will be with our newly gridded plastic emissions estimates added to other anthropogenic and natural emissions sources, while the other will be without the plastic emissions but including all other sources.

For 2030, we will conduct simulations based on four different plastic burning emissions scenarios, in addition to all other anthropogenic and natural emissions that stay the same among the four scenarios. The purpose of the future simulations will be to assess the impact of the potential reduction of plastic burning on local and regional air quality. For each scenario, we will use WRF-Chem to model air quality for a year, conducting 12 monthly simulations. For each year-long simulation, the model will be spun-up for 14 days to allow the model to ventilate the regional domain and this period will not be included in the analysis. For our baseline simulation for the year 2021, we will evaluate our WRF-Chem model results by comparing simulated pollutant concentrations with observational data available in Guatemala.

For anthropogenic emissions, we will use the Hemispheric Transport of Air Pollution (HTAP) version 2.2. The HTAP inventory includes emissions from fossil fuel combustion and production, power transmission and distribution, transport, residential sector, industrial processes, solvents and other product use, agriculture, large scale biomass burning, waste and miscellaneous sources. Biomass burning emissions will be taken from the Fire Inventory from NCAR, which includes daily fire emissions (i.e., wildfire, agricultural fires, and prescribed burning) of CO, nitrogen oxides (NO_x), and VOCs. For biogenic emissions of CO, NO_x, methane, and 13 other chemical species, we will use the Model of Emissions of Gases and Aerosols from Nature version 2.1.

Sampling Size: For the model-observations comparison, we will use data sets that vary by sample size. We will compare our hourly, daily, and monthly average simulations with existing observational data.

Quality Assurance: All data will be continuously reviewed and monitored to ensure quality and minimize missing data, including descriptive statistics to check for any data entry errors and validation. Preliminary analyses will examine baseline differences in demographic/household characteristics and exposures by randomized groups as a randomization check.

22. Provisions to Monitor the Data to Ensure the Safety of Participants

PROTECTION OF CONFIDENTIALITY:

The study will employ standard methods for protecting the confidentiality of participants through the use of personal identification numbers (PINs) on all study materials, password-protected computer data files, and locked file cabinets for storing hard copies of interview and other study materials (e.g., signed consent forms) in Jalapa where they will then be transferred to UVG at the end of the data collection period. During the study period, the custodian of the printed consent forms will be the administrative assistant working together with the field project manager. All hard copies of study materials will be destroyed 5 years after the conclusion of the study. All study investigators will undergo training in Human Subjects Research Ethics. All key personnel will be CITI (Collaborative Institutional Training Initiative) certified per Emory IRB requirements. This training includes procedures to minimize the potential for breaches of confidentiality, including but not limited to ensuring that all files are closed, that interviews are conducted in private settings, and that no conversations about individual study participants occur in public settings.

The study will be implemented, and data collected, in a secure manner following Standard Operating Procedures (SOPs). Study subjects will be assured that information gathered during interviews and health and exposure assessments is kept confidential. Names and other easily recognizable identifiers will be removed from all questionnaires prior to data entry. No personal information (e.g., name, address, telephone number) will leave the local project office. Numeric study identifiers are included so that data from the several instruments can be linked; however, these are not meaningful to casual observers without access to the original study logs. The same procedures are followed for all biological samples.

If there is a security breach, those affected by the breach will be notified in a timely manner. The notification will be in writing and will include a brief description of the breach, a description of the types of information involved in the breach, the steps the affected individuals should take to protect themselves from potential harm, a brief description of what the trial is doing to investigate the breach, mitigate the harm, and prevent further breaches, as well as contact information.

PROCEDURES TO MONITOR PARTICIPANTS SAFETY AND MINIMIZE RISKS:

Institutional Review Boards (IRBs):

The IRB at Emory University will work with the IRB at Universidad del Valle de Guatemala (UVG) to ensure that Human Subjects Research approvals are obtained prior to the enrollment of participants. The study protocol will be reviewed by IRBs at Emory University and UVG. UCSF and UGA will rely on Emory's IRB. All study documents including approval letters will be made

available to study investigators via a secure web-based document-sharing platform (e.g., OneDrive).

The team of investigators at Emory and UVG have conducted research together for nearly 20 years, including the seminal NIH-funded RESPIRE trial, which was the first randomized trial to examine the impact of an improved biomass stove intervention on infant pneumonia. The study team has collaborated on studies evaluating maternal and early infant personal exposures to air pollution and birth outcomes, including preterm birth and infant neurodevelopmental impairment; and recently implemented an LPG stove and behavioral change intervention in pregnant women in Guatemala.

Data and Safety Monitoring Board (DSMB)

This study will not employ a Data and Safety Monitoring Board because none of the study activities (e.g., participating in community workshops, collecting urine specimens, and wearing air pollution monitors) are deemed harmful to study participants.

Data and Safety Monitoring Plan (DSMP)

The PIs will be informed of AEs/SAEs as soon as they occur and will notify the respective IRBs and NIH quarterly.

Content of Data and Safety Monitoring Report:

The investigators will also report the following to NIHI: 1) IRB-approved revisions to the study protocol, particularly those that indicate a change in risk for participants; 2) IRB-approved revisions to study consent forms; 3) Notice of any actions taken by the IRB or regulatory bodies regarding the research and any responses to those actions.

Adverse Event (AE) and Serious Adverse Event (SAE) Collection and Reporting

We do not anticipate that there will be adverse events (AEs) and serious adverse events (SAEs) for this study, which does not involve invasive procedures or health outcome monitoring, other than urinary biomarkers of exposure to combustion of solid fuels and plastics.

To assess for unexpected risks that may be associated with the study protocol, participants will be monitored for adverse events. The field project manager at the research site will perform the safety reviews. This data will be reviewed and reported to the Principal Investigator and co-investigators at Emory and UVG every week.

If important and unexpected adverse experiences occur, they will be recorded as an adverse event and will be reported to the Emory IRB and to local Universidad del Valle de Guatemala IRB per protocol and as defined according to the January 2007 Office of Human Research Protections Guidance on Reviewing and Reporting Unanticipated Problems Involving Risks to Subjects or Others and Adverse Events, Office of Human Research Protections Guidance. All issues having to do with patient safety will be reported to the Emory and UVG IRB within 10 working days for AE and within 2 days for SAEs.

Specifically, the following will be reported, in writing: 1) all serious adverse events associated with the study procedures, and/or 2) any incidents or problems involving the conduct of the study

or participation, including problems with the recruitment and/or consent processes. The Principal Investigator will provide a discussion of any side effects or problems noticed during each year in the course of the study to the IRBs on an annual basis.

DATA QUALITY CONTROL:

The Dual-PIs at Emory and the co-investigators at UVG, UGA and UCSF will oversee all training of personnel. To ensure data quality, standard operating procedures have been developed for each survey instrument and air monitoring procedure in English and Spanish and will be modified for the present study. All consents and questionnaires will be translated into Spanish, back-translated into English to confirm consistency with original questions.

The quality of the study data will be considered at each stage of design, training, implementation, and analysis. Forms will be designed such that data collection will be accurate, complete, and standardized, and efficient for data recording, processing, and analysis. All forms and study protocols will be pilot tested. Forms will be assigned a version number, which will be updated if changes to the form are required over the course of the study. Before the revisions are released, the data management system will be updated to accept these revisions.

Participant identifiers will be assigned in a way to improve tracking and monitoring of study activities (such as embedding the recruitment source and panel number within the identification number). Staff will be required to use pre-printed QR-coded ID labels to avoid transcription errors when labeling study samples.

The timing of follow-up interviews, and acceptable 'windows' during which visits can still be completed will be specified. Because some participants may not complete each interview, and not all interviews will be completed on the scheduled date, expected dates for visits will be scheduled according to the baseline date rather than from an interval from the previous visit.

Interviewer training will include in-person techniques, the importance of standardization, coding, correcting errors, study protocols, and data entry/management. Supervision will be ongoing and a schedule for booster-training and performance will be implemented.

A comprehensive Standard Operating Procedures manual will be developed and copies will be stored and updated at each location where data are to be collected or entered. Detailed protocols for each procedure will be assigned a version number and date. During site visits, manuals will be reviewed to verify that the most up-to-date procedures have been implemented.

Protocol monitoring will be performed at UVG and at Emory. Reports will provide summaries such as the number of visits completed, exceptions to the protocol (visits outside of window, missed visits, missing forms or samples), and dates of expected visits. Summaries on the time to data entry, time until errors are resolved, and completeness of forms will be provided. Running these reports will assist the field project manager and the project coordinator in identifying problem areas for quick remediation.

An essential element of this protocol is open, frequent and accurate communication between Drs. Thompson and Saikawa at Emory and co-Investigators and field staff at UVG, UGA and UCSF, which is facilitated by good cell phone and internet links at the project headquarters. Every effort

will be made to ensure that the research staff is well informed of the study goals, procedures, and progress. By feeling invested in the project, they will remain motivated to collect the most complete and accurate data and will be more forthcoming about problems and concerns about the data collection process. Whenever possible, the staff will be included in decisions regarding protocol changes and scheduling and will be encouraged to make suggestions based on their experiences. Weekly conference calls with project staff will be conducted. In-person visits by investigators to the study center will be conducted at least quarterly.

Data Quality Control for Exposure Assessment and Urinary Biomarkers:

Considerable attention will be paid to QA at every stage of the monitoring, from lab analysis; specimen handling and calibration at the field headquarters; and protection of instruments in transport. Protocols will be kept in both English and Spanish. Questionnaires and monitoring forms will be checked nightly by field staff and corrected, repeated, or rejected according to protocols. Email reports and weekly conference calls will be conducted to discuss and prevent problems.

23. Provisions to Protect the Privacy Interest of Participants

DATA MANAGEMENT:

Data management will be overseen in Guatemala by the project manager and the data manger. Extensive data management protocols will be modified and applied to the current study. Trainings will be held on at least a quarterly basis in order to ensure that the field team and other staff are following study procedures, including recruitment of participants and informed consent, and to identify any problems with procedures or protocols. All information about participants is coded using unique identification numbers that link questionnaires. All data are kept in locked filing cabinets or password protected computer files. Measures to guarantee security and confidentiality of data, including back-up to mass storage media, off-site fireproof storage and data encryption, will be implemented and strictly enforced.

Data Entry and Quality Control:

The database will include data from: 1) baseline surveys, 2) follow-up surveys, 3) urine biomarker forms, and 6) air pollution forms. Chain-of-custody procedures will be developed for each specimen type. Data forms will be designed such that data collection will be accurate, standardized, and efficient for data recording, processing, and analysis. Prior to the start of the study, all forms and protocols will be pilot tested. Participant identifiers will be stripped of protected health information (PHI) and will be assigned in a way to improve tracking and monitoring of study activities (e.g. embedding recruitment source and panel number within the identification number). We will use Research Electronic Data Capture (REDCap), a secure, web-based application for building and managing databases at no cost to Emory researchers, and can be shared with approved institutions, such as designated UVG, UGA and UCSF researchers. Data dictionaries, survey forms and databases will be created in the REDCap web portal for quantitative data. All survey data will be entered onto data-encrypted tablets. Bimonthly reports on the completeness, accuracy and timeliness of data entry will be reviewed by the Dual-PIs. Descriptive analyses (frequencies, cross-tabulations, between-item consistencies, etc.) will be

run to look for systematic and random errors. Data reports will be generated by Emory and distributed to UVG, UGA and UCSF. R software will be used for data analysis; all changes resulting from cleaning will be documented.

Data Security:

Each REDCap user will be assigned their own user account. Access to projects will be assigned by the PI at Emory. For instance, certain users may be allowed to enter or modify data, but not export data. All information about participants will be coded using unique identification numbers that link survey forms and stored on password-protected computers. Every week an automatic back-up of data and project materials will take place: the field project manager will make a copy of the data to be stored on Emory OneDrive, a secure cloud-based storage system. Access to data files is strictly monitored by the project manager. All requests for data use are approved by the Dual-PIs at Emory and the co-investigators at UVG, UGA and UCSF.

CONFIDENTIALITY:

We will use several methods, which have proven to be successful in previous studies, to ensure confidentiality.

All local fieldworkers who will collect data from study participants will receive training in the ethical conduct of research in Spanish through CITI before initiating recruitment and data collection. This program provides instruction pertaining to confidentiality including not discussing information about study participants or study results in public or with any non-project personnel. We will ask all field personnel to sign a confidentiality statement (see Appendix B). Participant names will not appear in study reports or publications; instead, we will use de-identified study codes, which will be used to link data files. The consent form will include a section on photographs and audiotapes, clearly explaining that these are voluntary. Study participants will elect, via a separate consent form, whether or not they agree to be photographed and/or audiotaped prior to initiation of the study.

The potential risk of a breach in privacy in community sessions will be mitigated by clearly explaining to all community members that their participation in these sessions is voluntary. All participants may decline to attend a session or answer a question without any risks to the individual or his/her household.

In the biomonitoring sub-sample, interviews will be conducted in the privacy of participants' homes. Acknowledging that the presence of other household members present may be viewed as a loss of privacy, we will offer all participants the option to conduct the interview alone. Even if the interviewee does not mind others listening or if the house appears to be small/crowded, we will always ask women if interviews may be conducted in areas of the home away from other household members, as we have done in other past studies. We will re-discuss confidentiality at each household visit and explain to the women that they may refuse to answer/comment on any question asked during the interviews at any point in the study period.

The risks of home visits by project field staff or *promotoras* being viewed as an invasion of privacy will be minimized by the bonds that local fieldworkers have developed with the participating communities as a result of their work in previous studies. The fieldworkers are respected leaders

in their communities and are highly competent and conscientious interviewers; without their involvement, this study would not be possible.

24. Economic Burden to Participants

There are no costs to participants.

25. Informed Consent

The Institutional Review Board (IRB) at Emory University will work with the Ethics Committee at the Universidad del Valle de Guatemala to ensure that Human Subjects/Ethics approvals are obtained prior to enrollment of participants in order that all participants are safe from harm and their rights are protected.

Written informed consent or assent will be obtained by a trained and certified Spanish-speaking local fieldworker in Spanish for all participants who wish to participate in this study. The written consent forms will be translated into Spanish and terminology used will be pre-tested and back-translated. If a participant is between 15 and 18 years of age, he or she will provide written assent, and the caregiver of the child (e.g., mother, father or adult caregiver who is the designated legal guardian) will provide consent for their child to participate in the study. Assents will be required of all participants between 15 and 18 years of age. *Promotoras* will provide consent for their additional activities via a short consent addendum.

During the consent process, the fieldworker will thoroughly describe the details of the study procedures and potential risks and benefits. The fieldworker will clearly explain to the participant that their participation in the study is voluntary and that their decision to participate or not to participate will not in any way affect their care at the community health clinics and/or hospitals. Consent to participate will be recorded on a signed consent form. Although some heads of households will not be literate, people are accustomed to signing their name, or a thumbprint as proof of consent, to a document after having it read to them. Households will be free to withdraw from the study at any time without prejudice or coercion. The consent will take approximately 20-30 minutes to administer and will be read to participants in Spanish by a trained fieldworker. Steps will be taken to ensure participants' understanding by assuring that the consent is in a simple language at an elementary-school level.

26. Setting

Identification and recruitment of participants will occur in Jalapa, Guatemala. The Jalapa Municipality has a rural population of 75,134 people in 133 villages. We will select a random subset of 16 villages from among the 29 largest villages in the municipality of Xalapán to participate in the study. All research procedures will occur in Jalapa, Guatemala.

Local scientific ethical approval will be obtained and overseen by the Ethics Committee at the Center for Health Studies at the Universidad del Valle de Guatemala in Guatemala City, Guatemala.

27. Resources Available

Feasibility of Recruiting the Required Number of Suitable Participants

We will select a random subset of 16 villages from among the 75,134 people in 29 villages in the Xalapán area of Jalapa Guatemala. We have worked in Jalapa Guatemala over the past 5 years, recruiting 800 participants in the HAPIN trial, with over 30 field workers and a study office with air pollution laboratory, clinical laboratory and oversite provided by researchers at the Universidad del Valle de Guatemala (UVG). We also conducted a pilot for the proposed study in Xalapán and have developed relationships with local communities and with the Ministries of Agriculture, Energy and Mines and Health, such that we have established excellent working relationships to conduct this study efficiently in this region. It takes many years to establish trust with local communities, and we will leverage the success of the HAPIN study to work on this new proposed study. We will be able to recruit the number of participants for the proposed study over five years.

Time Needed to Conduct and Complete Research

During the **Formative Phase (Year 1),** we will conduct a Baseline Assessment of plastic waste burning and waste management practices and socio-demographic determinants. After the Baseline Assessment we will determine the villages that will participate in the Main Trial.

The **Main Trial** will occur over a 4-year period. Each participant will be followed for approximately 1 year. However, recruitment for Aims 2 and 3 will be staggered.

As part of Aim 1, individuals will complete surveys at baseline (before the 12-week curriculum has begun), 4-5 months (after the 12-week curriculum has been delivered), and 12-13 months. Individual interviews and follow-up meetings with 6-8 members from each village will be conducted at months 3, 6, and 12 to assess whether intervention activities have been successful or not, as well as barriers and enablers of success.

As part of Aims 2 and 3, recruitment of the 400 women of reproductive age will be staggered and women will each be followed for approximately 1 year. We will monitor urinary biomarkers and personal exposure to fine particulate matter with collection of samples occurring at baseline, 4-5 months, and 12-13 months. Waste collection will occur at baseline, month 4-5 and month 12-13, and plastic will be separated out for quantification of plastic that would have been burned to estimate emissions that would have occurred.

Project oversight

Dr. Lisa Thompson and Dr. Eri Saikawa at Emory University are the Principal Investigators. Dr. Thompson will visit the project site for one week every 1-2 months to oversee the project. Dr. John McCracken is a co-investigator on the proposal. He will visit the project site every 2 months. Dr. Mayari Hengstermann is a co-investigator and will spend 3-6 months a year at the project site every year. Ms. Maria Renee Lopez is the local site Principal Investigator. She will visit the project site every month. The local field team will be supervised by Maria Renee Lopez. She will oversee the day-to-day activities and supervise activities to ensure that standard operating procedures will be followed. Key personnel will visit the site at least one week every month. We will also hold weekly meetings via zoom with local project staff as we have done with HAPIN to make sure that

the project is being managed adequately. Given our collective experience on research projects since 2002, we have established an excellent method of communication to conduct research and resolve issues that arise during the study procedures.

Training of Research Staff

We will provide trainings for all individuals assisting with research in order to ensure that everyone is informed about the research protocol, procedures, and field staff duties and functions.

All project staff (researchers, fieldworkers, etc.) have received training in the ethical conduct of research through UVG. They have received instruction specifically pertaining to confidentiality and will not discuss information about study participants or study results in public or with any non-project personnel.

Facilities

Working group meetings will occur in local village spaces (e.g. churches or local community centers).

For the past five years we have been conducting the HAPIN trial at this research site and currently have 10 local Guatemalan field staff who speak Spanish and are well trained to conduct field-based research.

Specimens will be stored in the project office fields. Urine samples will be shipped to the Barr Laboratory at Emory University for analysis. Air filters will be processed at our field project office, weighed at UVG, and shipped to the University of Iowa and Colorado State University for analysis.

- The research office for the proposed project is in Jalapa, Guatemala. It has a bedroom and a bathroom, an air pollution lab, a biomarkers lab, and a meeting room. It contains the following:
- Project office: Desks and Chairs, 10 computers: 3 for air pollution lab, 1 for data manager, 4 for field project staff, 1 for biomarkers laboratory and 2 printers.
- Air Pollution laboratory: clean room for filter preparation, 5 filing cabinets, refrigerator and freezer, shelving for equipment storage, 5 tables for equipment preparation and calibration.
- Biomarkers lab: 1 desk area for processing urine specimens, including centrifuge and pipetting devices, 2 refrigerators, 3 -20 freezers.
- Air pollution equipment: 1 SootScan to measure black and brown carbon on filters; 70 Lascar monitors for continuous carbon monoxide monitoring; 1 CO calibration chamber,
  1 CO tank & 1 Zero air tank; 14 Casella pumps, 27 BGI Cyclones, 8 SKC Impactors for gravimetric PM monitoring; 5 Rotameters; 2 Gilibrators; 2 DryCal Defenders 510; 11 PATS+ continuous PM monitors; 5 Ultrasonic Personal Aerosol Sampler (UPAS); 72 Early Childhood MicroPEMs (ECMs) with power chargers for continuous and filter-based $PM_{2.5}$, 42 Beacon loggers, 40 Bluetooth Beacons for measuring distance from fire/stoves, 950 Geocene DOTs for continuous stove use temperature monitoring, 141 SUMS buttons for continuous stove use temperature monitoring, (Maxim Integrated), 1 MetOne E-sampler for ambient PM monitoring, 3 laptops for downloading data in the field and processing in the office.

28. References

Anderson, K.A., Points, G.L., Donald, C.E., Dixon, H.M., Scott, R.P., Wilson, G., Tidwell, L.G., Hoffman, P.D., Herbstman, J.B., O'Connell, S.G., 2017. Preparation and performance features of wristband samplers and considerations for chemical exposure assessment. J Expo Sci Environ Epidemiol 27, 551–559. https://doi.org/10.1038/jes.2017.9

Barabad, M.L.M., Jung, W., Versoza, M.E., Lee, Y.I., Choi, K., Park, D., 2018. Characteristics of Particulate Matter and Volatile Organic Compound Emissions from the Combustion of Waste Vinyl. International journal of environmental research and public health 15. https://doi.org/10.3390/ijerph15071390

Carroll, C., Patterson, M., Wood, S., Booth, A., Rick, J., Balain, S., 2007. A conceptual framework for implementation fidelity. Implement Sci 2, 40. https://doi.org/10.1186/1748-5908-2-40

Chen, G., Gully, S.M., Eden, D., 2001. Validation of a New General Self-Efficacy Scale. Organizational Research Methods 4, 62–83. https://doi.org/10.1177/109442810141004

Clasen, T., Checkley, W., Peel, J., Balakrishnan, K., McCracken, J.P., Rosa, G., Thompson, L.M., Boyd Barr, D., Clark, M., Johnson, M., Waller, L.A., Jaacks, L.M., Steeland, K., Miranda, J.J., Chang, H., Kim, D.Y., Rosenthal, J., HAPIN investigator, 2020. Design and Rationale of the Household Air Pollution Intervention Network (HAPIN) Study: A multi-country randomized controlled trial to assess the effect of liquefied petroleum gas stove and continuous fuel distribution on household air pollution and health. Environmental Health Perspectives.

Cohen, D.J., Crabtree, B.F., Etz, R.S., Balasubramanian, B.A., Donahue, K.E., Leviton, L.C., Clark, E.C., Isaacson, N.F., Stange, K.C., Green, L.W., 2008. Fidelity versus flexibility: translating evidence-based research into practice. Am J Prev Med 35, S381-389. https://doi.org/10.1016/j.amepre.2008.08.005

Cordes, L., 2011. Igniting change: a strategy for universal adoption of clean cookstoves and fuels [WWW Document]. Global Alliance for Clean Cookstoves (GACC).

De Silva, M.J., Harpham, T., Tuan, T., Bartolini, R., Penny, M.E., Huttly, S.R., 2006. Psychometric and cognitive validation of a social capital measurement tool in Peru and Vietnam. Social Science & Medicine 62, 941–953. https://doi.org/10.1016/j.socscimed.2005.06.050

Doherty, B.T., Pearce, J.L., Anderson, K.A., Karagas, M.R., Romano, M.E., 2020. Assessment of Multipollutant Exposures During Pregnancy Using Silicone Wristbands. Front Public Health 8, 547239. https://doi.org/10.3389/fpubh.2020.547239

Emmons, L.K., Walters, S., Hess, P.G., Lamarque, J.-F., Pfister, G.G., Filmonre, D., Granier, C., Guenther, A., Kinnison, D., Laepple, T., Orlando, J., Tie, X., Gyndall, G., Wiedinmyer, C., Baughcum, S.L., Kloster, S., 2010. Description and evaluation of the Model for Ozone and Related chemical Tracers, version 4 (MOZART-4). Geoscientific Model Development 3, 43–67.

Estabrooks, P.A., Smith-Ray, R.L., Dzewaltowski, D.A., Dowdy, D., Lattimore, D., Rheaume, C., Ory, M.G., Bazzarre, T., Griffin, S.F., Wilcox, S., 2011. Sustainability of evidence-based community-based physical activity programs for older adults: lessons from Active for Life. Transl Behav Med 1, 208–215. https://doi.org/10.1007/s13142-011-0039-x

Glasgow, R.E., Harden, S.M., Gaglio, B., Rabin, B., Smith, M.L., Porter, G.C., Ory, M.G., Estabrooks, P.A., 2019. RE-AIM Planning and Evaluation Framework: Adapting to New Science and Practice With a 20-Year Review. Frontiers in Public Health 7.

Glasgow, R.E., Vogt, T.M., Boles, S.M., 1999. Evaluating the public health impact of health promotion interventions: the RE-AIM framework. Am J Public Health 89, 1322–1327. https://doi.org/10.2105/ajph.89.9.1322

Government of the Republic of Guatemala, 2019. Results of the 2018 National Census [WWW Document].

Hamzai, L., Lopez Galvez, N., Hoh, E., Dodder, N.G., Matt, G.E., Quintana, P.J., 2022. A systematic review of the use of silicone wristbands for environmental exposure assessment, with a focus on polycyclic aromatic hydrocarbons (PAHs). J Expo Sci Environ Epidemiol 32, 244–258. https://doi.org/10.1038/s41370-021-00359-9

Hennessy, C.H., Moriarty, D.G., Zack, M.M., Scherr, P.A., Brackbill, R., 1994. Measuring health-related quality of life for public health surveillance. Public Health Rep 109, 665–72.

Kechter, A., Amaro, H., Black, D.S., 2019. Reporting of Treatment Fidelity in Mindfulness-Based Intervention Trials: A Review and New Tool using NIH Behavior Change Consortium Guidelines. Mindfulness (N Y) 10, 215–233. https://doi.org/10.1007/s12671-018-0974-4

Lippman, S.A., Neilands, T.B., Leslie, H.H., Maman, S., MacPhail, C., Twine, R., Peacock, D., Kahn, K., Pettifor, A., 2016. Development, validation, and performance of a scale to measure community mobilization. Social Science & Medicine 157, 127–137. https://doi.org/10.1016/j.socscimed.2016.04.002

Martínez-Restrepo, S., Ramos-Jaimes, L., Espino, A., Valdivia, M., Yancari, J., 2017. Measuring women's economic empowerment: Critical lessons from South America.

McLarnan, S.M., Bramer, L.M., Dixon, H.M., Scott, R.P., Calero, L., Holmes, D., Gibson, E.A., Cavalier, H.M., Rohlman, D., Miller, R.L., Kincl, L., Waters, K.M., Anderson, K.A., Herbstman, J.B., 2024. Predicting personal PAH exposure using high dimensional questionnaire and wristband data. J Expo Sci Environ Epidemiol 1–9. https://doi.org/10.1038/s41370-023-00617-y

Michie, S., van Stralen, M.M., West, R., 2011. The behaviour change wheel: a new method for characterising and designing behaviour change interventions. Implement Sci 6, 42. https://doi.org/10.1186/1748-5908-6-42

Miodovnik, A., Edwards, A., Bellinger, D.C., Hauser, R., 2014. Developmental neurotoxicity of ortho-phthalate diesters: review of human and experimental evidence. Neurotoxicology 41, 112–22. https://doi.org/10.1016/j.neuro.2014.01.007

Mustieles, V., Fernández, M.F., 2020. Bisphenol A shapes children's brain and behavior: towards an integrated neurotoxicity assessment including human data. Environmental Health 19, 66. https://doi.org/10.1186/s12940-020-00620-y

O'Connell, S.G., Kincl, L.D., Anderson, K.A., 2014. Silicone Wristbands as Personal Passive Samplers. Environ. Sci. Technol. 48, 3327–3335. https://doi.org/10.1021/es405022f

Rochester, J.R., 2013. Bisphenol A and human health: a review of the literature. Reprod Toxicol 42, 132–55. https://doi.org/10.1016/j.reprotox.2013.08.008

Salgueiro-González, N., López de Alda, M.J., Muniategui-Lorenzo, S., Prada-Rodríguez, D., Barceló, D., 2015. Analysis and occurrence of endocrine-disrupting chemicals in airborne

particles. TrAC Trends in Analytical Chemistry 66, 45–52. https://doi.org/10.1016/j.trac.2014.11.006

Samon, S.M., Hammel, S.C., Stapleton, H.M., Anderson, K.A., 2022. Silicone wristbands as personal passive sampling devices: Current knowledge, recommendations for use, and future directions. Environ Int 169, 107339. https://doi.org/10.1016/j.envint.2022.107339

Smith, K.R., McCracken, J.P., Thompson, L., Edwards, R., Shields, K.N., Canuz, E., Bruce, N., 2010. Personal child and mother carbon monoxide exposures and kitchen levels: methods and results from a randomized trial of woodfired chimney cookstoves in Guatemala (RESPIRE). J Expo Sci Environ Epidemiol 20, 406–416. https://doi.org/10.1038/jes.2009.30

Tahakkori A, Teddlie C, 2003. Handbook of mixed methods in the social and behavioral sciences. SAGE, Thousand Oaks, CA.

Wacławik, M., Rodzaj, W., Wielgomas, B., 2022. Silicone Wristbands in Exposure Assessment: Analytical Considerations and Comparison with Other Approaches. Int J Environ Res Public Health 19, 1935. https://doi.org/10.3390/ijerph19041935

Wise, C.F., Hammel, S.C., Herkert, N., Ma, J., Motsinger-Reif, A., Stapleton, H.M., Breen, M., 2020. Comparative Exposure Assessment Using Silicone Passive Samplers Indicates That Domestic Dogs Are Sentinels To Support Human Health Research. Environ. Sci. Technol. 54, 7409–7419. https://doi.org/10.1021/acs.est.9b06605

World Health Organization, 2016. WHO | Household air pollution [WWW Document]. WHO.

Appendix A. Timeline and Monitoring Strategies (16 villages)

Table1 Formative Phase Timeline in Years 1-2

#YEAR OF FUNDING	1									2				
YEAR	2021				2022					2022				
EXPECTED DATE	SEP	OCT	NOV	DEC	JAN	FEB	MAR	APR	MAY	JUN	JUL	AUG	SEP	OCT
FORMATIVE PHASE (8/18/2021-5/31/2022)	FORMATIVE PHASE													
CENSUS														
FORMATIVE BASELINE SURVEY														
PILOT INTERVENTION														
PILOT 1 EXPOSURE MEASUREMENT														
PILOT OPEN FIRE EMISSIONS														
PILOT/REVISE SURVEYS IN HOUSES														
PILOT 2 EXPOSURE MEASUREMENT														

Table 2 Main Trial Timeline (B=baseline measures; M4=midline measures; M12=post intervention period measures) in Years 2-5

#YEAR OF FUNDING	Year 2								Year 3												Year 4												Year 5						
YEAR	2022			2023												2024												2025											
EXPECTED DATE	OCT	NOV	DEC	JAN	FEB	MAR	APR	MAY	JUN	JUL	AUG	SEP	OCT	NOV	DEC	JAN	FEB	MAR	APR	MAY	JUN	JUL	AUG	SEP	OCT	NOV	DEC	JAN	FEB	MAR	APR	MAY	JUN	JUL	AUG	SEP	OCT	NOV	DEC
MAIN TRIAL (10/1/2022-12/31/2025)																																							
1ST INTERVENTION VILLAGE	Baseline|25			WORKSHOP			M4|22	POST-WORKSHOP FOLLOW-UP						M12|21																									
1ST CONTROL VILLAGE		Baseline|25					M4|24								M12|25																								
2nd INTERVENTION VILLAGE				B|25			WORKSHOP			M4|23	POST-WORKSHOP FOLLOW-UP						M12|25																						
2nd CONTROL VILLAGE				B|25						M4|24							M12|25																						
3rd INTERVENTION VILLAGE								B|25		WORKSHOP			M4|25	POST-WORKSHOP FOLLOW-UP							M12|25																		
3rd CONTROL VILLAGE								B|25						M4|21							M12|25																		
4th INTERVENTION VILLAGE												B|25		WORKSHOP			M4|23	POST-WORKSHOP FOLLOW-UP							M12|25														
4th CONTROL VILLAGE												B|25				M4|24									M12|25														
5th INTERVENTION VILLAGE																B|25		WORKSHOP			M4|25	POST-WORKSHOP FOLLOW-UP						M12|25											
5th CONTROL VILLAGE																B|25					M4|25							M12|25											
6th INTERVENTION VILLAGE																			B|25		WORKSHOP			M4|25	POST-WORKSHOP FOLLOW-UP							M12|25							
6th CONTROL VILLAGE																			B|25					M4|25								M12|25							
7th INTERVENTION VILLAGE																							B|25		WORKSHOP			M4|25	POST-WORKSHOP FOLLOW-UP							M12|25			
7th CONTROL VILLAGE																							B|25					M4|25								M12|25			
8th INTERVENTION VILLAGE																										B|25		WORKSHOP			M4|25	POST-WORKSHOP FOLLOW-UP							M12|25
8th CONTROL VILLAGE																										B|25					M4|25								M12|25

Table 3 (Aims 1 and 2): Evaluation and Dissemination in Years 4 and 5

#YEAR OF FUNDING	Year 4												Year 5											
YEAR	2024							2025												2026				
EXPECTED DATE	JUN	JUL	AUG	SEP	OCT	NOV	DEC	JAN	FEB	MAR	APR	MAY	JUN	JUL	AUG	SEP	OCT	NOV	DEC	JAN	FEB	MAR	APR	MAY
EVALUATION AND DISSEMINATION (AIMS 1 & 2)																								
COMMUNITY FAIRS OF INTERVENTION STRATEGIES													COMMUNITY FAIRS AT VILLAGE/REGIONAL LEVEL											
EVALUATION OF IMPEMENTATION STRATEGIES	EVALUATION OF IMPLEMENTATION STRATEGIES (ONGOING)																							
NATIONAL SCALE-UP WITH STAKEHOLDERS										REGIONAL/NATIONAL SCALE-UP (MEETINGS IN GUATEMALA)														
PUBLICATIONS/PRESENTATIONS										PRESENTATIONS/PUBLICATIONS														

Table 4 (Aim 3) Modeling Atmospheric Emissions of Plastic Burning in Years 4 and 5

					3			4												5											
			2024												2025												2026				
	NOV	DEC	JAN	FEB	MAR	APR	MAY	JUN	JUL	AUG	SEP	OCT	NOV	DEC	JAN	FEB	MAR	APR	MAY	JUN	JUL	AUG	SEP	OCT	NOV	DEC	JAN	FEB	MAR	APR	MAY
MODELING (AIM 3)																															
REVISE PLASTIC BURNING EMISSIONS ESTIMATES						REVISE PLASTIC BURNING EMISSIONS ESTIMATES																									
CREATE EMISSIONS SCENARIOS FOR 2030							CREATE EMISSIONS SCENARIOS FOR 2030																								
RUN WRF-CHEM WITH 3 SCENARIOS													RUN WRF-CHEM WITH 3 SCENARIOS																		
PUBLICATIONS/PRESENTATIONS																	PUBLICATIONS/PRESENTATIONS														

Appendix B. Acuerdo de Confidencialidad

La **confidencialidad** es un principio ético o derecho legal que implica que el personal del estudio mantendrá en secreto toda la información relacionada con su trabajo, incluyendo información recolectada sobre un participante, a menos que el mismo autorice previamente que sea revelada. Es de suma importancia que el personal involucrado en el proyecto se comprometa a mantener la confidencialidad de toda la información que se obtenga de los pacientes, procedimientos y resultados y otras actividades relacionadas. Toda información de participantes, protocolos de trabajo, bases de datos, cuestionarios y detalles del procesamiento de muestras y todos los resultados obtenidos deberán mantenerse de forma confidencial.

Como usuario de la información del Proyecto de Plásticos, usted puede desarrollar, usar, o mantener:

1. Información relacionada con un paciente/participante de un estudio/proyecto,
2. Información relacionada con el personal, o
3. Información confidencial de los proyectos y terceras personas o programas relacionados con los mismos.

Esta información puede provenir de cualquier fuente y en cualquier forma incluyendo, pero no limitada a, escrita, oral, audiovisual, y/o electrónica. Toda información es **estrictamente confidencial**.

Es la política del Proyecto de Plásticos que todos los usuarios (p. ej. empleados, personal médico, estudiantes, voluntarios y afiliados) deben respetar y mantener la privacidad, confidencialidad y seguridad de la información. Las violaciones a esta política incluyen, pero no están limitadas a:

- Divulgar información de un participante a cualquier persona fuera del estudio, incluyendo divulgar información identificable del participante por medios electrónicos o verbales, no teniendo precaución al conversar con personal del proyecto en presencia de terceros, y otros.
- Acceder información que no corresponde a las actividades que le fueron asignadas;
- Publicar sin la autorización adecuada;
- Revelar a otra persona sus códigos de acceso o contraseñas para acceder a cualquier información confidencial electrónica (incluyendo computadoras, tabletas, etc.);
- Utilizar el código de acceso o contraseña de otra persona para acceder a información confidencial electrónica;
- Manejar mal o destruir información confidencial (incluyendo el envío de comunicaciones con información confidencial);
- Revelar información del personal del estudio o proyecto.

La violación de este acuerdo puede constituir motivo para tomar acciones correctivas de acuerdo con los procedimientos que se establezcan. El uso o publicación de información confidencial también puede violar las leyes del código civil, y puede incurrir en ciertas consecuencias legales.

Al firmar esta hoja, certifico que he leído y comprendo el Acuerdo de Confidencialidad de Proyecto de Plásticos y me comprometo a realizar mis actividades de acuerdo con los principios establecidos en los mismos, conforme apliquen.

Nombre: ___________________________________

Firma: _____________________________________

Fecha: _____________________________________
