# Supplementary material for: A village-level cluster randomized controlled implementation trial to measure the effectiveness of a behavioral intervention aiming to reduce women’s exposures to household plastic waste burning in rural Guatemala: study protocol for the Ecolectivos trial
Source: Trials. 2025 Dec 13;27:49. doi: 10.1186/s13063-025-09338-z (PMC12817428; doi:10.1186/s13063-025-09338-z)
Supplement: Supplementary file 3 — Additional file 3. Materials for community working groups: COM-B surveys, homework sheets, pre- and post-tests. English and Spanish. [file 13063_2025_9338_MOESM3_ESM.pdf]

Supplementary Materials 1

Ecolectivos Community Working Group

Worksheets / Homework for Sessions 1 - 8

Intervention participants only

At the orientation meeting, before the first working group session, participants are given a pre-test which they complete at home and return at session 1.

After each individual session (1 – 8), participants are given a reflection exercise based on the curriculum topic discussed during that session and a COM-B worksheet. Participants are asked to complete the homework with the assistance of other household members and return the homework at the following session.

At the end of session 8, participants take a post-test (duplicate of the pre-test) and return it at session 9, when the intervention proposal is discussed.

This file includes:

1. Pre-/post-test in Spanish, English
2. Homework for each session:
    a. Reflection exercise in Spanish, English
    b. COM-B worksheet in Spanish, bilingual (unformatted)

Pre-test

Encuesta Inicial / Encuesta Final

Instrucciones

Esta encuesta sobre el manejo de los desechos sólidos o basura, será leída y entregada personalmente para esclarecer dudas. No existen respuestas falsas o incorrectas. Cada respuesta debe reflejar las percepciones, experiencias y conocimientos del participante. Hay **preguntas cerradas**, en las cuales se ofrecen respuestas opcionales, y **preguntas abiertas,** en las cuales los participantes deben pensar y escribir sus respuestas. La respuesta "no sé" es posible. El material deberá ser entregado en la primera sesión. De no hacerlo, no se tomará en cuenta la asistencia para el primer módulo. Tiempo aproximado de trabajo **30 minutos.**

1. ¿Qué significa para usted un "buen manejo de los desechos sólidos"?

2. ¿Qué hacen con la basura en su casa? (Puede elegir más de una opción)

- Quemo toda
- Quemo alguna, por ejemplo:
- Entierro todo
- Entierro alguna, por ejemplo:
- La recoge un servicio de basura
- Separo alguna, por ejemplo:
- Guardo alguna, por ejemplo:

Otro:

3. ¿Qué basura es la que más ve tirada en el patio o alrededor de su casa?

4. ¿Qué basura es la que más ve tirada en las calles de su comunidad?

5. ¿Cuáles son los mayores problemas para poder hacer un buen manejo de los desechos sólidos?

6. ¿Cuál cree es la mejor forma de deshacerse de la basura? (Marcar solamente una opción)

- Quemar toda
- Quemar alguna, por ejemplo:
- Enterrar todo
- Enterrar alguna, por ejemplo:
- Que la recoja un servicio de basura
- Separar alguna, por ejemplo:
- Guardar alguna, por ejemplo:
- Otra:

7. ¿Qué tipo de problemas se dan si no se quema la basura?

- Provoca enfermedades como:
- Huele mal
- Hace que los lugares se vean mal
- Provoca que se encuentren insectos y roedores (como cucarachas, ratas, ratones)
- Otro:

8. ¿Qué entiende por "contaminación ambiental"?

9. ¿Qué consecuencias tiene la "contaminación ambiental" para su salud?

10. ¿De qué manera cree que usted y su familia contribuyen al problema de la basura plástica?

11. ¿Cómo cree que el problema de la basura plástica le afecta a usted y su familia?

12. ¿De qué manera cree que el problema de la basura plástica afecta a su comunidad?

13. ¿Cómo cree que la quema de basura plástica afecta al medio ambiente?

14. ¿Cree que usted podría reducir el problema de la basura plástica?

- SÍ ¿Cómo?
- NO ¿Por qué?

15. ¿Cuál es la mejor forma para evitar que la basura plástica llegue a los ríos, lagos y océanos?

16. ¿Qué se puede hacer para que las personas participen en actividades para solucionar el problema de plásticos en su hogar y comunidad?

17. ¿Qué se puede hacer para crear conciencia ambiental sobre el problema de plásticos en su hogar y comunidad?

Initial / Final Survey

Instructions

This survey on solid waste or garbage management will be read for clarification and handed out in person. There are no incorrect answers. Each response should reflect the participant's perceptions, experiences and knowledge. There are **closed questions,** in which optional answers are offered, and **open questions,** in which participants must think and write their answers. The answer ***I don't know*** is possible. The material must be handed in at the first session. If not, attendance will not be taken into account for the first module. Approximate time to complete: **30 minutes.**

1. What does ***good management of solid waste*** mean to you?

2. What do you do with the garbage in your house? (You can choose more than one option)

- I burn it all
- I burn some of it, for example:
- I bury it all
- I bury some of it, for example:
- It is picked up by a garbage service
- I separate some of it, for example:
- I keep some of it, for example:

Other:

3. What type of garbage do you see littered the most in the yard or around your home?

4. What type of garbage do you see littered the most on the streets in your community?

5. What are the biggest problems for good solid waste management?

6. What do you think is the best way to dispose of garbage?

- Burn it all
- Burn some of it, for example:
- Bury it all
- Bury some of it, for example:
- Have it picked up by a garbage service
- Separate some of it, for example:
- Save some of it, for example:
- Other:

7. What kind of problems occur if garbage is not burned?

- Causes diseases such as:
- Smells bad
- Makes places look bad
- Attracts insects and rodents (such as cockroaches, rats, mice)
- Other:

8. What does the phrase ***environmental pollution*** mean to you?

9. What are the consequences of "environmental pollution" for your health?

10. In what ways do you think you and your family contribute to the plastic waste problem?

11. How do you think the plastic waste problem affects you and your family?

12. How do you think the plastic waste problem affects your community?

13. How do you think the burning of plastic waste affects the environment?

14. Do you think you could reduce the problem of plastic waste?

- YES How?
- NO Why?

15. What is the best way to prevent plastic waste from reaching rivers, lakes and oceans?

16. What can be done to get people **involved in activities** to solve the plastics problem in their homes and communities?

17. What can you do to **spread environmental awareness** about the plastics problem in your home and community?

Session 1

Generation of Solid Waste and Garbage

Theme: Identification of the main problems of solid waste management, including sources of contamination in communities

Guía de Trabajo en Casa (Ejercicio de Reflexión)

MÓDULO #1: GENERACIÓN de desechos sólidos o BASURA

Muchas veces, no nos damos cuenta de un problema hasta que lo hacemos visible. Por ejemplo, ¿te has dado cuenta, qué cantidad de cosas que utilizas diariamente, vienen en recipientes plásticos o están hechas de este material? En la siguiente lista escribe las cosas que ves en tu casa, las cuales están hechas de plástico o vienen en contendores plásticos.

La cocina

Por ejemplo: botellas de aceite

El comedor

Por ejemplo: sillas

La sala

El dormitorio

La letrina o baño

El patio

Homework Guide (Reflection Exercise)

MODULE #1: GENERATION of solid waste or GARBAGE

Many times, we don't realize there is a problem until it becomes visible. For example, have you noticed how many of the things you use every day come in plastic containers or are made of plastic? In the following list write down the things you see in your home that are made of plastic or come in plastic containers.

Kitchen

For Example: Bottles of oil

Dining room

For Example: Chairs

Living room

Bedroom

Toilet or bathroom

Backyard

COM-B

1. ¿Cómo cree que le afecta a usted y a su familia el problema de la basura plástica?

2. ¿Cómo cree usted que afecta a su comunidad el problema de la basura plástica?

3. ¿Qué tan difícil o fácil sería para usted usar menos plástico en su vida diaria?

Difícil Fácil No sé

4. ¿Cree que usted podría ayudar a reducir el problema de los residuos plásticos?

SI NO

5. ¿Qué tan importante es para usted solucionar el problema de la basura plástica?

Muy Importante Un Poco Importante

No Tan Importante Nunca lo pensé

6. ¿Qué cree usted que es **peor** para su comunidad hacer con la basura plástica?

Tirar la basura plástica

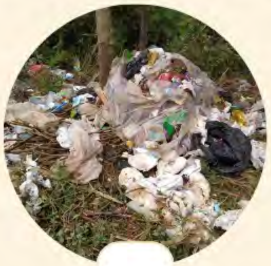

Quemar la basura plástica

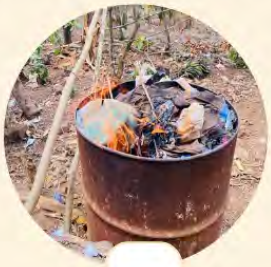

7. ¿Qué beneficios espera para usted y para su comunidad al participar en las capacitaciones?

COM-B - MÓDULO 1: Generación de desechos sólidos o basura/ Generation of solid waste or garbage

	Pregunta/Question	Respuesta/Answer
1	¿Cómo cree que le afecta a Usted y a su familia el problema de la basura plástica?<br>How do you think the problem of plastic trash affects you and your family?	[texto]
2	¿Cómo cree usted que afecta a su comunidad el problema de la basura plástica?<br>How do you think the plastic trash problem affects your community?	[texto]
3	¿Qué tan difícil o fácil seria para usted usar menos plástico en su vida diaria?<br>How difficult or easy would it be for you to use less plastic in your daily life?	1. Difícil<br>2. Fácil<br>3. No se<br>1. Difficult<br>2. Easy<br>3. I don't know
4	¿Cree que usted podría ayudar a reducir el problema de los residuos plásticos?<br>Do you think you could help reduce the problem of plastic waste?	1. Si<br>2. No<br>1. Yes<br>2. No
5	¿Qué tan importante es para usted solucionar el problema de la basura plástica en su vida, en comparación con otros tipos de basura?<br>How important is it for you to solve the problem of plastic trash in your life, compared to other types of trash?	1. Muy importante<br>2. Un poco importante<br>3. No tan importante<br>4. No se<br>1. Very important<br>2. Somewhat important<br>3. Not very important<br>4. I don't know
6	¿Qué cree usted que es peor para su comunidad: tirar o quemar la basura plástica?<br>What do you think is worse for your community: throwing away or burning plastic garbage?	• *Foto de un espacio natural cubierto de basura plástica*<br>• *Montón de basura de plástico que arde*<br>• *Photo of a natural space covered in plastic trash*<br>• *Plastic trash pile that burning*
7	¿Qué beneficios espera para usted u su comunidad al participar en las capacitaciones?<br>What benefits do you expect for yourself or your community by participating in the trainings?	[texto]

Session 2

A World of Plastic

Theme: Personal, family and community practices of plastic waste management and effects on the ecosystem

Guía de Trabajo en Casa (Ejercicio de Reflexión)

MÓDULO #2: UN MUNDO de plástico

Océanos y plásticos, ¿sabes o adivinas?

Instrucciones: Marca a la par del enunciado si crees es

VERDADERO **V** FALSO **F**

V F 1. Los océanos cubren alrededor del 70% de la superficie del planeta.

V F 2. El cambio climático y la presencia de microplásticos están amenazando la vida de los océanos.

V F 3. Cada botella de plástico tarda unos 450 años en descomponerse. Si no está a cielo abierto, tarda cerca de 1.000 años.

V F 4. En 2018, la producción global de plásticos fue de 359 millones de toneladas, un peso similar al aproximado de la población mundial.

V F 5. Si la producción de plástico continúa al mismo ritmo, los océanos tendrán más plástico que peces para el año 2050.

V F 6. La mayoría de los materiales plásticos de un solo uso, que la gente utiliza, son reciclados al finalizar su uso.

V F 7. Millones de aves, peces, ballenas y tortugas marinas mueren cada año por comer plásticos que se encuentran en el mar o playas.

V F 8. El principal problema sobre el uso del plástico es que no hay alternativas para reemplazarlo por otros materiales.

V F 9. Los artículos plásticos son buenos o malos dependiendo del uso que le demos y cómo son desechados.

V F 10. Los microplásticos pueden llegar a nuestros alimentos y bebidas sin que nos demos cuenta.

1. verdadero
2. verdadero
3. verdadero
4. verdadero
5. verdadero
6. falso
7. verdadero
8. falso
9. verdadero
10. verdadero

Homework Guide (Reflection Exercise)

MODULE #2: A WORLD of plastic

Oceans and plastics quiz questions

Instructions: Mark next to the statement if you think it is T or F

TRUE **T** FALSE **F**

T F 1. The oceans cover about 70% of the Earth's surface.

T F 2. Climate change and microplastics are threatening the life of the oceans.

T F 3. Each plastic bottle takes about 450 years to decompose. If that bottle is in a landfill, it takes about 1,000 years.

T F 4. In 2018, the global production of plastics was 359 million tons, a weight similar to the approximate weight of the world population.

T F 5. If plastic production continues at the same rate as right now, the oceans will have more plastic than fish by 2050.

T F 6. Most of the single-use plastic materials are recycled at the end of their use.

T F 7. Millions of birds, fish, whales, and turtles die each year from eating plastic found in the sea or on beaches.

T F 8. The main problem with plastic is that there are no other materials that can be used instead of plastic.

T F 9. Plastic items are good or bad depending on how we use them and how they are disposed of.

T F 10. Microplastics can get into our food and drinks without us realizing it.

1. true
2. true
3. true
4. true
5. true
6. false
7. true
8. false
9. true
10. true

COM-B 2

1. La semana pasada ¿fue posible no quemar plástico (por ejemplo, pequeños envoltorios y paquetes) en el fogón de su cocina?

SI ☐ NO ☐

2. ¿Qué podrían hacer en su hogar para crear un ambiente con menos artículos plásticos?

3. ¿Qué pueden hacer en su comunidad para lograr vivir en un medio ambiente libre de contaminación por plásticos?

4. ¿Qué tanto apoyo recibiría de su familia, si tratara de reducir la basura plástica en su hogar?

5. ¿Quiénes en su comunidad deberían involucrarse para reducir los problemas que se generan por los plásticos?

7. ¿Quiénes cree usted que deberían de encargarse del manejo de la basura plástica en su comunidad?

8. ¿Cree usted que otras personas en su comunidad quieran trabajar juntos, para resolver los problemas que se generan por los plásticos?

SI ☐ NO ☐

¿Por qué?

COM-B - MÓDULO 2: Un mundo de plástico/ A world of plastic

	Pregunta/Question	Respuesta/Answer
1	La semana pasada, ¿fue posible NO quemar plástico (pequeños envoltorios y paquetes, por ejemplo) en su fogón de la cocina?<br>Last week, was it possible to NOT burn plastic (small wrappers and packages, for example) in your kitchen fire?	1. Si<br>2. No<br>1. Yes<br>2. No
2	¿Qué podrían hacer en su hogar para crear un ambiente con menos artículos plásticos?<br>What could be done in your home to create an environment with less plastic items?	[texto]
3	¿Qué pueden hacer en su comunidad para lograr vivir en un medio ambiente libre de contaminación por plásticos?<br>What can you do in your community to be able to live in an environment free of plastic contamination?	[texto]
4	¿Qué tanto apoyo recibiría de su familia, si tratara de reducir la basura plástica en su hogar?<br>How much support would you receive from your family if you tried to reduce plastic trash in your home?	[texto]
5	¿Quiénes en su comunidad deberían involucrarse para reducir los problemas que se generan por los plásticos?<br>Who in your community should get involved to reduce the problems caused by plastics?	[texto]
6	¿Quiénes cree usted que deberían de encargarse del manejo de la basura plástica en su comunidad?<br>Who do you think should be in charge of plastic waste management in your community?	[texto]
7	¿Cree usted que otras personas en su comunidad quieran trabajar juntos para resolver los problemas que se generan por los plásticos? ¿Por qué sí? ¿Por qué no?<br>Do you think other people in your community want to work together to solve the problems caused by plastics? Why? Why not?	[texto]

Session 3

Impacts of Plastic on the Environment

Theme: Plastics in waterways and oceans, including microplastics; the 4 R's (refuse, reduce, recycle, repurpose)

Guía de Trabajo en Casa (Ejercicio de Reflexión)

MÓDULO #3: IMPACTOS del plástico en el MEDIO AMBIENTE

Pronostica cuánto tiempo tardan en degradarse ciertos materiales. Te presentamos varias fechas, escoge la que corresponde a cada uno. Escribe la letra de la fecha que elegiste, puedes repetir la letra. Te mostramos un ejemplo.

A 1-3 meses B 3-6 meses C 1-5 años D 5-20 años

E 10-50 años F 100-300 años G 300-600 años

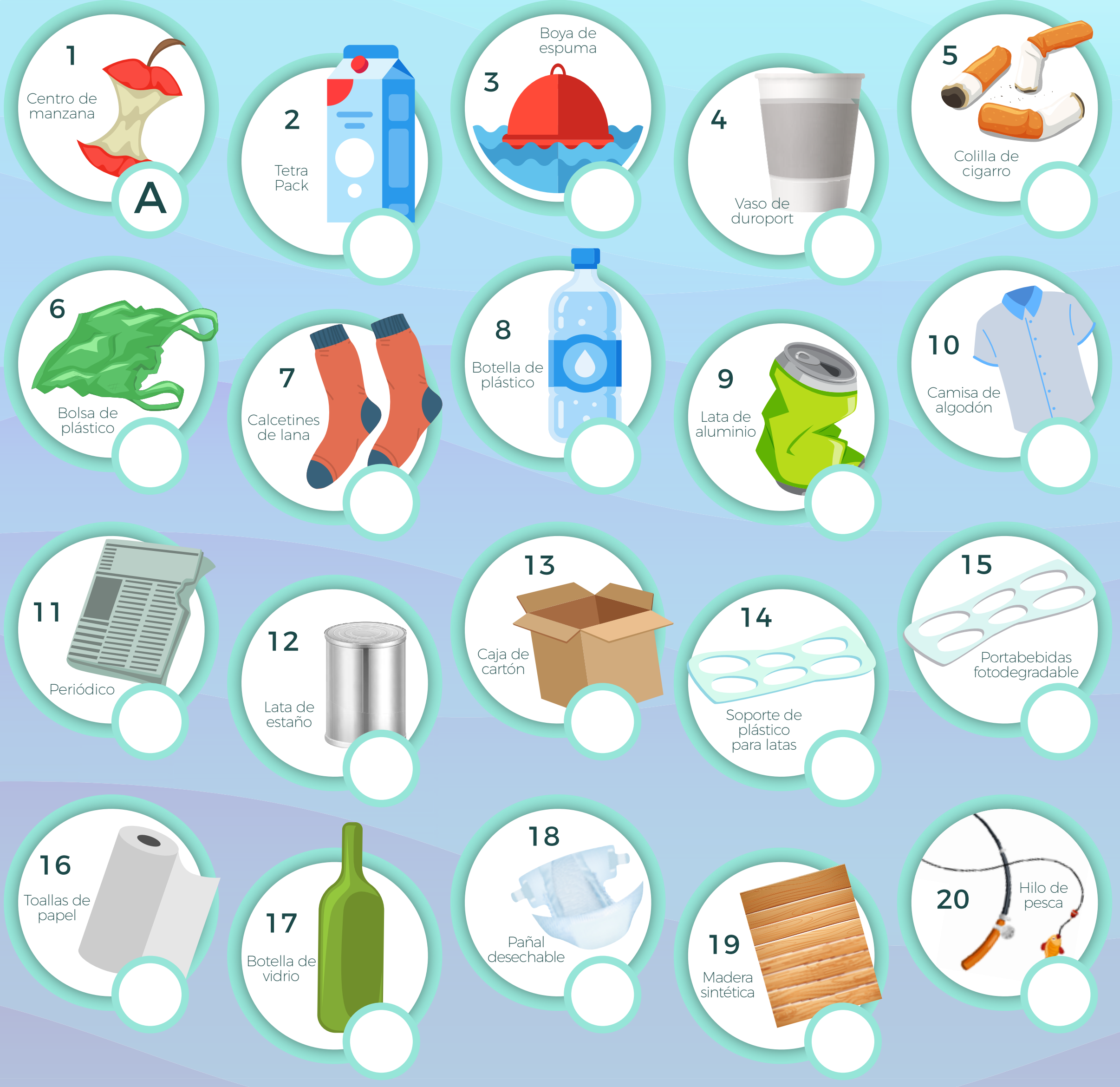

1-A 2 meses **2-A** 3 meses **3-E** 50 años **4-E** 50 años **5-C** 1-5 años **6-E** 10-20 años **7-C** 1-5 años **8-G** 450 años **9-F** 200 años **10-B** 5 meses
11-A 1,5 meses **12-E** 50 años **13-A** 2 meses **14-G** 400 años **15-B** 6 meses **16-A** 1 mes **17-** indeterminado **18-G** 450 años **19-C** 1-3 años **20-G** 600 años

Homework Guide (Reflection Exercise)

MODULE #3: IMPACTS of plastic on the ENVIRONMENT

Participants will decide how long it takes for certain materials to desintegrate or degrade. They will choose which date corresponds to each item.

A 1-3 months   B 3-6 months   C 1-5 years   D 5-20 years

E 10-50 years   F 100-300 years   G 300-600 years

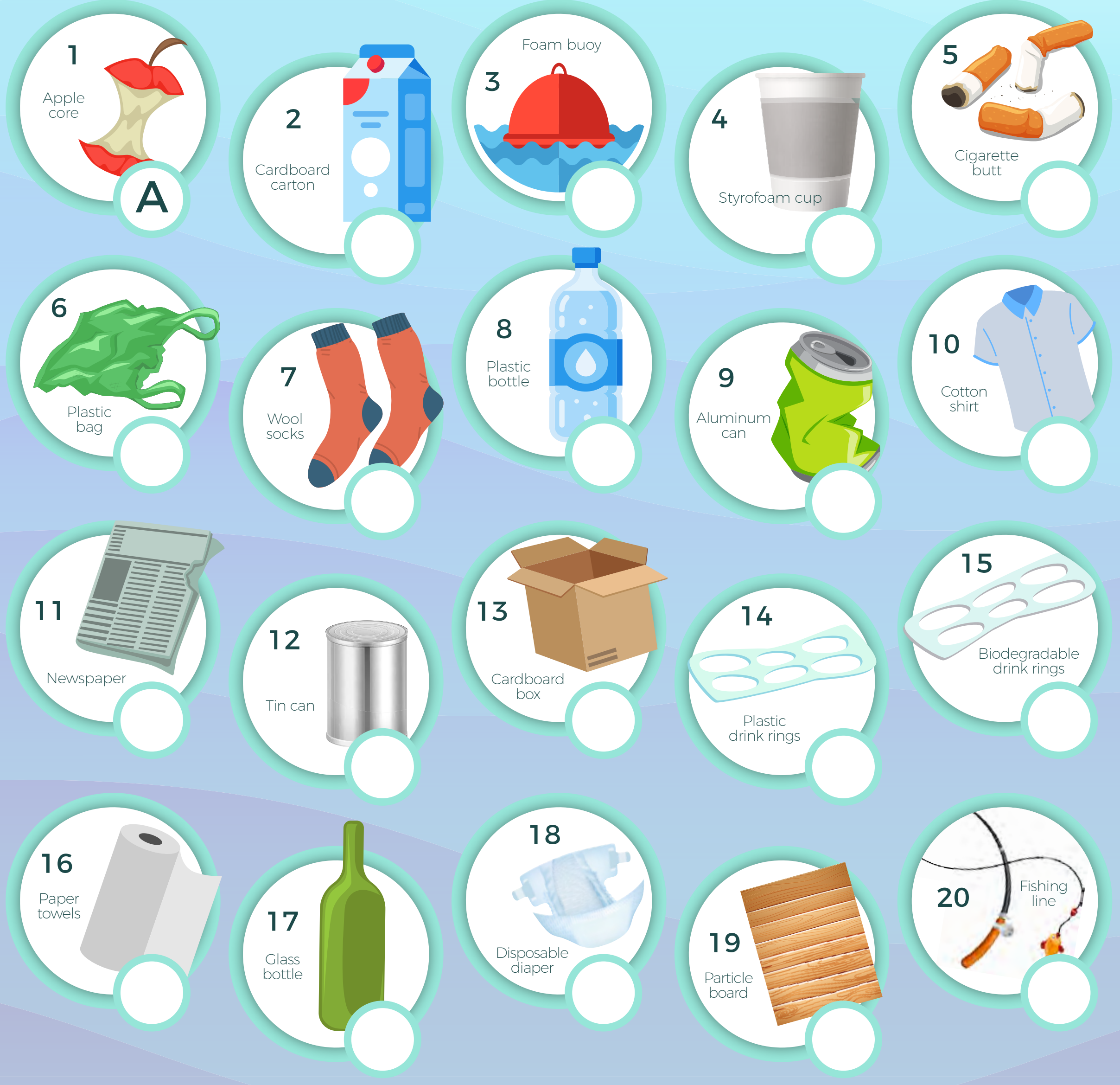

1-A 2 months **2-A** 3 months **3-E** 50 years **4-E** 50 years **5-C** 1-5 years **6-E** 10-20 years **7-C** 1-5 years **8-G** 450 years **9-F** 200 years **10-B** 5 months
11-A 1.5 months **12-E** 50 years **13-A** 2 months **14-G** 400 years **15-B** 6 months **16-A** 1 month **17-** indeterminate **18-G** 450 years **19-C** 1-3 years **20-G** 600 years

COM-B
3

1. La semana pasada ¿Con quiénes de su familia habló sobre cómo reducir el uso de plásticos en su hogar y comunidad?

2. ¿Cómo se sintió cuando aprendió acerca de todos los daños que provocan los plásticos en la naturaleza, especialmente en los animales?

3. ¿Cree que al entender ese daño, cambiará la forma en que su hogar maneja el plástico?

SI NO

¿Por que?

3a. ¿Qué cambios podrían hacer en su hogar?

4. ¿Cuáles son las consecuencias de la contaminación ambiental?

5. ¿Usted conoce a personas en su comunidad que eligen usar menos plástico?

SI NO

6. ¿Cuál cree usted que es la razón por la que una persona decide usar menos plástico?

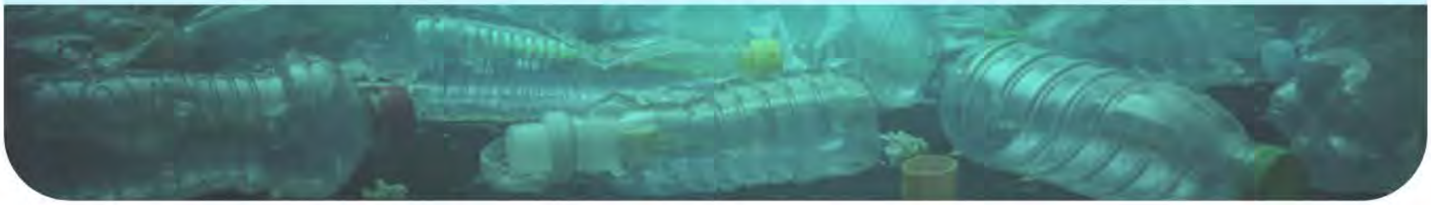

COM-B - MÓDULO 3: Impactos del plástico en el medio ambiente/ Impacts of plastic on the environment

	**Pregunta/Question**	**Respuesta/Answer**
1	La semana pasada ¿Con quiénes de su familia habló sobre cómo reducir el uso de plásticos en su hogar y comunidad?<br>During last week, who did you talk with in your family about reducing plastic in your home and community?	[texto]
2	¿Cómo se sintió cuando aprendió acerca de todos los daños que provocan los plásticos en la naturaleza, especialmente en los animales?<br>How did you feel when you learned about all the damage plastics do to nature, especially animals?	[texto]
3	¿Cree que al entender ese daño, cambiará la forma en que su hogar maneja el plástico? ¿Por qué sí? ¿Por qué no? ¿Qué cambios podrían hacer en su hogar?<br>Do you think understanding those damages will change the way plastic is managed in your home? Why so? Why not? What changes could be made in your home?	[texto]
4	¿Cuáles son las consecuencias de la contaminación ambiental?<br>What are the consequences of environmental pollution?	[texto]
5	¿Usted conoce a personas en su comunidad que eligen usar menos plástico?<br>Do you know other people in your community that choose to use less plastic?	1. Si<br>2. No<br>1. Yes<br>2. No
6	¿Cuál cree usted que es la razón por la que una persona decide usar menos plástico?<br>What do you think is the reason why a person decides to use less plastic?	[texto]

Session 4

Impacts of Plastic on Health

Theme: Contaminants in burning plastic, and health implications of exposure; differences between junk food in plastic bags and food like fruits and vegetables, typically not wrapped in plastic

Guía de Trabajo en Casa (Ejercicio de Reflexión)

MÓDULO #4: IMPACTOS del plástico en la SALUD

Ejercicio de reflexión en casa ¿Qué cosas podría cambiar en mi consumo de plástico?
Escribe en el cuadro izquierdo todos los artículos que son plásticos o contienen partes plásticas que utilizas diaria u ocasionalmente. Colócalo en la columna a la cual crees que corresponde. Las categorías **importante** y **no importante** se refieren a si esos productos son necesarios (importante) o bien, puedes eliminar su uso (no importante). Luego, escribe si ese producto puede sustituirse o no por otro material y por cuál. Por ejemplo, ***cepillo de dientes*** en importante, ***bote de enjuague bucal*** en no importante, si ese fuera el caso. Un cepillo de dientes plástico podría sustituirse por uno de bambú.

¿Qué artículos plásticos utilizamos en casa diaria o frecuentemente?		¿Lo podemos sustituir por otro tipo de material?		
**Importante**	**No importante**	**No**	**Si**	**¿Cuál?**
Cepillo de dientes			✓	Cepillo de dientes de bambú
	Bote de enjuague bucal	✓		

Homework Guide (Reflection Exercise)

MODULE #4: IMPACTS of plastic on HEALTH

This is an exercise for you to reflect on at home: Ask yourself: What things could I change about how I use plastic? On the left side of the grid list all the items that are plastic or contain plastic parts that you use daily - or even occasionally. Place the item in the column to which you think it belongs, for instance, is it **important** (necessary) or **not important** (you can do without it)? On the right side of the grid, answer yes or no if you think you can find a substitution for the plastic item. Write down what you could use instead. For example, a ***toothbrush*** is **important**, and we might substitute a bamboo toothbrush for a plastic one. ***Mouthwash bottle*** might be **not important**, in your case.

What plastic items do I use daily or even occasionally?		Can I replace this plastic item with something made with another material that is not plastic?		
Important	Not important	No	Yes	What?
Toothbrush			✓	Bamboo toothbrush
	Mouthwash bottle	✓		

COM-B

1. La semana pasada ¿Cuáles son algunas de las cosas que usted hizo para reducir el impacto del plástico en la naturaleza que está alrededor de su hogar?

2. ¿Cómo se sintió cuando aprendió acerca de los daños que provocan los plásticos en la salud, especialmente en los niños?

3. ¿Le preocupa que el plástico pueda afectar la salud de su familia?

Si mucho | Si un poco | No

4. ¿Cómo cree usted que la basura plástica afecta la salud de su familia?

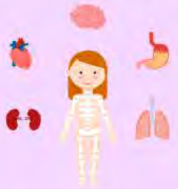

5. ¿Usted cree que quemar basura plástica dentro del fogón de la cocina daña la salud de una persona?

Sí | No | Nosé

6. Si en su hogar dejaran de quemar plásticos, ¿qué pasaría? (marcar las fotos que aplican)

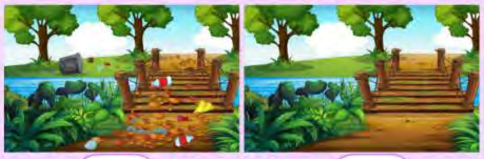
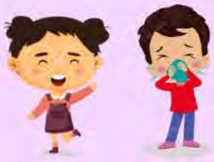
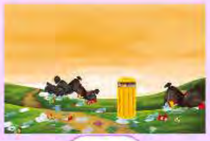
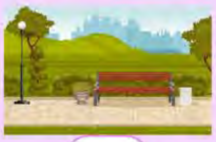

7.¿Qué necesitaría usted para poder hablar con su familia, amigos u otras personas en la comunidad sobre los efectos negativos del plástico en la salud?

COM-B MÓDULO 4: Impactos del plástico en la salud/
Impacts of plastic on health

	Pregunta/Question	Respuesta/Answer
1	La semana pasada ¿Cuáles son algunas de las cosas que usted hizo para reducir el impacto del plástico en la naturaleza que está alrededor de su hogar?<br>What are some of the things you did to reduce the impact of plastic on nature around your home last week?	[texto]
2	¿Cómo se sintió cuando aprendió acerca de los daños que provocan los plásticos en la salud, especialmente en los niños?<br>How did you feel when you learned about the health harms of plastics, especially for children?	[texto]
3	¿Le preocupa que el plástico pueda afectar la salud de su familia?<br>Are you worried that plastic could affect your family's health?	1. Si, mucho<br>2. Si, un poco<br>3. No<br>1. Yes, a lot<br>2. Yes, a little<br>3. No
4	¿Cómo cree usted que la basura plástica afecta la salud de su familia?<br>How do you think that plastic trash affects your family's health?	[texto]
5	¿Usted cree que quemar basura plástica dentro del fuego de la cocina daña la salud de una persona?<br>Does burning plastic trash inside the kitchen fire harm a person's health?	1. Si<br>2. No<br>3. No se<br>1. Yes<br>2. No<br>3. I don't know
6	Si en su hogar dejaran de quemar plásticos, ¿qué pasaría? (Marcar las imágenes que aplican)<br>If your home stopped burning plastic, what would happen? (Check the images that apply)	• *Ambiente limpio/otro*<br>• *con plástico*<br>• *Nino sano/enfermo*<br>• *Air contaminado/limpio*<br>• *Environment with/without plastic*<br>• *Healthy/sick child*<br>• *Contaminated/clean air*
7	¿Qué necesitaría usted para poder hablar con su familia, amigos u otras personas en la comunidad sobre los efectivos negativos del plástico en la salud?<br>What would you need to be able to talk to your family, friends or others in the community about the negative health effects of plastic?	[texto]

Session 5

Sustainable Alternatives to Reduce the Use of Plastics

Theme: Sustainable alternatives to plastic and reducing plastic litter in the community

Guía de Trabajo en Casa (Ejercicio de Reflexión)

MÓDULO #5: Alternativas SUSTENTABLES para reducir el uso de PLÁSTICOS

Instrucciones: Marcar al final de cada día los artículos plásticos de un solo uso que utilizaron o consumieron. Al final de una semana veremos cuántos artículos plásticos se consumieron en total.

Mi diario de plásticos de UN SOLO USO

	Lunes	Martes	Miércoles	Jueves	Viernes	Sábado	Domingo	Total de artículos
Bolsas plásticas								
Pajillas								
Botellas plásticas								
Platos, vasos y cubiertos desechables								
Envoltorios de chucherías								
Contenedores o envoltorios plásticos de comida								
Contenedores o envoltorios plásticos de higiene personal								

Otros artículos plásticos de un solo uso que utilizaron esta semana:

Homework Guide (Reflection Exercise)

Módule #5: SUSTAINABLE alternatives to reduce the use of PLASTICS

Instructions: At the end of each day make a list of the single-use plastic items you used or food that came packaged in plastic. At the end of the week, we will see how many plastic items were purchased or used in total.

My SINGLE USE Plastics Diary

	Monday	Tuesday	Wednesday	Thursday	Friday	Saturday	Sunday	Total number of articles
Plastic bags								
Straws								
Plastic bottles								
Disposable plates, cups and utensils								
Junk food wrappers								
Plastic food containers								
Plastic items used for personal hygiene (soap, shampoo)								

Other single-use plastic items used this week:

COM-B
5

1. La semana pasada ¿Qué alternativas encontró para no quemar plásticos, tanto afuera como adentro del hogar?

2. ¿Qué pueden hacer las personas para resolver los problemas que ocasionan los plásticos en su hogar y en su comunidad?

3. ¿Usted ha visto en su comunidad que reutilicen los plásticos para otros fines, por ejemplo, hacer maceteros con botellas de refresco u otros fines?

SI ☐ NO ☐

4. ¿Qué otras cosas ha visto usted que la gente hace con materiales plásticos?

5. ¿Cree usted que hay personas en su comunidad que se preocupan por evitar la quema de materiales plásticos?

SI ☐ NO ☐

Responda: ¿Por qué cree que a unos les preocupa?

Responda: ¿Por qué cree que a otros no les preocupa?

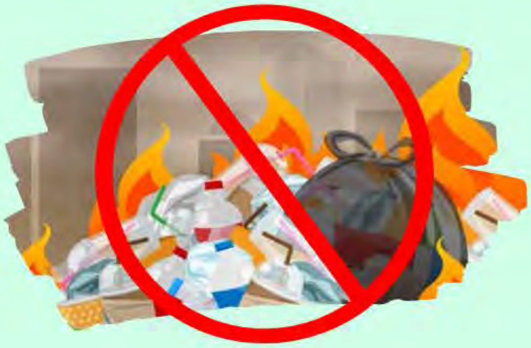

COM-B - MÓDULO 5: Alternativas sustentables para reducir el uso de plásticos/ Sustainable alternatives to reduce the use of plastics

	Pregunta/Question	Respuesta/Answer
1	La semana pasada ¿qué alternativas encontró para no quemar plástico, tanto afuera como adentro del hogar?<br>Last week, what alternatives did you find to avoid burning plastic, both outside and inside the home?	[texto]
2	¿Qué pueden hacer las personas para resolver los problemas que ocasionan los plásticos en su hogar y en su comunidad?<br>What can people do to address/fix the problem caused by plastic in their home and in the community?	[texto]
3	¿Usted ha visto en su comunidad que reutilicen los plásticos para otros fines, por ejemplo, hacer maceteros con botellas de refresco u otros fines?<br>Do you see other people who are reusing plastic for other things, like flowerpots or planters from soda bottles?	1. Si<br>2. No<br>1. Yes<br>2. No
4	¿Qué otras cosas ha visto que la gente hace con materiales plásticos?<br>What other things have you seen that people make with plastic materials?	[texto]
5	¿Cree usted que hay personas en su comunidad que se preocupan por evitar la quemar de plásticos?<br>5.1 ¿Por qué cree que a unos les preocupan?<br>5.2 ¿Por qué cree que a otros no les preocupan?<br>Do you think that there are people in your community who care about stopping plastic burning?<br>5.1 Why do you think some are concerned?<br>5.2 Why do you think others don't care?	1. Si<br>3. No<br>[texto]<br>1. Yes<br>2. No<br>[text]

Session 6
Recycling Plastic Materials

Theme: Recycling plastic materials; distinguishing materials that can and cannot be recycled

Guía de Trabajo en Casa

MÓDULO #6: RECICLAJE de materiales PLÁSTICOS

Identifica qué artículos de los que se muestran en la hoja pueden reciclarse.

Marca con un cheque los que creas que **sí,**

y con una cruz los que creas que **no.**

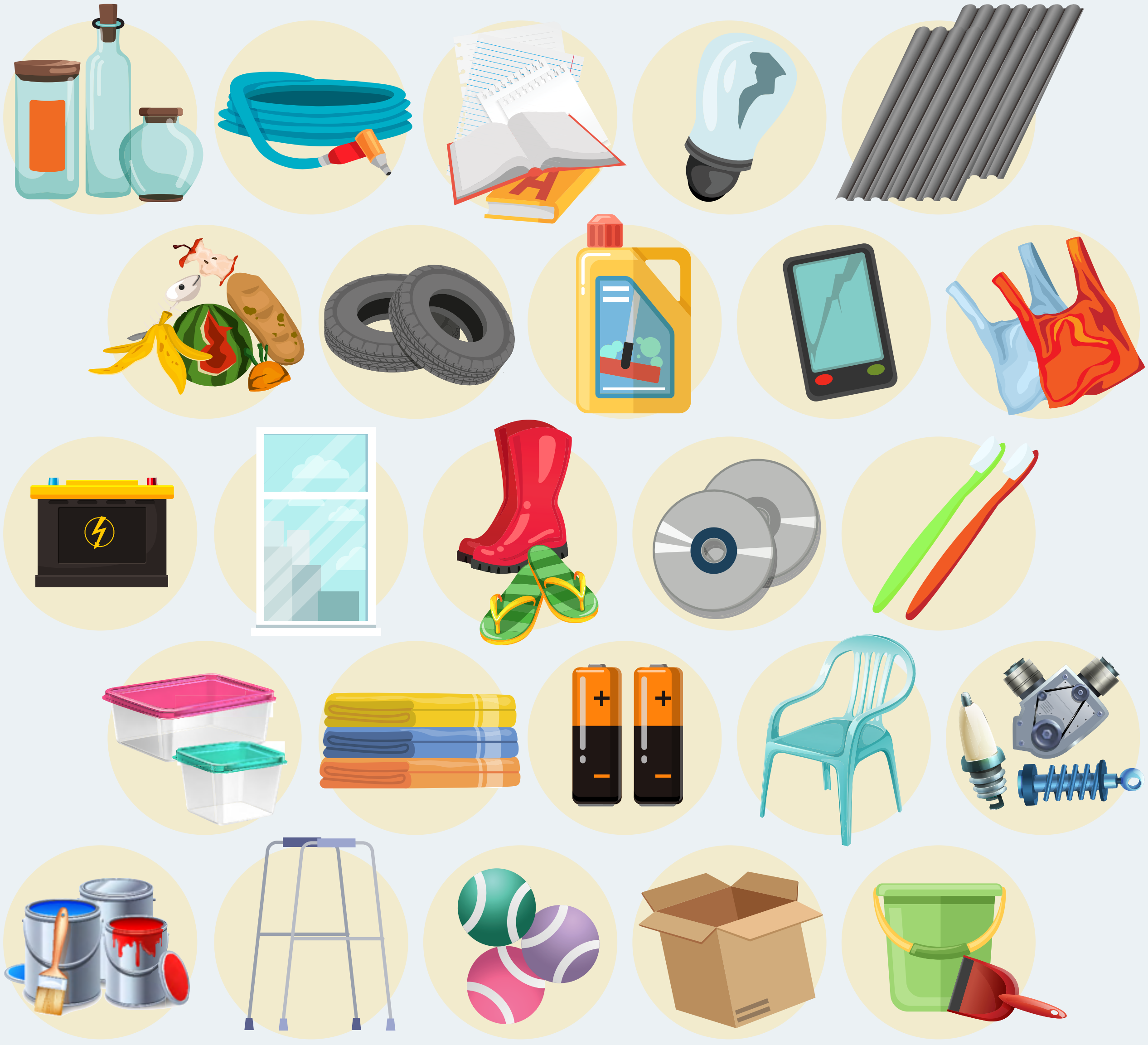

FILA #1 SI, SI, SI, NO, SI FILA #2 SI, NO, SI/NO, SI, NO FILA #3 NO, SI/NO, SI, NO, NO FILA #4 SI, NO, NO, SI, SI FILA #5 NO, SI, SI, SI, SI.

Homework Guide (Reflection Exercise)

Módule #6: RECYCLING PLASTIC materials

Identify which items below can be recycled.

✓ Mark with a check items that **CAN be recycled**

✗ Mark with a cross items that **CANNOT be recycled**

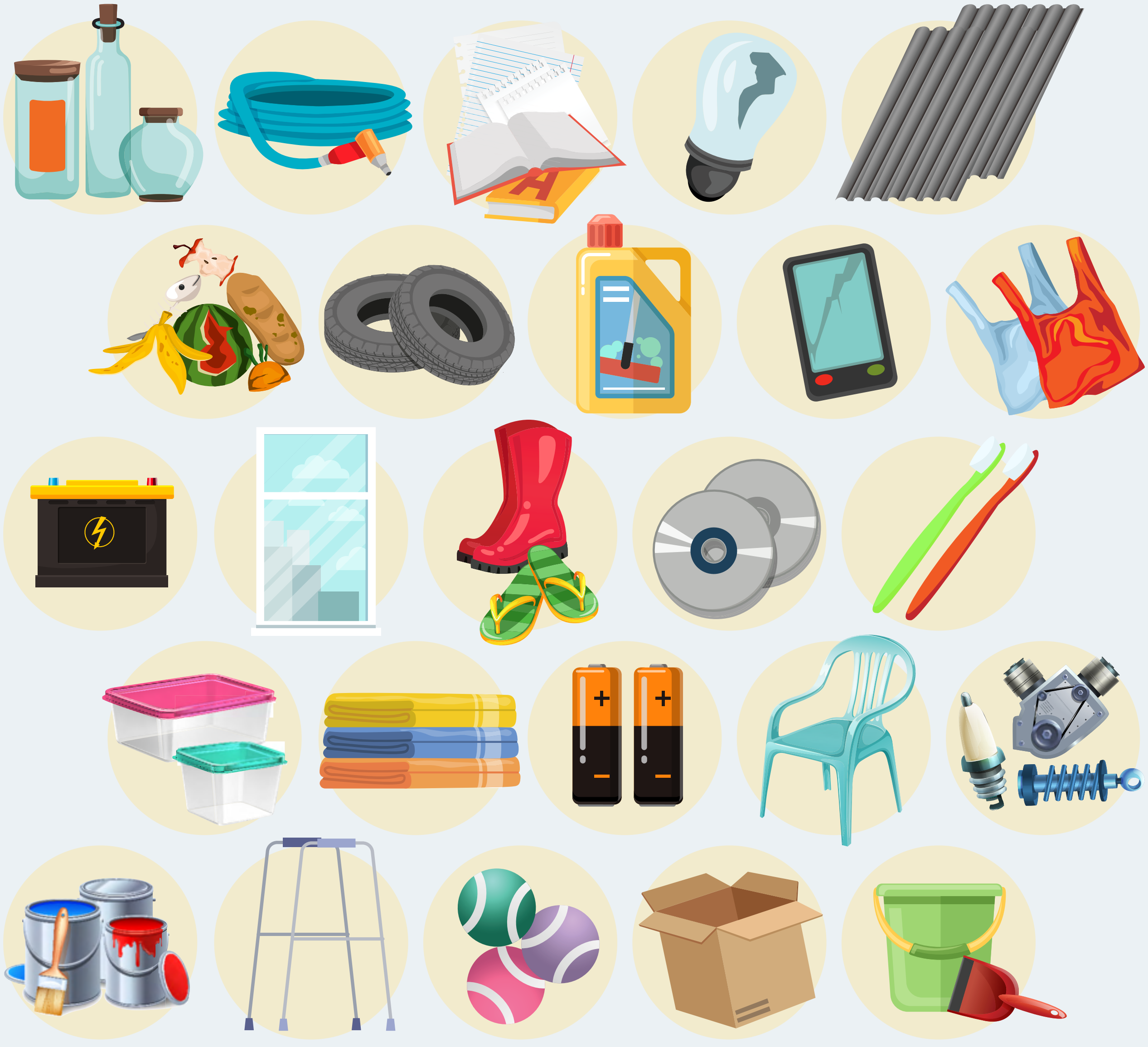

ROW #1 YES, YES, YES, NO, YES **ROW #2** YES, NO, YES/NO, YES, NO **ROW #3** NO, YES/NO, YES, NO, NO
ROW #4 YES, NO, NO, YES, YES **ROW #5** NO, YES, YES, YES, YES

1. La semana pasada ¿Pudo usted hablar con sus amigos, familiares o vecinos, sobre qué opinan acerca de quemar la basura plástica?

SI ☐ NO ☐

¿Qué dijeron ellos sobre evitar la quema de basura plástica?

3. ¿Usted conoce a personas en su comunidad que reciclen materiales plásticos?

SI ☐ NO ☐

4. ¿Conoce usted personas de su comunidad o programas en su comunidad, que compren materiales reciclados?

SI ☐ NO ☐

5. ¿Qué tan difícil o fácil es para usted clasificar la basura para poder reciclarla?

☐ Facil ☐ Dificil ☐ No sé

6.¿Cuáles cree que serían los beneficios en su comunidad si la gente reciclara los materiales plásticos?

7. ¿Qué tan difícil o fácil sería para los miembros de su comunidad iniciar un programa de reciclaje?

☐ Facil ☐ Dificil ☐ No sé

8. ¿Cuáles serían los mayores problemas que encontraría la gente al intentar reciclar materiales plásticos en su comunidad?

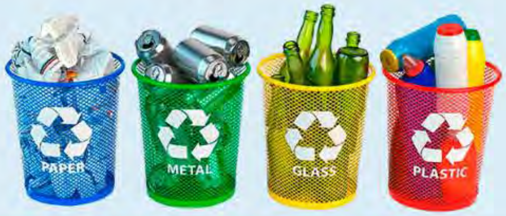

COM-B - MÓDULO 6: Reciclaje de materiales plásticos/
Recycling plastic materials

	Preguntas/Questions	Respuestas/Answers
1	La semana pasada ¿Pudo usted hablar con sus amigos, familiares o vecinos, sobre qué opinan acerca de quemar la basura plástica?<br>Last week did you talk to your friends, family or neighbors about their views on plastic trash burning?	1. Si<br>2. No<br>1. Yes<br>2. No
2	¿Qué dijeron ellos sobre evitar la quema la basura de plástica?<br>What did they say about avoiding burning plastic trash?	[texto]
3	¿Usted conoce a personas en su comunidad que reciclen materiales plásticos?<br>Do you know people in your community who recycle plastic materials?	1. Si<br>2. No<br>1. Yes<br>2. No
4	¿Conoce usted personas de su comunidad o programas en su comunidad, que compren materiales reciclados?<br>Do you know people from your community, or programs in your community, who buy recycled materials?	1. Si<br>2. No<br>1. Yes<br>2. No
5	¿Qué tan difícil o fácil es para usted clasificar la basura para poder reciclarla?<br>How difficult or easy would it be for you to classify your waste to be able to recycle?	1. Difícil<br>2. Fácil<br>3. No se<br>1. Difficult<br>2. Easy<br>3. I don't know
6	¿Cuáles cree que serían los beneficios en su comunidad si la gente reciclara los materiales plásticos?<br>What do you think would be the benefits in your community if people recycled plastic materials?	[texto]
7	¿Qué tan difícil o fácil sería para los miembros de su comunidad iniciar un programa de reciclaje?<br>How difficult or easy would it be for members of your community to start a recycling program?	1. Difícil<br>2. Fácil<br>3. No se<br>1. Difficult<br>2. Easy<br>3. I don't know
8	Cuáles serían los mayores problemas que encontraría la gente al intentar reciclar materiales plásticos en su comunidad?<br>What would be the biggest problems people would encounter when trying to recycle plastic material in their village?	[texto]

Session 7

Environmental Justice

Theme: Environmental justice; community empowerment

Guía de Trabajo en Casa

MÓDULO #7: JUSTICIA AMBIENTAL

Observa los dibujos e identifica ¿Cuáles de estos problemas ambientales hay en tu comunidad?, ¿Dónde se encuentran? y ¿Qué impactos ambientales y en salud pueden tener?. Escribe en el cuadro inferior, según el ejemplo.

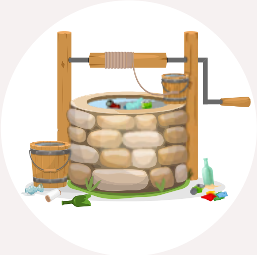
Pozos contaminados

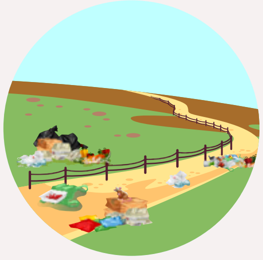
Caminos llenos de basura

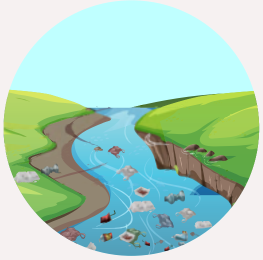
Ríos o arroyos contaminados con basura

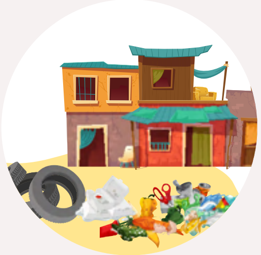
Basureros cercanos a viviendas

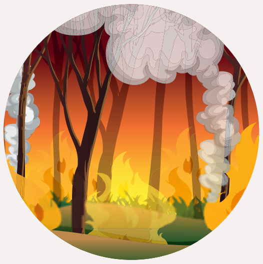
Incendios forestales

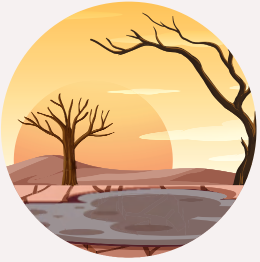
Áreas deforestadas

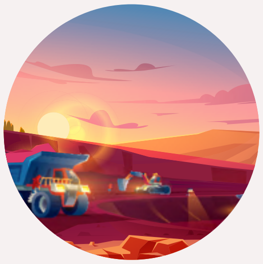
Extracción de recursos por presencia de la minería

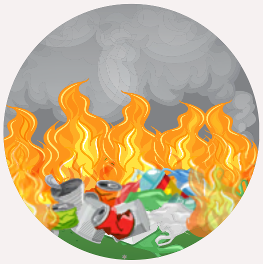
Quema de basura

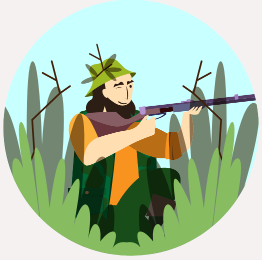
Cacería ilegal de animales salvajes

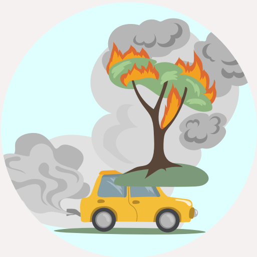
Humo tóxico, smog

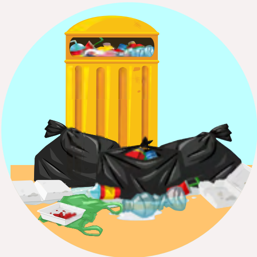
Presencia de muchos plásticos

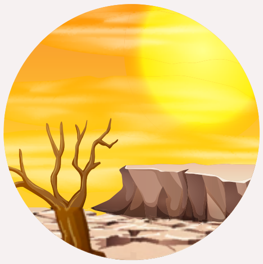
Sequía, falta de agua

Problema	Lugar	Impacto
**Ejemplo:** Llantas tiradas	Campo de fútbol	Pérdida de calidad de suelo

Homework Guide (Reflection Exercise)

Módule #7: ENVIRONMENTAL JUSTICE

Instructions: Look at the drawings and identify: Which of these environmental problems are in your community? Where are they located? What are the environmental and health impacts you think the problem may cause?

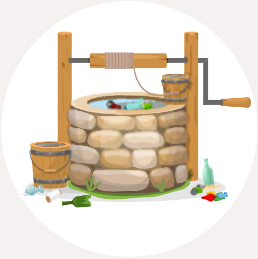
Contaminated wells

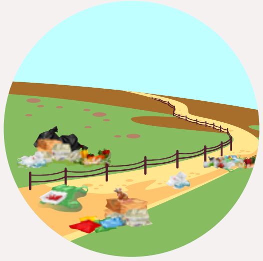
Roads full of garbage

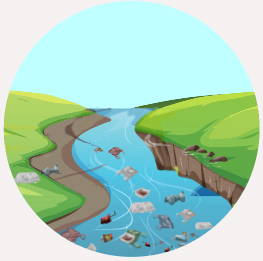
Rivers or streams filled with garbage

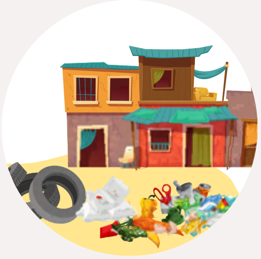
Garbage dumps close to homes

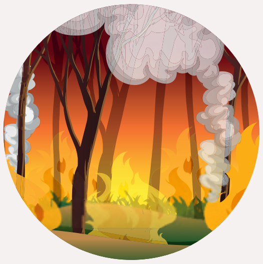
Forest fires

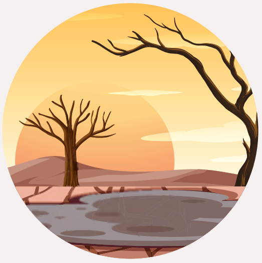
Deforested areas

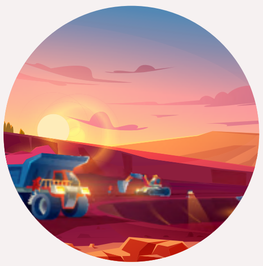
Resource extraction by mining companies

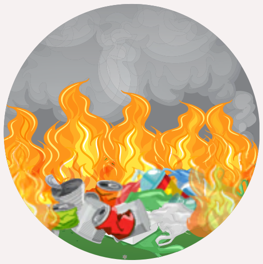
Burning garbage

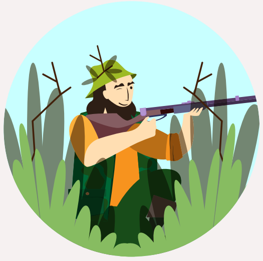
Illegal hunting of wild animals

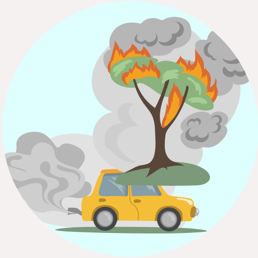
Toxic smoke, smog

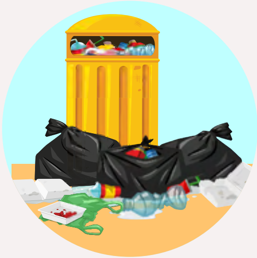
Plastic litter

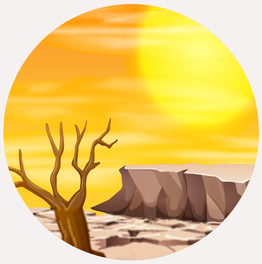
Drought; lack of water

Problem	Place	Impact
**Example:** discarded tires	Football field	Poor soil quality

COM-B
7

1. La semana pasada cuando fue de compras, ¿Qué empaques de productos vio usted que podría reciclar fácilmente o que estén hechos con materiales reciclados?

2. ¿Usted cree que la acción en la foto es un beneficio para su comunidad?

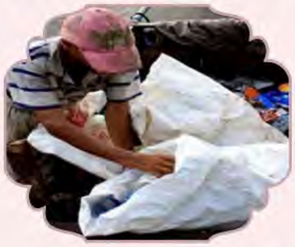

SI NO

¿Por qué?

3. Indique cuáles de las siguientes acciones puede hacer en grupo y cuáles puede hacer usted solo:

RECICLAR

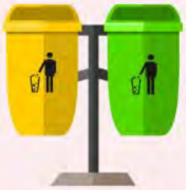

EN GRUPO
SOLO

TREN DE ASEO

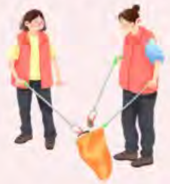

EN GRUPO
SOLO

HACER JABONES

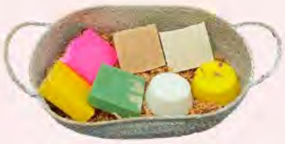

EN GRUPO
SOLO

REUTILIZAR PLASTICO

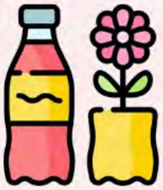

EN GRUPO
SOLO

HACER SILLONES

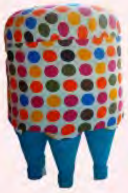

EN GRUPO
SOLO

USAR BOLSAS REUTILIZABLES

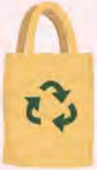

EN GRUPO
SOLO

PROHIBIR BOLSAS DE UN SOLO USO

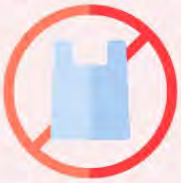

EN GRUPO
SOLO

4. ¿Cuáles cree que son los beneficios para su comunidad al tomar acción para los problemas de justicia ambiental?

5. Desde que comenzó a asistir a la reunión de ECOLECTIVOS, ¿Con cuántas personas ha hablado sobre los siguientes temas?

Cuidado del medio ambiente

Número de Personas

¿Con quienes habló?

Los problemas generados por los plásticos

Número de Personas

¿Con quienes habló?

Proyecto Ecolectivos

Número de Personas

¿Con quienes habló?

La quema de residuos plásticos

Número de Personas

¿Con quienes habló?

Reciclaje de materiales plásticos

Número de Personas

¿Con quienes habló?

Reutilizacion de materiales plásticos

Número de Personas

¿Con quienes habló?

Uso de Jabones artesanales para la reducción de empaques plásticos.

Número de Personas

¿Con quienes habló?

COM-B - MÓDULO 7: Justicia ambiental/
Environmental justice

	Pregunta/Question	Respuesta/Answer
1	La semana pasada cuando fue de compras, ¿qué empaques de productos vio usted que podría reciclar fácilmente o que estén hechos con materiales reciclados?<br>Last week when you were shopping, did you look for product packaging that can be easily recycled or that had recycled materials?	[texto]
2	¿Usted cree que la acción en la foto es un beneficio para su comunidad? Por qué?<br>Do you think the action in the photo is a benefit to your community? Why?	1. Si<br>2. No<br>[texto]<br>1. Yes<br>2. No<br>[text]
3	Indique cuales de las siguientes acciones puede hacer en grupo y cuales puede hacer usted solo:<br>Indicate which of the following actions you can do in a group and which you can do alone:	
3a	Reciclar<br>Recycling	1. En grupo<br>2. Solo<br>1. In a group<br>2. Alone
3b	Tren de aseo<br>Community clean-up	1. En grupo<br>2. Solo<br>1. In a Group<br>2. Alone
3c	Hacer jabones<br>Make soap	1. En grupo<br>2. Solo<br>1. In a Group<br>2. Alone
3d	Reutilizar plastico<br>Re-use plastic	1. En grupo<br>2. Solo<br>1. In a Group<br>2. Alone
3e	Hacer sillones<br>Make stools	1. En grupo<br>2. Solo<br>1. In a Group<br>2. Alone
3f	Usar bolsas reutilizables<br>Use reusable bags	1. En grupo<br>2. Solo<br>1. In a Group<br>2. Alone
3g	Prohibir bolsas de un solo uso\<br>Ban single use bags	1. En grupo<br>2. Solo<br>1. In a Group<br>2. Alone

4	¿Cuáles cree que son los beneficios para su comunidad al tomar acción para los problemas de justicia ambiental?<br>What do you think are the benefits to your community of taking action about the problems of environmental justice?	[texto]
5	Desde que comenzó a asistir a las reuniones de ECOLECTIVOS, ¿con cuántas personas ha hablado sobre lo siguiente termas?<br>Since you started attending the ECOLECTIVOS meetings, how many people have you talked to about the following?	
5a	Cuidado del medio ambiente<br>Caring for the environment<br>Número de personas; ¿Con quienes habló?<br>Number of people; Who did you talk to?	[#]<br>[texto]<br>[#]<br>[text]
5b	Los problemas generados por los plásticos<br>The problems generated by plastics<br>Número de personas; ¿Con quienes habló?<br>Number of people; Who did you talk to?	[#]<br>[texto]<br>[#]<br>[text]
5c	ECOLECTIVOS<br>ECOLECTIVOS<br>Número de personas; ¿Con quienes habló?<br>Number of people; Who did you talk to?	[#]<br>[texto]<br>[#]<br>[text]
5d	La quema de residuos plásticos<br>Plastic waste-burning<br>Número de personas; ¿Con quienes habló?<br>Number of people; Who did you talk to?	[#]<br>[texto]<br>[#]<br>[text]
5e	Reciclaje de materiales plásticos<br>Plastic recycling<br>Número de personas; ¿Con quienes habló?<br>Number of people; Who did you talk to?	[#]<br>[texto]<br>[#]<br>[text]
5f	Reutilización de materiales plásticos<br>Plastic re-use<br>Número de personas; ¿Con quienes habló?<br>Number of people; Who did you talk to?	[#]<br>[texto]<br>[#]<br>[text]
5g	Uso de Jabones artesanales para la reducción de empaques plásticos<br>Use of artisanal soaps to reduce plastic packaging<br>Número de personas; ¿Con quienes habló?<br>Number of people; Who did you talk to?	[#]<br>[texto]<br>[#]<br>[text]

Session 8

Community Projects and Collective Actions

Theme: Global and collective actions; importance of community projects; Guatemala initiatives

Guía de Trabajo en Casa (Ejercicio de Reflexión)

MÓDULO #8: Proyectos COMUNITARIOS & acciones COLECTIVAS

Aunque pareciera ser una labor casi imposible conseguir los cambios ambientales que deseamos y necesitamos, **para lograr frenar la contaminación por plásticos y un mal manejo de estos desechos,** es importante que cada persona inicie esta labor a través de objetivos claros y practicables. Para ello, coloca en cada círculo ¿Qué puedo hacer yo? ¿Qué podemos hacer como comunidad? ¿Qué podemos hacer como país? Define con claridad la acción.

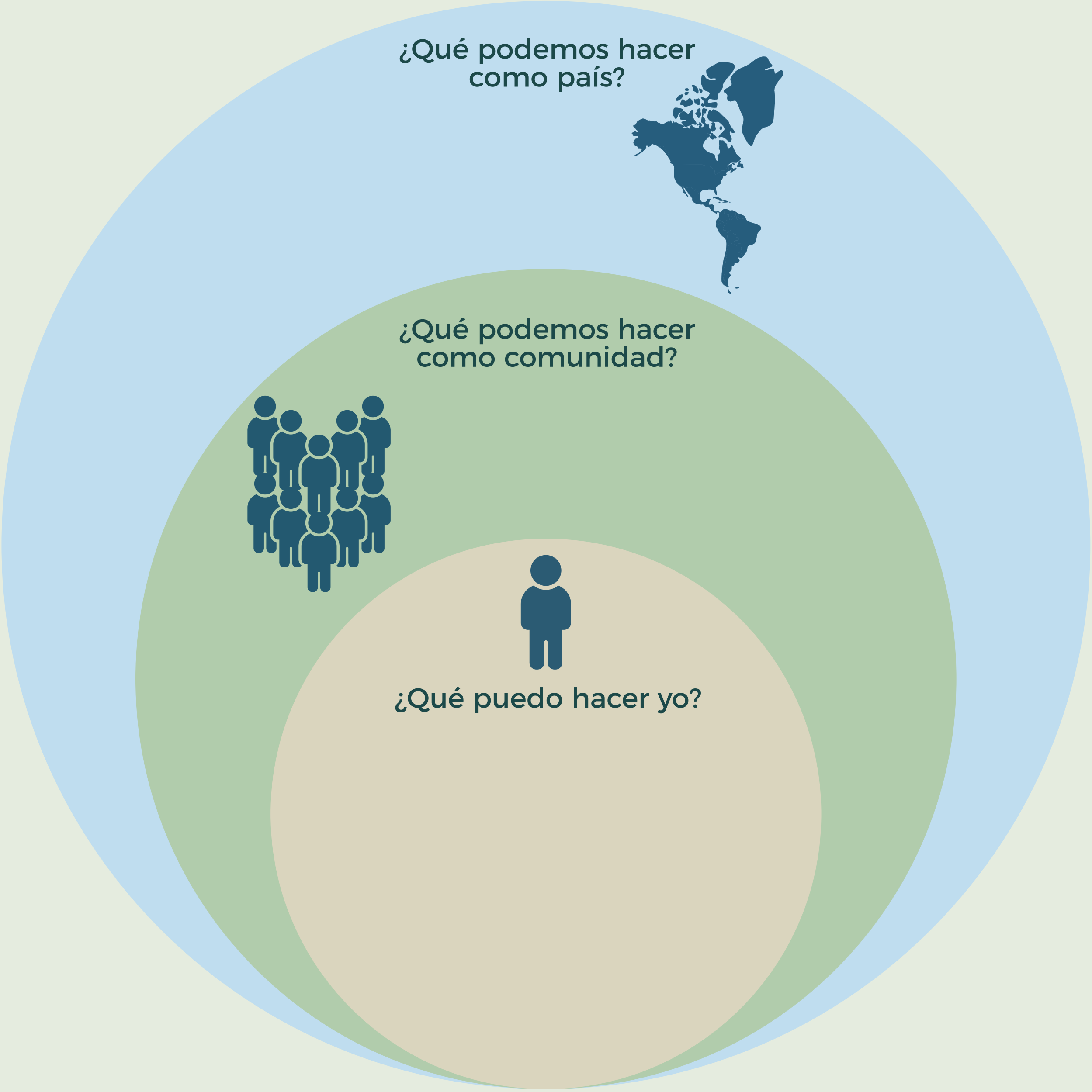

Homework Guide (Reflection Exercise)

Módule #8: COMMUNITY Projects & COLLECTIVE Actions

Instructions: Although it seems almost impossible to achieve the environmental changes that we want and need **to stop plastic pollution and poor management of this waste,** it is important that each person works towards meeting feasible objectives. To do this, write down in each circle: What can I do? What can we do as a community? What can we do as a country? Clearly define the action.

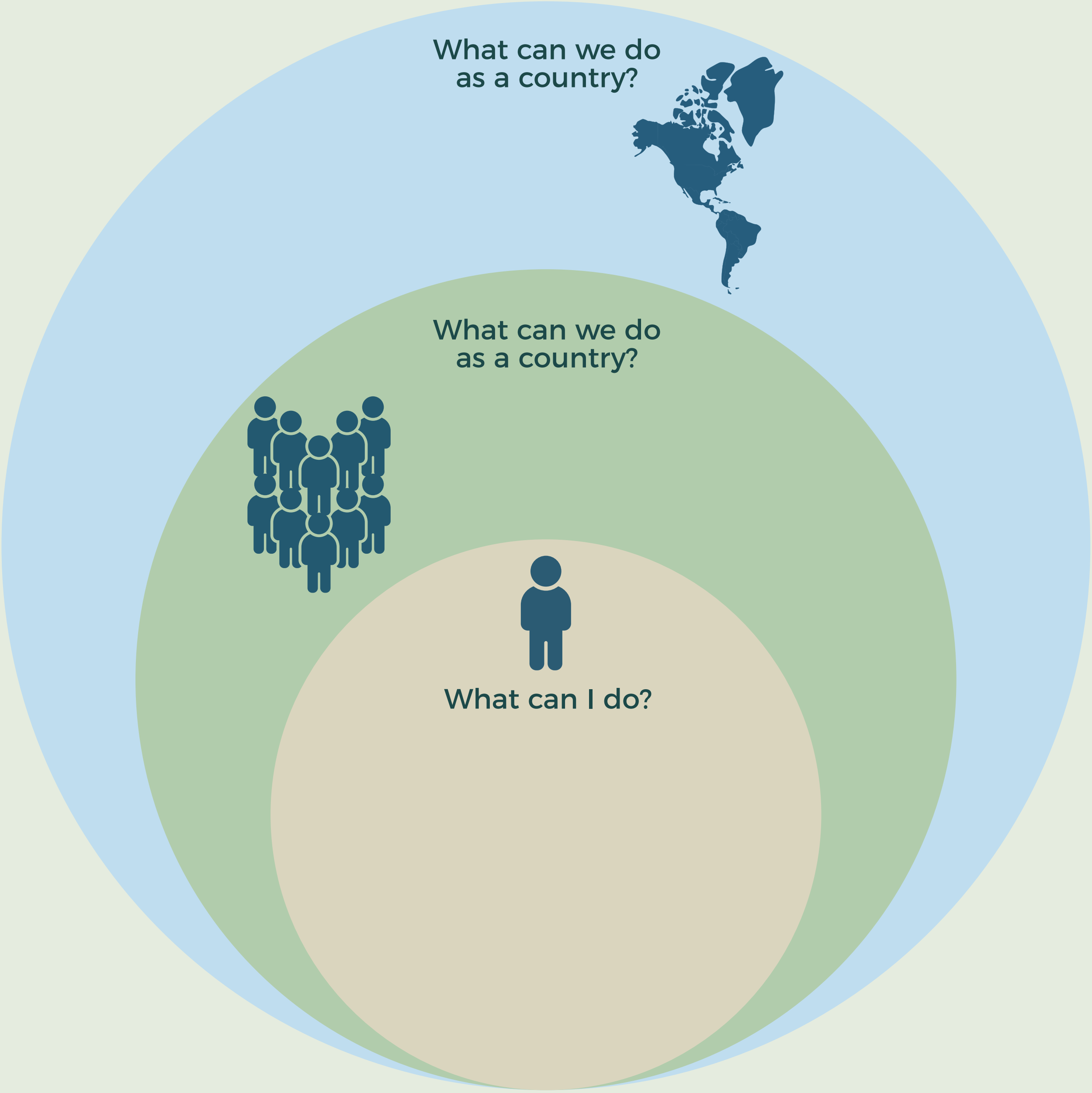

1. ¿Qué ideas tiene usted para motivar a personas de su comunidad, para que se hable sobre temas de justicia ambiental?

2. ¿Sería posible introducir en su comunidad la prohibición de las bolsas plásticas de un solo uso?

SI NO

¿Por qué?

3. ¿Qué beneficios esperaba usted al participar en las clases?

4. ¿Qué tan satisfecha estuvo usted con las clases?, siendo 1= Muy Satisfecho, 2= Medio Satisfecho y 3= Nada Satisfecho

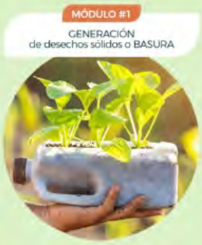

1 2 3

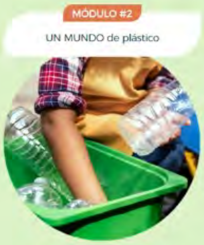

1 2 3

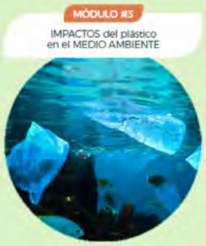

1 2 3

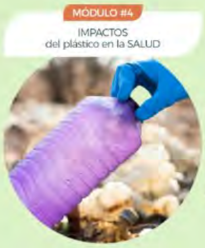

1 2 3

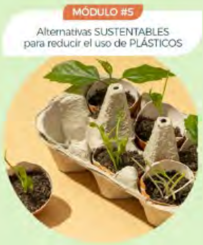

1 2 3

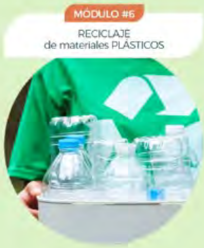

1 2 3

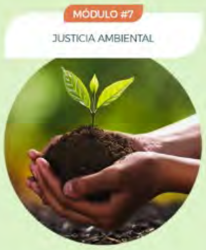

1 2 3

¿Por qué?

5. ¿Qué fue lo menos satisfactorio de todas las clases para usted?

¿Por qué?

6. ¿Qué esperaría usted que el grupo pudiera hacer diferente en el futuro?

7. ¿Qué cosas de las que aprendió en las clases, cree usted que puede llevar a cabo en su vida diaria?

8. ¿Qué cosas de las que aprendió en las clases, cree usted que puede llevar a cabo con su comunidad?

9. ¿Qué cosas de las que aprendió en las clases, será más difícil de llevar a cabo en su vida diaria, pero quiere intentarlo?

¿Por qué?

10. ¿Qué cosas de las que aprendió en las clases, será más difícil de llevar a cabo con su comunidad, pero quiere

¿Por qué?

COM-B - MÓDULO 8: Proyectos comunitarios & acciones colectivas/
Community projects & collective actions

	Pregunta/Question	Respuesta/Answer
1	¿Qué ideas tiene usted para motivar a personas de su comunidad para que se hable sobre temas de justicia ambiental?<br>What ideas do you have to motivate people in your community to speak out about environmental justice issues?	[texto]
2	¿Sería posible introducir en su comunidad la prohibición de las bolsas plásticas de un solo uso? ¿Por qué?<br>Would it be possible to introduce a ban on single-use plastic bags in your community? Why?	1. Si<br>2. No<br>[texto]<br>1. Yes<br>2. No<br>[text]
3	¿Qué beneficios esperaba usted al participar en las clases?<br>What benefits did you expect from participating in the classes?	[texto]
4	¿Qué tan satisfecha estuvo usted con las clases? ¿Por qué?<br>How satisfied were you with the classes? Why?<br><br>*[Fotos de cada módulos #s1 - 7]*<br>*[Photos of each module #s1 - 7]*	1. Muy satisfecho<br>2. Medio satisfecho<br>3. Nada satisfecho<br>[texto]<br>1. Very satisfied<br>2. Somewhat satisfied<br>3. Not at all satisfied<br>[text]
5	¿Qué fue lo menos satisfactorio de todas las clases para usted? ¿Por qué?<br>What was the least satisfying of all the classes for you? Why?	[texto]<br>[texto]
6	¿Qué esperaría usted que el grupo pudiera hacer diferente en el futuro?<br>What would you hope the group could do differently in the future?	[texto]
7	¿Qué cosas de las que aprendió en las clases cree usted que puede llevar a cabo en su vida diaria?<br>What things that you learned in the classes do you think you can accomplish in your daily life?	[texto]
8	¿Qué cosas de las que aprendió en las clases cree uste que puede llevar a cabo con su comunidad?<br>What things that you learned in the classes do you think you can accomplish with your community?	[texto]
9	¿Qué cosas de las que aprendió en las clases, será más difícil de llevar a cabo en su vida diaria, pero quiere intentarlo? ¿Por qué?<br>What activities that you learned from the classes would be most difficult to practice in daily life, but you want to try to do it? Why?	[texto]<br>[texto]
10	¿Qué cosas de las que aprendió en las clases, será más difícil de llevar a cabo con su comunidad, pero quiere [intentarlo]? ¿Por qué?<br>What activities that you learned from the classes would be most difficult to practice in your community, [but you want to try to do it]? Why?	[texto]<br>[texto]

Post-test

Instrucciones

Esta encuesta sobre el manejo de los desechos sólidos o basura, será leída y entregada personalmente para esclarecer dudas. No existen respuestas falsas o incorrectas. Cada respuesta debe reflejar las percepciones, experiencias y conocimientos del participante. Hay **preguntas cerradas**, en las cuales se ofrecen respuestas opcionales, y **preguntas abiertas,** en las cuales los participantes deben pensar y escribir sus respuestas. La respuesta "no sé" es posible. El material deberá ser entregado en la primera sesión. De no hacerlo, no se tomará en cuenta la asistencia para el primer módulo. Tiempo aproximado de trabajo **30 minutos.**

1. ¿Qué significa para usted un "buen manejo de los desechos sólidos"?

2. ¿Qué hacen con la basura en su casa? (Puede elegir más de una opción)

- Quemo toda
- Quemo alguna, por ejemplo:
- Entierro todo
- Entierro alguna, por ejemplo:
- La recoge un servicio de basura
- Separo alguna, por ejemplo:
- Guardo alguna, por ejemplo:

Otro:

3. ¿Qué basura es la que más ve tirada en el patio o alrededor de su casa?

4. ¿Qué basura es la que más ve tirada en las calles de su comunidad?

5. ¿Cuáles son los mayores problemas para poder hacer un buen manejo de los desechos sólidos?

6. ¿Cuál cree es la mejor forma de deshacerse de la basura? (Marcar solamente una opción)

- Quemar toda
- Quemar alguna, por ejemplo:
- Enterrar todo
- Enterrar alguna, por ejemplo:
- Que la recoja un servicio de basura
- Separar alguna, por ejemplo:
- Guardar alguna, por ejemplo:
- Otra:

7. ¿Qué tipo de problemas se dan si no se quema la basura?

- Provoca enfermedades como:
- Huele mal
- Hace que los lugares se vean mal
- Provoca que se encuentren insectos y roedores (como cucarachas, ratas, ratones)
- Otro:

8. ¿Qué entiende por "contaminación ambiental"?

9. ¿Qué consecuencias tiene la "contaminación ambiental" para su salud?

10. ¿De qué manera cree que usted y su familia contribuyen al problema de la basura plástica?

11. ¿Cómo cree que el problema de la basura plástica le afecta a usted y su familia?

12. ¿De qué manera cree que el problema de la basura plástica afecta a su comunidad?

13. ¿Cómo cree que la quema de basura plástica afecta al medio ambiente?

14. ¿Cree que usted podría reducir el problema de la basura plástica?

- SÍ ¿Cómo?
- NO ¿Por qué?

15. ¿Cuál es la mejor forma para evitar que la basura plástica llegue a los ríos, lagos y océanos?

16. ¿Qué se puede hacer para que las personas participen en actividades para solucionar el problema de plásticos en su hogar y comunidad?

17. ¿Qué se puede hacer para crear conciencia ambiental sobre el problema de plásticos en su hogar y comunidad?

Initial / Final Survey

Instructions

This survey on solid waste or garbage management will be read for clarification and handed out in person. There are no incorrect answers. Each response should reflect the participant's perceptions, experiences and knowledge. There are **closed questions,** in which optional answers are offered, and **open questions,** in which participants must think and write their answers. The answer ***I don't know*** is possible. The material must be handed in at the first session. If not, attendance will not be taken into account for the first module. Approximate time to complete: **30 minutes.**

1. What does ***good management of solid waste*** mean to you?

2. What do you do with the garbage in your house? (You can choose more than one option)

- I burn it all
- I burn some of it, for example:
- I bury it all
- I bury some of it, for example:
- It is picked up by a garbage service
- I separate some of it, for example:
- I keep some of it, for example:

Other:

3. What type of garbage do you see littered the most in the yard or around your home?

4. What type of garbage do you see littered the most on the streets in your community?

5. What are the biggest problems for good solid waste management?

6. What do you think is the best way to dispose of garbage?

- Burn it all
- Burn some of it, for example:
- Bury it all
- Bury some of it, for example:
- Have it picked up by a garbage service
- Separate some of it, for example:
- Save some of it, for example:
- Other:

7. What kind of problems occur if garbage is not burned?

- Causes diseases such as:
- Smells bad
- Makes places look bad
- Attracts insects and rodents (such as cockroaches, rats, mice)
- Other:

8. What does the phrase ***environmental pollution*** mean to you?

9. What are the consequences of "environmental pollution" for your health?

10. In what ways do you think you and your family contribute to the plastic waste problem?

11. How do you think the plastic waste problem affects you and your family?

12. How do you think the plastic waste problem affects your community?

13. How do you think the burning of plastic waste affects the environment?

14. Do you think you could reduce the problem of plastic waste?

- YES How?
- NO Why?

15. What is the best way to prevent plastic waste from reaching rivers, lakes and oceans?

16. What can be done to get people **involved in activities** to solve the plastics problem in their homes and communities?

17. What can you do to **spread environmental awareness** about the plastics problem in your home and community?
